# Correction to: Characterization of adaptation mechanisms in sorghum using a multireference back-cross nested association mapping design and envirotyping

**DOI:** 10.1093/genetics/iyae062

**Published:** 2024-04-29

This is a correction to: Vincent Garin, Chiaka Diallo, Mohamed Lamine Tékété, Korotimi Théra, Baptiste Guitton, Karim Dagno, Abdoulaye G Diallo, Mamoutou Kouressy, Willmar Leiser, Fred Rattunde, Ibrahima Sissoko, Aboubacar Touré, Baloua Nébié, Moussa Samaké, Jana Kholovà, Angélique Berger, Julien Frouin, David Pot, Michel Vaksmann, Eva Weltzien, Niaba Témé, Jean-François Rami, Characterization of adaptation mechanisms in sorghum using a multireference back-cross nested association mapping design and envirotyping, *Genetics*, Volume 226, Issue 4, April 2024, iyae003, https://doi.org/10.1093/genetics/iyae003

In the originally published version of this manuscript the author Angélique Berger was inadvertently omitted from the author list.

This error has been corrected.

