# Supplementary material for: Characterization of adaptation mechanisms in sorghum using a multireference back-cross nested association mapping design and envirotyping
Source: Genetics. 2024 Feb 21;226(4):iyae003. doi: 10.1093/genetics/iyae003 (PMC10990433; doi:10.1093/genetics/iyae003)
Supplement: iyae003_Supplementary_Data [file iyae003_supplementary_data.docx]

**Characterization of adaptation mechanisms in sorghum using a multi-reference back-cross nested association mapping design and envirotyping - Supplementary material**

Vincent Garin, Chiaka Diallo, Mohamed Tekete, Korotimi Thera, Baptiste Guitton, Karim Dagno, Abdoulaye Diallo, Mamoutou Kouressy, Willmar Leiser, Fred Rattunde, Ibrahima Sissoko, Aboubacar Toure, Baloua Nebie, Moussa Samake, Jana Kholova, Julien Frouin, David Pot, Michel Vaksmann, Eva Weltzien, Niaba Teme, Jean-Francois Rami

**Supplemental figures**

**Figure S1:** Illustration of the crossing scheme with each parent represented as a circle  (recurrent parents in white and donor parents in blue) and each cross as a line. The larger ellipses represent the “phenotyping experiments” encompassing the locations and the sowing dates.


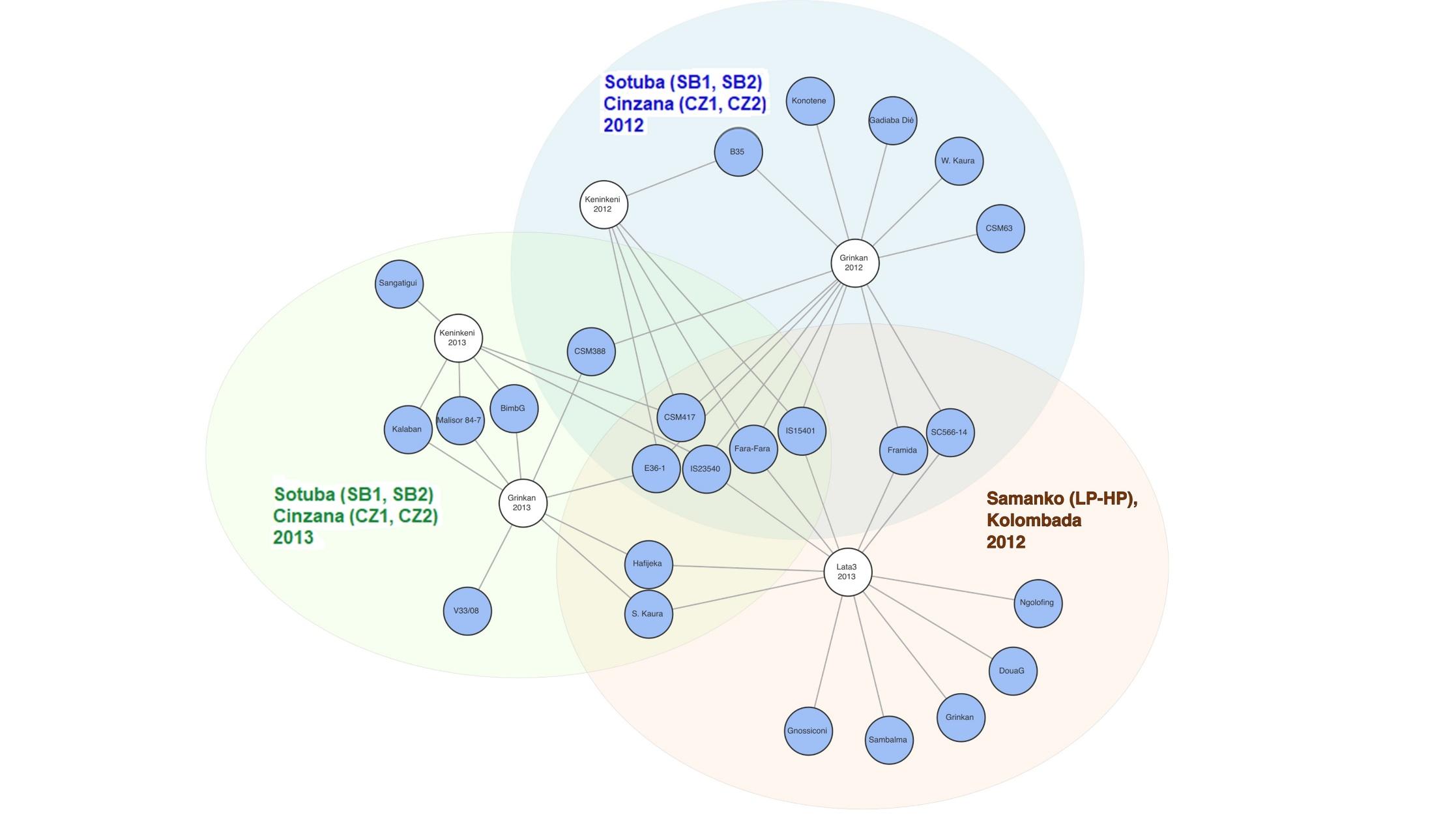


**Figure S2**: Field trial locations with environmental description in terms of temperature, precipitation, and photoperiod (daylength)


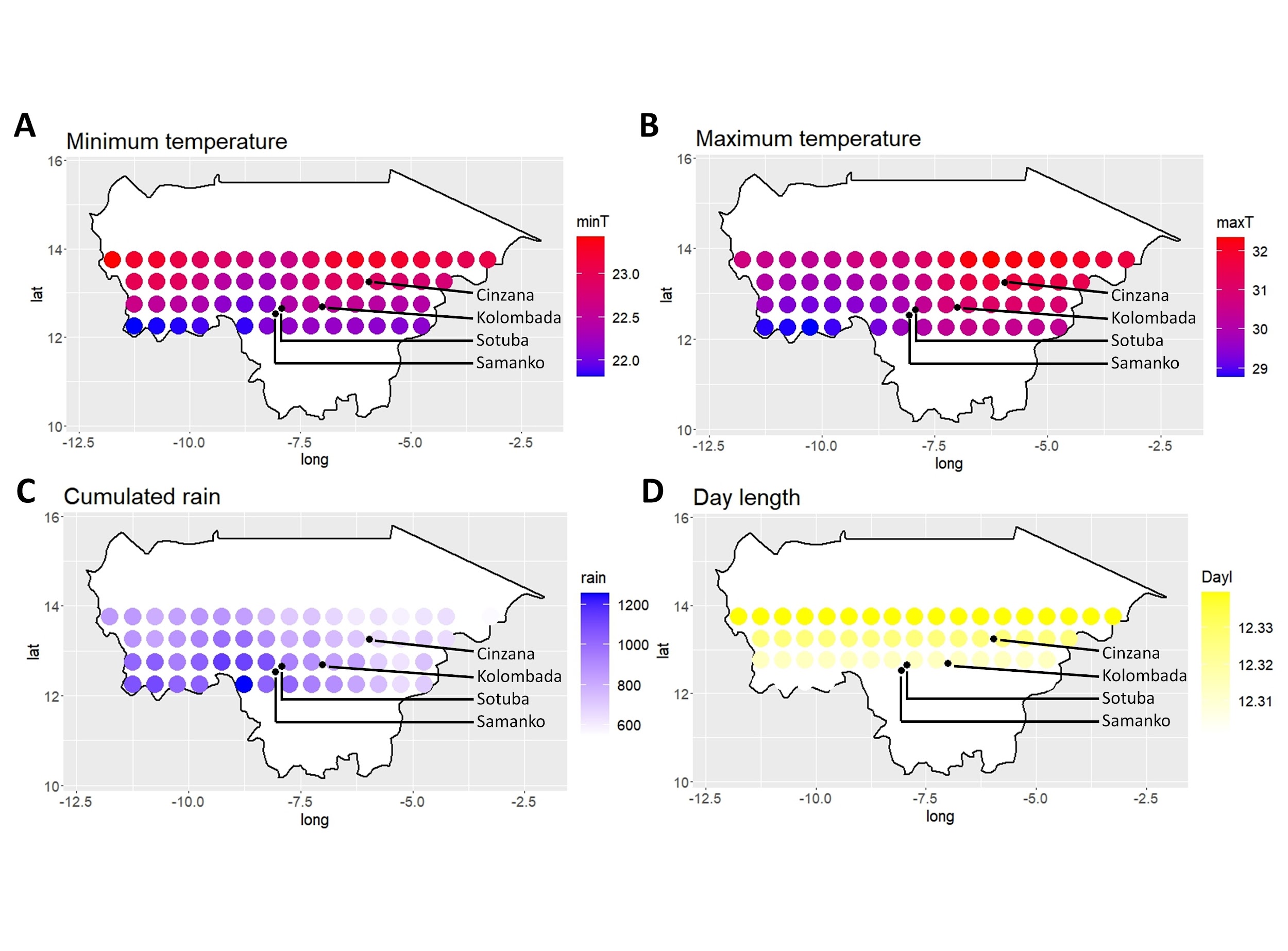


Environmental characterization of the field trial and neighbouring area over the growing season (June 20th to 20 Septembre 20th 2012-2013) given A) minimum temperature, B) maximum temperature, C) cumulated rain and D) day length using synthetic climatic data from Nasapower (Spark 2018)

**Figure S3**: Cinzana and Sotuba environments principal component analysis based on ECs


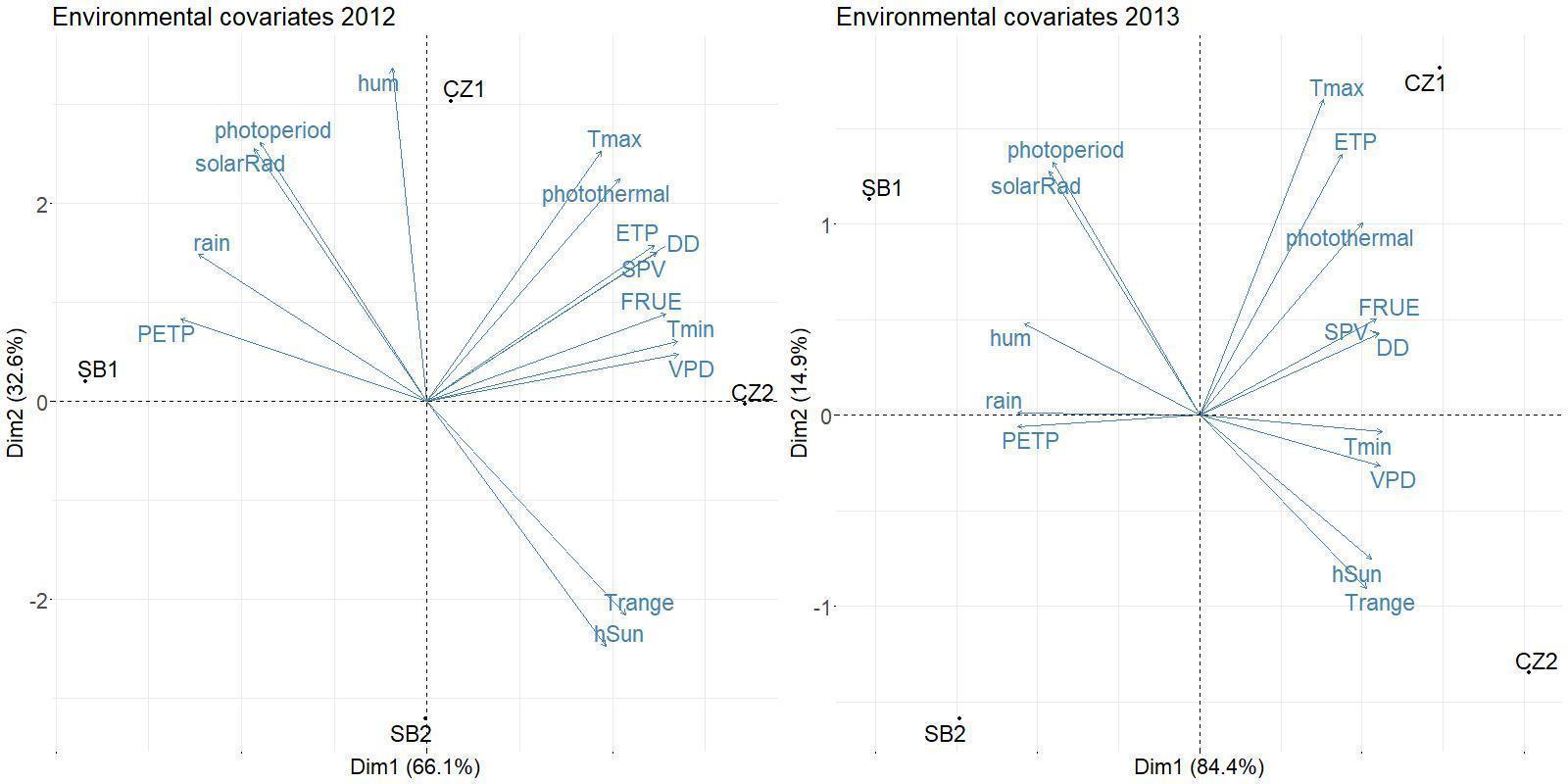


Four environments: Sotuba sowing 1 and 2 (SB1, SB2) and Cinzana (CZ1, CZ2). Environment covariables: cumulated rain over the season (rain), relative humidity (hum), vapour pressure deficit (VPD), slope of saturation VP curve (SVP), potential evapotranspiration (ETP), water deficit (PETP), minimum temperature (Tmin), maximum temperature (Tmax), temperature range (Trange), cumulated degree day (DD), temperature effect on radiation efficiency (FRUE), cumulated observed hour of sun (hsun), photoperiod, solar radiation (solarRad), photothermal (photoperiod * DD)

**Figure S4:** Principal component analysis based on the genetic information


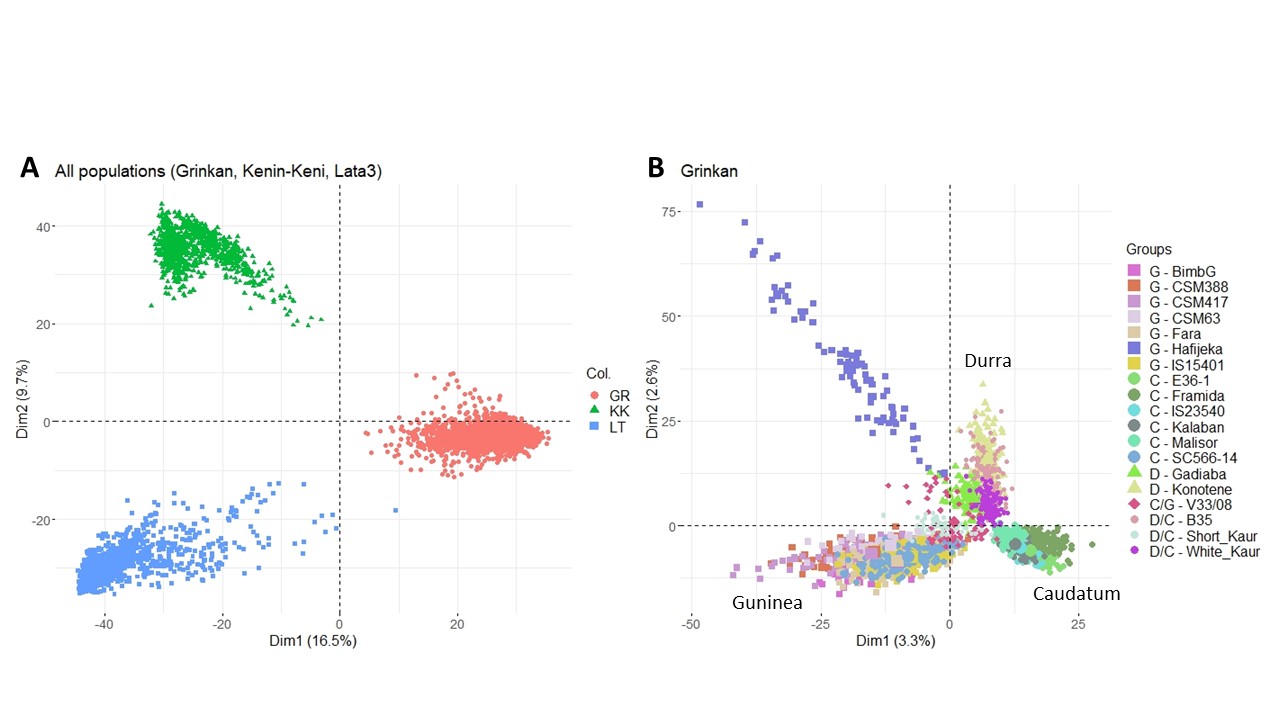


Principal component bi-plot performed on a subset of 5000 markers randomly selected of the three sub-populations Grinkan (GR), Kenin-Keni (KK) and Lata3 (LT) populations.

**Figure S5**: Distributions of the best linear unbiased estimates for the traits characterized on the BCNAM sub-populations and environments

**
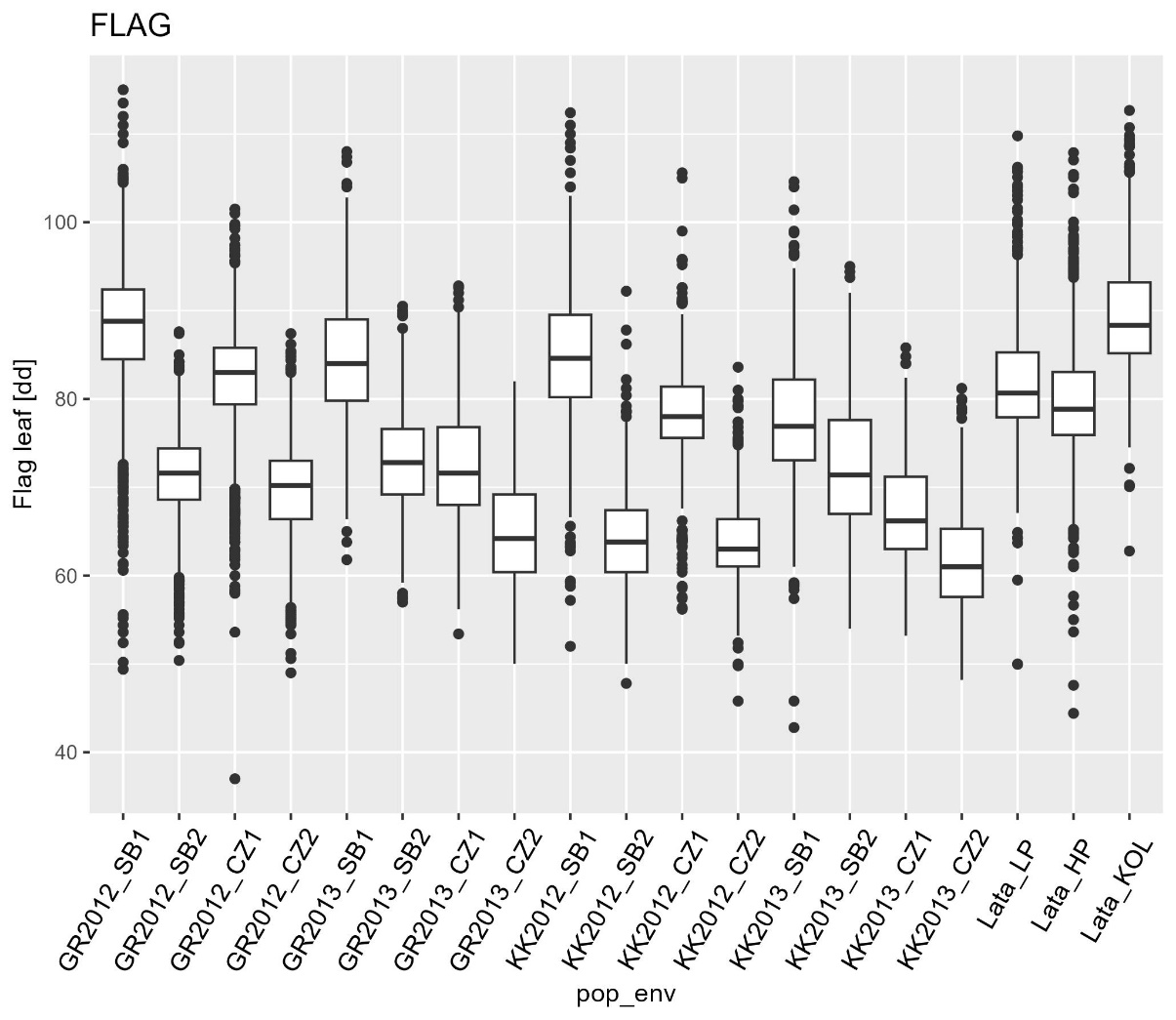
**

|  | **minimum** | **maximum** | **range** | **mean** | **std. dev.** |
| --- | --- | --- | --- | --- | --- |
| GR2012_SB1 | 49.4 | 115 | 65.6 | 88 | 7.4 |
| GR2012_SB2 | 50.4 | 87.6 | 37.2 | 71.4 | 4.9 |
| GR2012_CZ1 | 37 | 101.5 | 64.5 | 82.1 | 6.4 |
| GR2012_CZ2 | 49 | 87.4 | 38.4 | 69.7 | 5.5 |
| GR2013_SB1 | 61.8 | 108 | 46.2 | 84.3 | 7.4 |
| GR2013_SB2 | 57 | 90.5 | 33.5 | 72.8 | 5.7 |
| GR2013_CZ1 | 53.4 | 92.8 | 39.4 | 72.5 | 6.8 |
| GR2013_CZ2 | 50 | 82 | 32 | 64.8 | 5.8 |
| KK2012_SB1 | 52 | 112.4 | 60.4 | 84.9 | 8 |
| KK2012_SB2 | 47.8 | 92.2 | 44.4 | 64.3 | 6.1 |
| KK2012_CZ1 | 56.2 | 105.6 | 49.4 | 78.4 | 6.5 |
| KK2012_CZ2 | 45.8 | 83.6 | 37.8 | 63.8 | 5.1 |
| KK2013_SB1 | 42.8 | 104.6 | 61.8 | 77.5 | 8.1 |
| KK2013_SB2 | 54 | 95 | 41 | 72.6 | 7.8 |
| KK2013_CZ1 | 53.2 | 85.8 | 32.6 | 67.6 | 6.3 |
| KK2013_CZ2 | 48.2 | 81.2 | 33 | 61.8 | 6.3 |
| Lata_LP | 50 | 109.8 | 59.8 | 81.9 | 6.9 |
| Lata_HP | 44.4 | 107.9 | 63.5 | 79.8 | 7.3 |
| Lata_KOL | 62.8 | 112.7 | 49.9 | 89.6 | 6.6 |
| **average** | **50.8** | **97.7** | **46.9** | **75.1** | **6.6** |

**
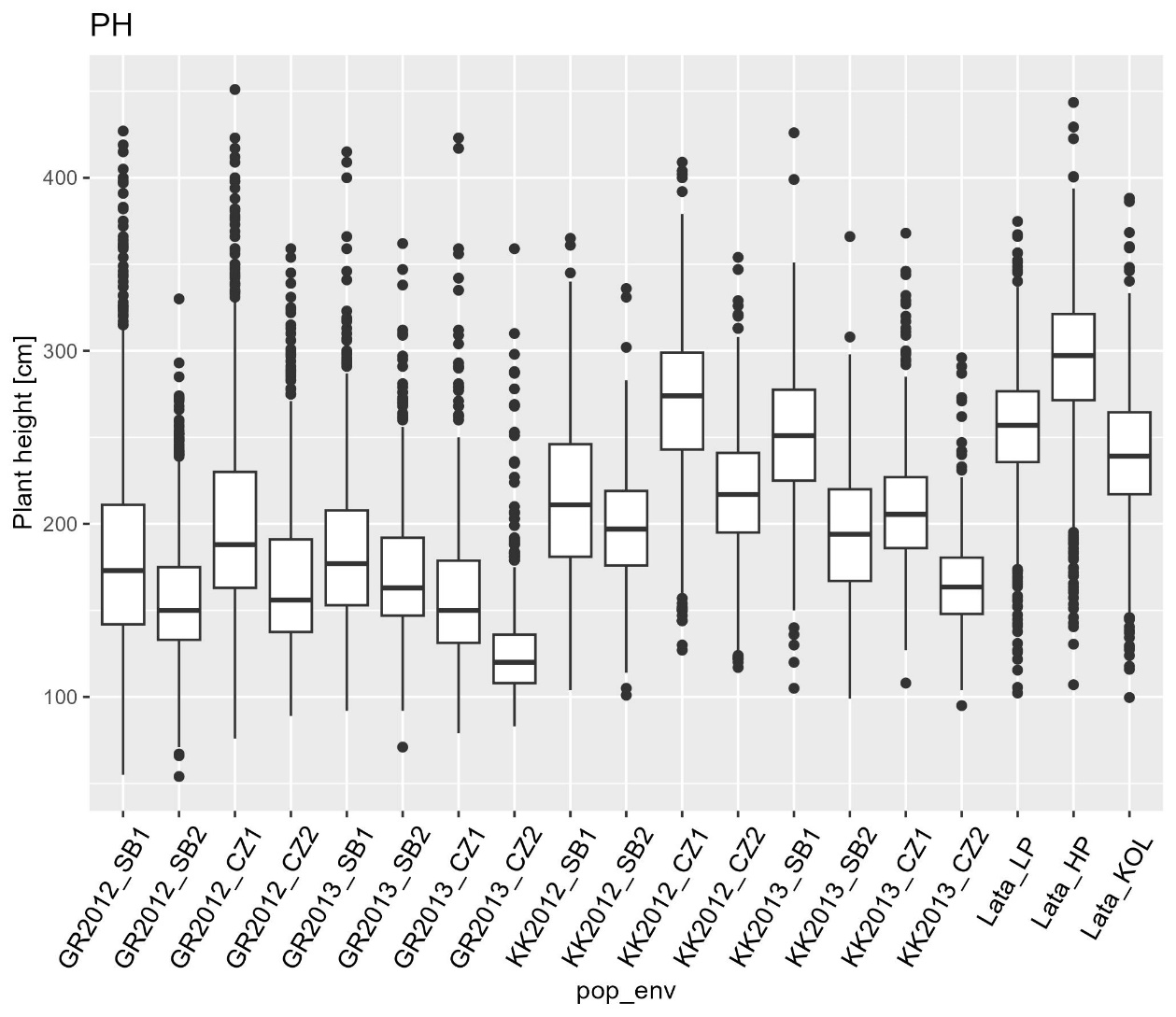
**

|  | **minimum** | **maximum** | **range** | **mean** | **std. dev.** |
| --- | --- | --- | --- | --- | --- |
| GR2012_SB1 | 55 | 427 | 372 | 182 | 58 |
| GR2012_SB2 | 54 | 330 | 276 | 156.8 | 35.2 |
| GR2012_CZ1 | 76 | 451 | 375 | 201 | 54.9 |
| GR2012_CZ2 | 89 | 359 | 270 | 168.1 | 43.4 |
| GR2013_SB1 | 92 | 415 | 323 | 186.9 | 49.3 |
| GR2013_SB2 | 71 | 362 | 291 | 173.6 | 41.1 |
| GR2013_CZ1 | 79 | 423 | 344 | 160.4 | 44.4 |
| GR2013_CZ2 | 83 | 359 | 276 | 128.2 | 34.1 |
| KK2012_SB1 | 104 | 365 | 261 | 213 | 50.3 |
| KK2012_SB2 | 101 | 336 | 235 | 198.3 | 33.3 |
| KK2012_CZ1 | 127 | 409 | 282 | 268.2 | 47.9 |
| KK2012_CZ2 | 117 | 354 | 237 | 218.3 | 38.6 |
| KK2013_SB1 | 105 | 426 | 321 | 248.9 | 45 |
| KK2013_SB2 | 99 | 366 | 267 | 194.1 | 39.4 |
| KK2013_CZ1 | 108 | 368 | 260 | 209.1 | 41.2 |
| KK2013_CZ2 | 95 | 296 | 201 | 167.4 | 31.4 |
| Lata_LP | 102.2 | 374.8 | 272.5 | 254.4 | 37.3 |
| Lata_HP | 107 | 443.5 | 336.5 | 293.1 | 43.7 |
| Lata_KOL | 99.6 | 388.2 | 288.5 | 238.9 | 38.6 |
| **average** | **92.8** | **381.7** | **288.9** | **203.2** | **42.5** |

**
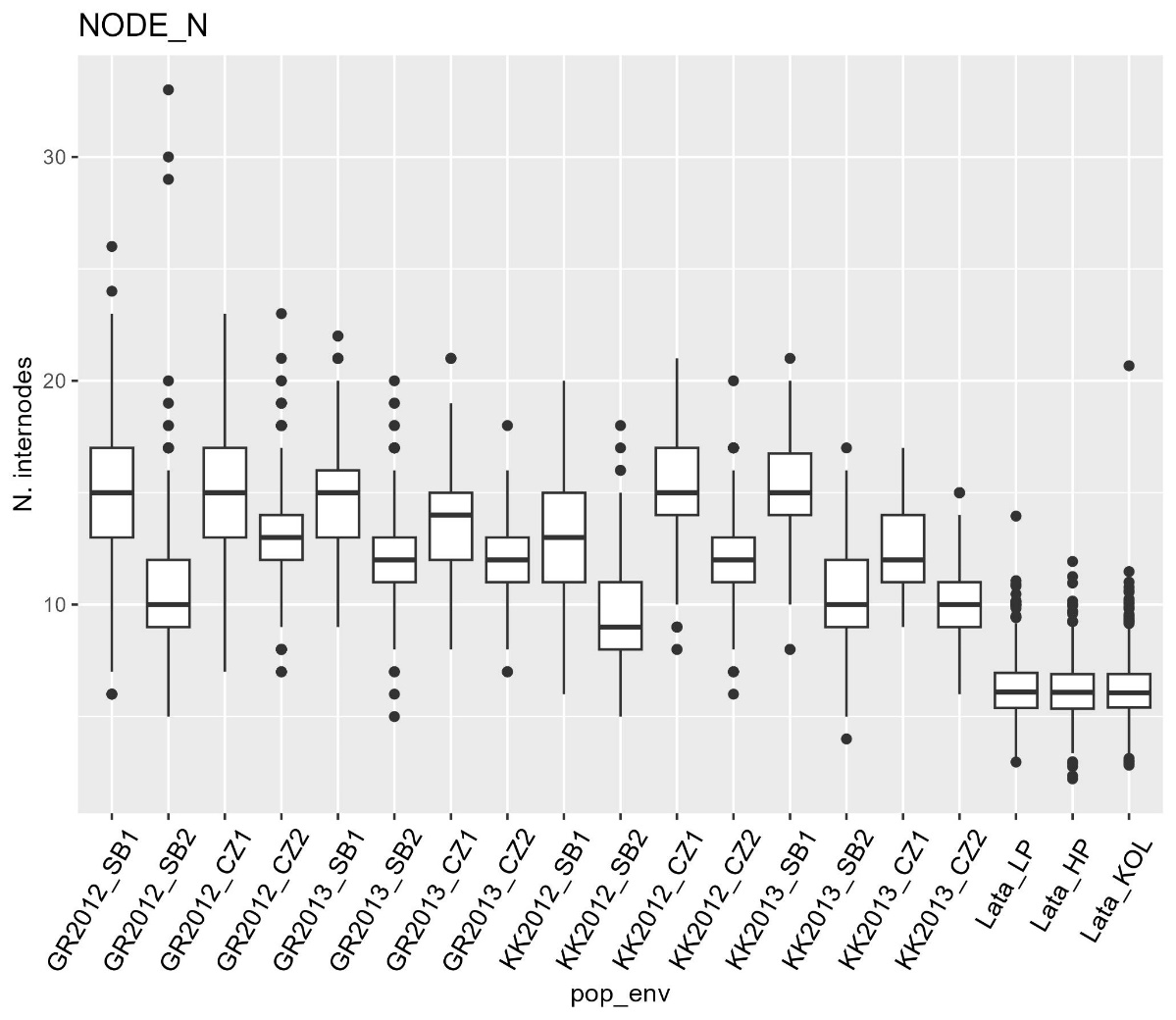
**

|  | **minimum** | **maximum** | **range** | **mean** | **std. dev.** |
| --- | --- | --- | --- | --- | --- |
| GR2012_SB1 | 6 | 26 | 20 | 14.7 | 2.8 |
| GR2012_SB2 | 5 | 33 | 28 | 10.5 | 2.5 |
| GR2012_CZ1 | 7 | 23 | 16 | 15 | 2.5 |
| GR2012_CZ2 | 7 | 23 | 16 | 13 | 2.1 |
| GR2013_SB1 | 9 | 22 | 13 | 14.9 | 2.3 |
| GR2013_SB2 | 5 | 20 | 15 | 11.9 | 2.1 |
| GR2013_CZ1 | 8 | 21 | 13 | 13.8 | 2 |
| GR2013_CZ2 | 7 | 18 | 11 | 11.5 | 1.5 |
| KK2012_SB1 | 6 | 20 | 14 | 12.8 | 2.7 |
| KK2012_SB2 | 5 | 18 | 13 | 9.3 | 2.3 |
| KK2012_CZ1 | 8 | 21 | 13 | 15.4 | 2.1 |
| KK2012_CZ2 | 6 | 20 | 14 | 11.9 | 2 |
| KK2013_SB1 | 8 | 21 | 13 | 15 | 2.2 |
| KK2013_SB2 | 4 | 17 | 13 | 10.2 | 2.3 |
| KK2013_CZ1 | 9 | 17 | 8 | 12.5 | 1.8 |
| KK2013_CZ2 | 6 | 15 | 9 | 10.2 | 1.8 |
| Lata_LP | 3 | 14 | 11 | 6.2 | 1.2 |
| Lata_HP | 2.2 | 11.9 | 9.7 | 6.2 | 1.2 |
| Lata_KOL | 2.8 | 20.7 | 17.8 | 6.2 | 1.4 |
| **average** | **6** | **20.1** | **14.1** | **11.7** | **2** |

**
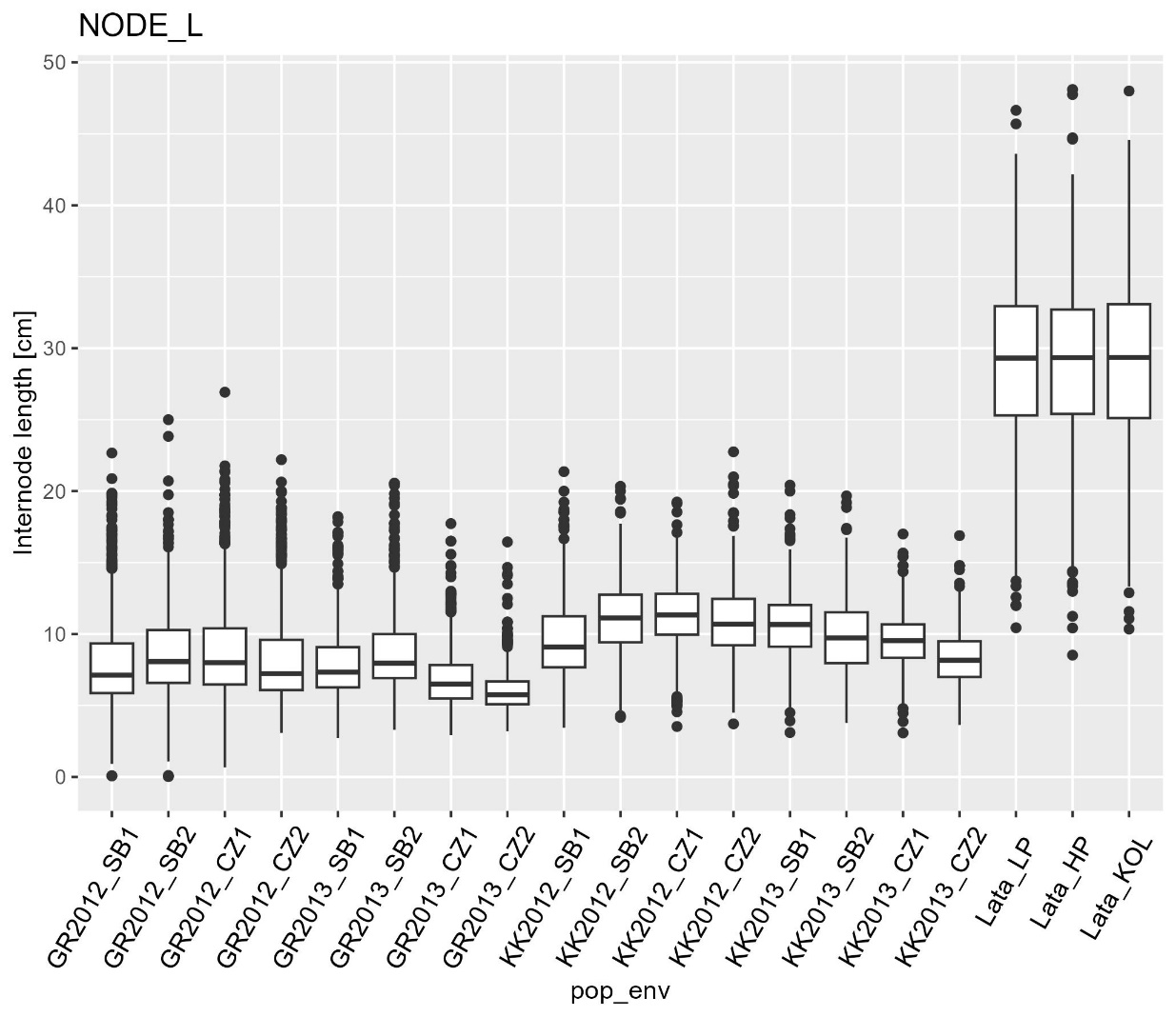
**

|  | **minimum** | **maximum** | **range** | **mean** | **std. dev.** |
| --- | --- | --- | --- | --- | --- |
| GR2012_SB1 | 0.1 | 22.7 | 22.6 | 7.9 | 3.1 |
| GR2012_SB2 | 0 | 25 | 25 | 8.6 | 2.8 |
| GR2012_CZ1 | 0.7 | 26.9 | 26.3 | 8.8 | 3.2 |
| GR2012_CZ2 | 3.1 | 22.2 | 19.1 | 8.2 | 3 |
| GR2013_SB1 | 2.7 | 18.2 | 15.5 | 8 | 2.6 |
| GR2013_SB2 | 3.3 | 20.6 | 17.3 | 8.8 | 2.9 |
| GR2013_CZ1 | 2.9 | 17.7 | 14.8 | 6.9 | 2.1 |
| GR2013_CZ2 | 3.2 | 16.4 | 13.2 | 6.1 | 1.6 |
| KK2012_SB1 | 3.4 | 21.4 | 17.9 | 9.6 | 2.9 |
| KK2012_SB2 | 4.2 | 20.3 | 16.2 | 11.2 | 2.8 |
| KK2012_CZ1 | 3.5 | 19.2 | 15.7 | 11.3 | 2.4 |
| KK2012_CZ2 | 3.7 | 22.8 | 19 | 10.8 | 2.8 |
| KK2013_SB1 | 3.1 | 20.4 | 17.3 | 10.7 | 2.6 |
| KK2013_SB2 | 3.8 | 19.7 | 15.9 | 9.8 | 2.7 |
| KK2013_CZ1 | 3.1 | 17 | 13.9 | 9.5 | 2.1 |
| KK2013_CZ2 | 3.6 | 16.9 | 13.3 | 8.4 | 2.1 |
| Lata_LP | 10.4 | 46.6 | 36.2 | 29 | 5.9 |
| Lata_HP | 8.5 | 48.1 | 39.6 | 29 | 5.6 |
| Lata_KOL | 10.4 | 48 | 37.6 | 29 | 5.8 |
| **average** | **3.9** | **24.7** | **20.9** | **12.2** | **3.1** |

**
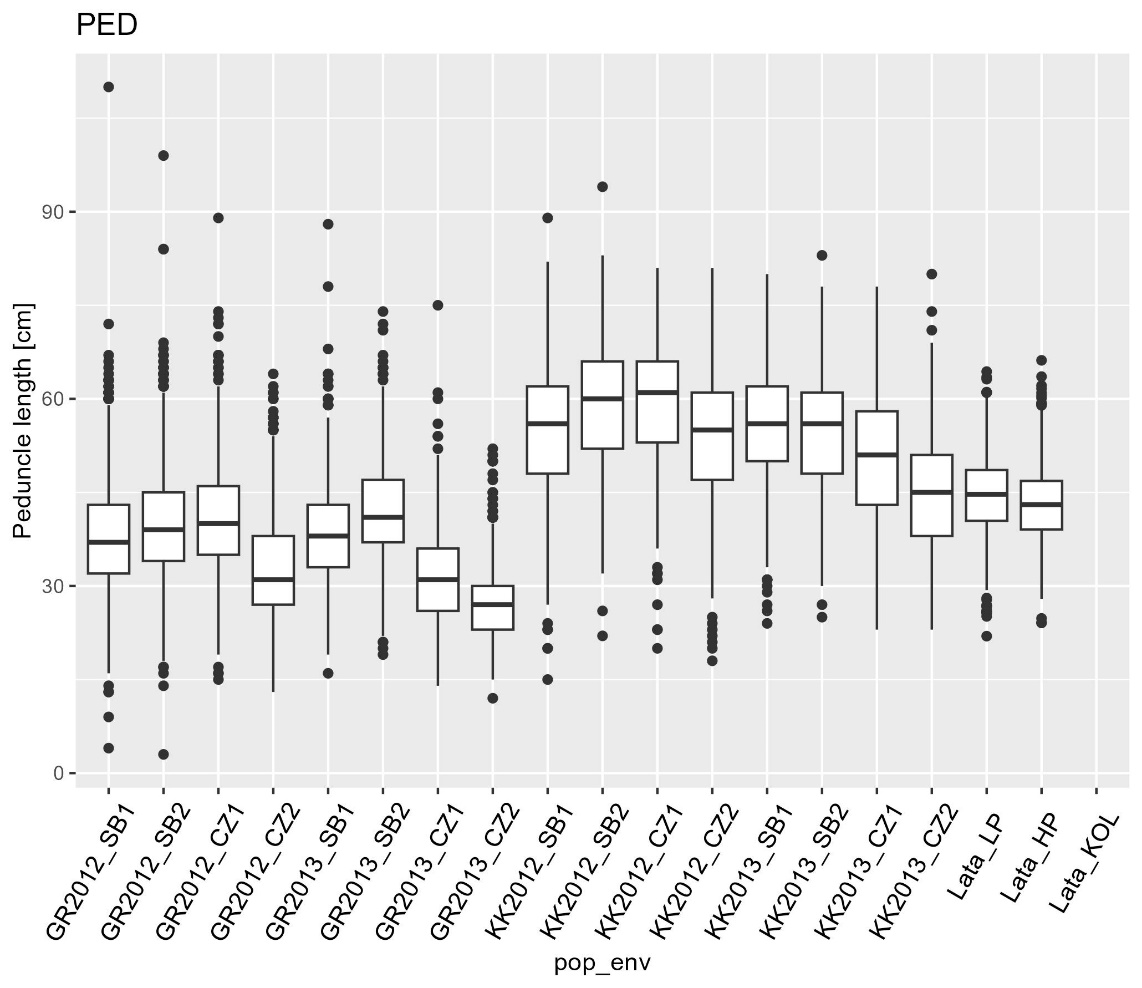
**

|  | **minimum** | **maximum** | **range** | **mean** | **std. dev.** |
| --- | --- | --- | --- | --- | --- |
| GR2012_SB1 | 4 | 110 | 106 | 37.8 | 9.4 |
| GR2012_SB2 | 3 | 99 | 96 | 40.1 | 9.2 |
| GR2012_CZ1 | 15 | 89 | 74 | 40.9 | 9.1 |
| GR2012_CZ2 | 13 | 64 | 51 | 32.8 | 8.2 |
| GR2013_SB1 | 16 | 88 | 72 | 38.6 | 8.6 |
| GR2013_SB2 | 19 | 74 | 55 | 42.4 | 8.7 |
| GR2013_CZ1 | 14 | 75 | 61 | 31.3 | 8 |
| GR2013_CZ2 | 12 | 52 | 40 | 27.5 | 6.4 |
| KK2012_SB1 | 15 | 89 | 74 | 55 | 10.8 |
| KK2012_SB2 | 22 | 94 | 72 | 58.8 | 9.6 |
| KK2012_CZ1 | 20 | 81 | 61 | 59.1 | 9.9 |
| KK2012_CZ2 | 18 | 81 | 63 | 53.8 | 11.1 |
| KK2013_SB1 | 24 | 80 | 56 | 55.2 | 10.1 |
| KK2013_SB2 | 25 | 83 | 58 | 54.8 | 9.8 |
| KK2013_CZ1 | 23 | 78 | 55 | 50.7 | 10.9 |
| KK2013_CZ2 | 23 | 80 | 57 | 45.2 | 9.9 |
| Lata_LP | 22 | 64.4 | 42.4 | 44.5 | 6.3 |
| Lata_HP | 24.1 | 66.2 | 42.1 | 43.2 | 6.1 |
| Lata_KOL |  |  |  |  |  |
| **average** | **16.9** | **82.3** | **65.7** | **55** | **8** |

**
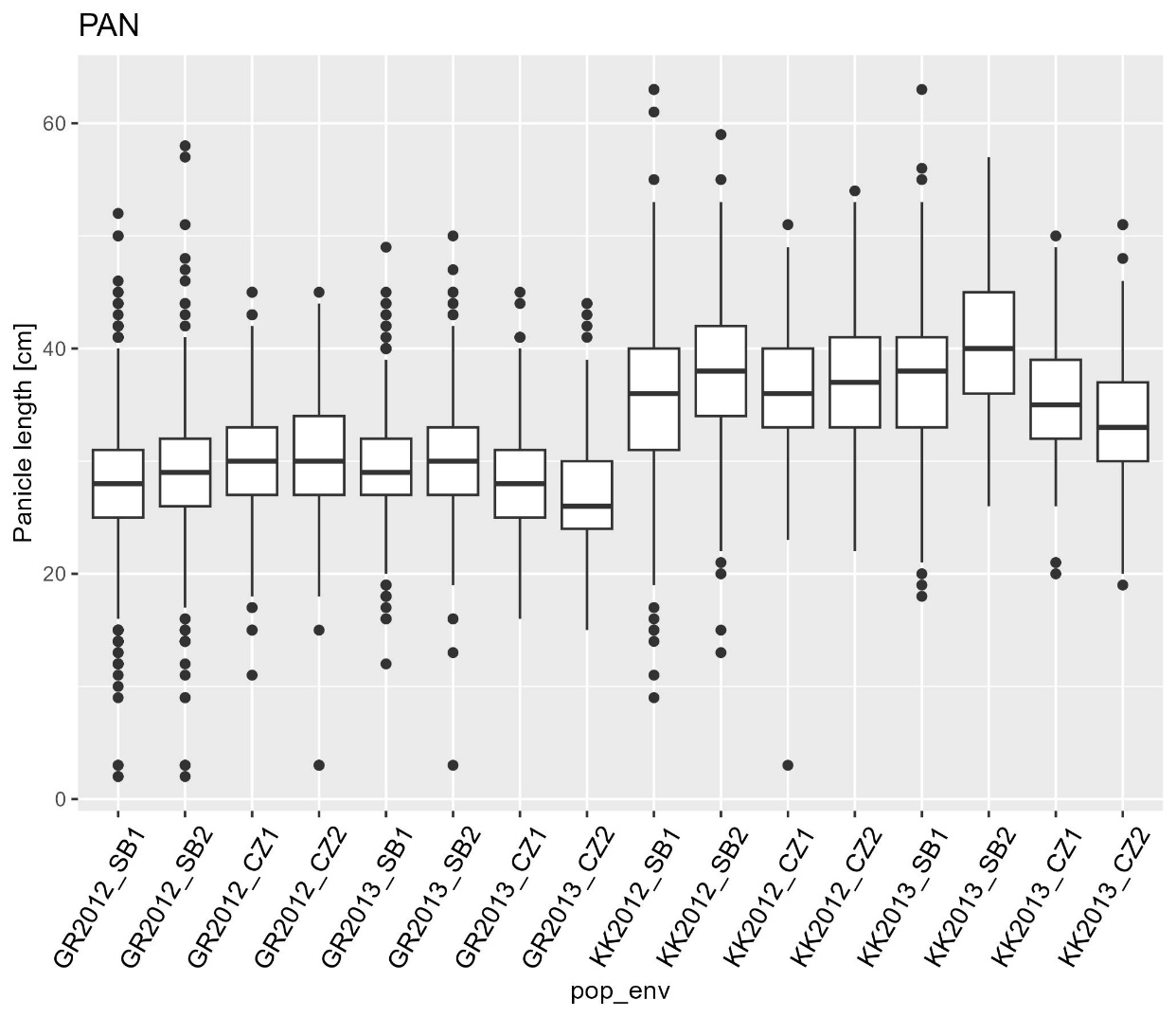
**

|  | **minimum** | **maximum** | **range** | **mean** | **std. dev.** |
| --- | --- | --- | --- | --- | --- |
| GR2012_SB1 | 2 | 52 | 50 | 28 | 5.6 |
| GR2012_SB2 | 2 | 58 | 56 | 28.9 | 4.9 |
| GR2012_CZ1 | 11 | 45 | 34 | 29.9 | 4.3 |
| GR2012_CZ2 | 3 | 45 | 42 | 30.3 | 4.8 |
| GR2013_SB1 | 12 | 49 | 37 | 29.6 | 4.9 |
| GR2013_SB2 | 3 | 50 | 47 | 30.4 | 5 |
| GR2013_CZ1 | 16 | 45 | 29 | 28.2 | 4.5 |
| GR2013_CZ2 | 15 | 44 | 29 | 27.4 | 4.6 |
| KK2012_SB1 | 9 | 63 | 54 | 35.4 | 7.3 |
| KK2012_SB2 | 13 | 59 | 46 | 38.1 | 6.5 |
| KK2012_CZ1 | 3 | 51 | 48 | 36 | 5.3 |
| KK2012_CZ2 | 22 | 54 | 32 | 37.2 | 5.8 |
| KK2013_SB1 | 18 | 63 | 45 | 37.4 | 6.1 |
| KK2013_SB2 | 26 | 57 | 31 | 40 | 6.7 |
| KK2013_CZ1 | 20 | 50 | 30 | 35.4 | 5 |
| KK2013_CZ2 | 19 | 51 | 32 | 33.7 | 5.3 |
| Lata_LP |  |  |  |  |  |
| Lata_HP |  |  |  |  |  |
| Lata_KOL |  |  |  |  |  |
| **average** | **12.1** | **52.3** | **40.1** | **32.9** | **5.4** |

**
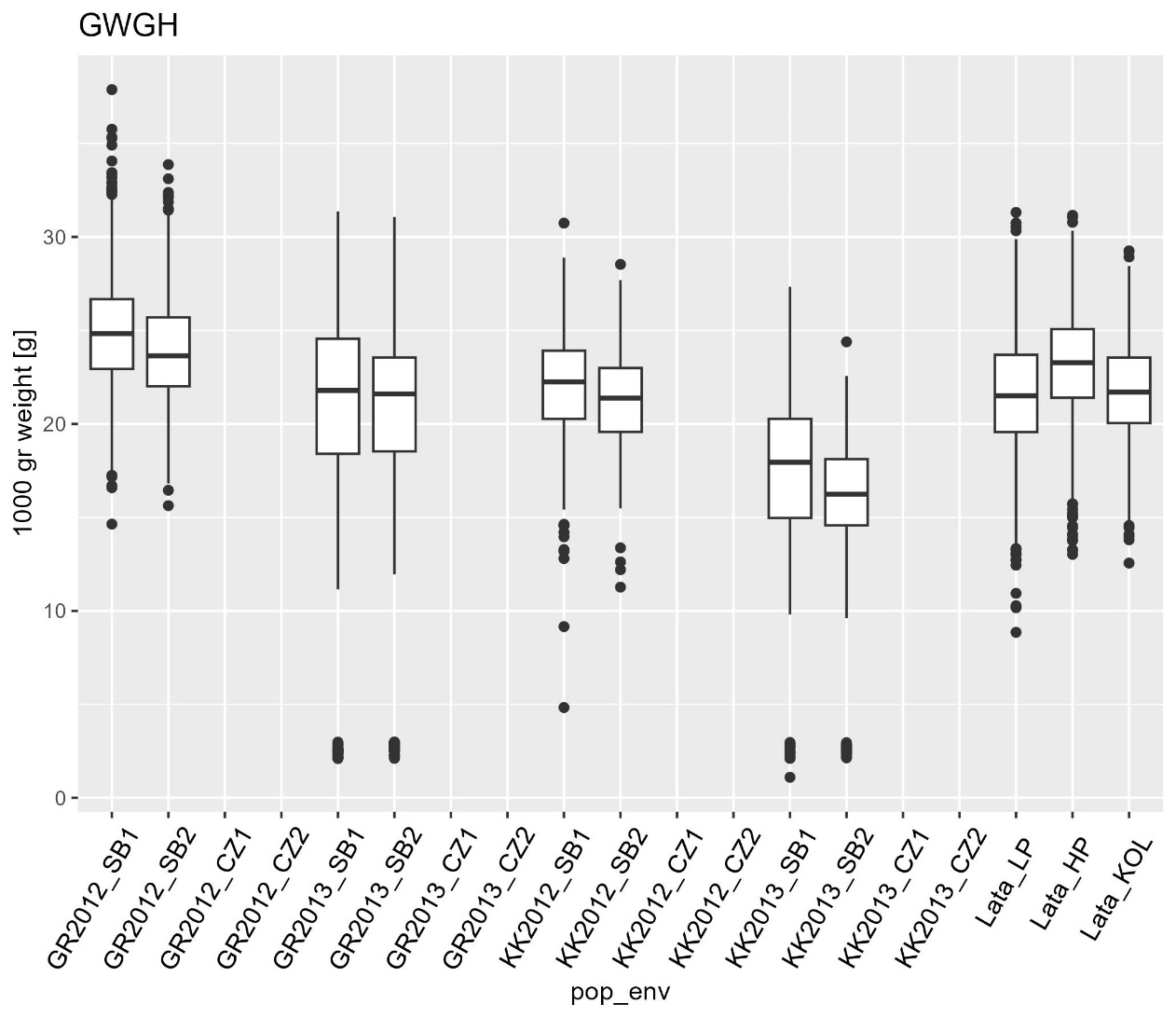
**

|  | **minimum** | **maximum** | **range** | **mean** | **std. dev.** |
| --- | --- | --- | --- | --- | --- |
| GR2012_SB1 | 14.6 | 37.9 | 23.2 | 24.9 | 3 |
| GR2012_SB2 | 15.6 | 33.9 | 18.2 | 23.9 | 2.7 |
| GR2012_CZ1 |  |  |  |  |  |
| GR2012_CZ2 |  |  |  |  |  |
| GR2013_SB1 | 2.1 | 31.4 | 29.3 | 20.6 | 6.4 |
| GR2013_SB2 | 2.1 | 31.1 | 29 | 20 | 6.5 |
| GR2013_CZ1 |  |  |  |  |  |
| GR2013_CZ2 |  |  |  |  |  |
| KK2012_SB1 | 4.8 | 30.7 | 25.9 | 22 | 2.9 |
| KK2012_SB2 | 11.3 | 28.5 | 17.3 | 21.3 | 2.6 |
| KK2012_CZ1 |  |  |  |  |  |
| KK2012_CZ2 |  |  |  |  |  |
| KK2013_SB1 | 1.1 | 27.3 | 26.2 | 16.6 | 6.1 |
| KK2013_SB2 | 2.1 | 24.4 | 22.2 | 14.8 | 5.5 |
| KK2013_CZ1 |  |  |  |  |  |
| KK2013_CZ2 |  |  |  |  |  |
| Lata_LP | 8.9 | 31.3 | 22.5 | 21.5 | 3.2 |
| Lata_HP | 13 | 31.2 | 18.1 | 23.1 | 3 |
| Lata_KOL | 12.6 | 29.3 | 16.7 | 21.8 | 2.7 |
| **average** | **8** | **30.6** | **22.6** | **21** | **4.1** |

**
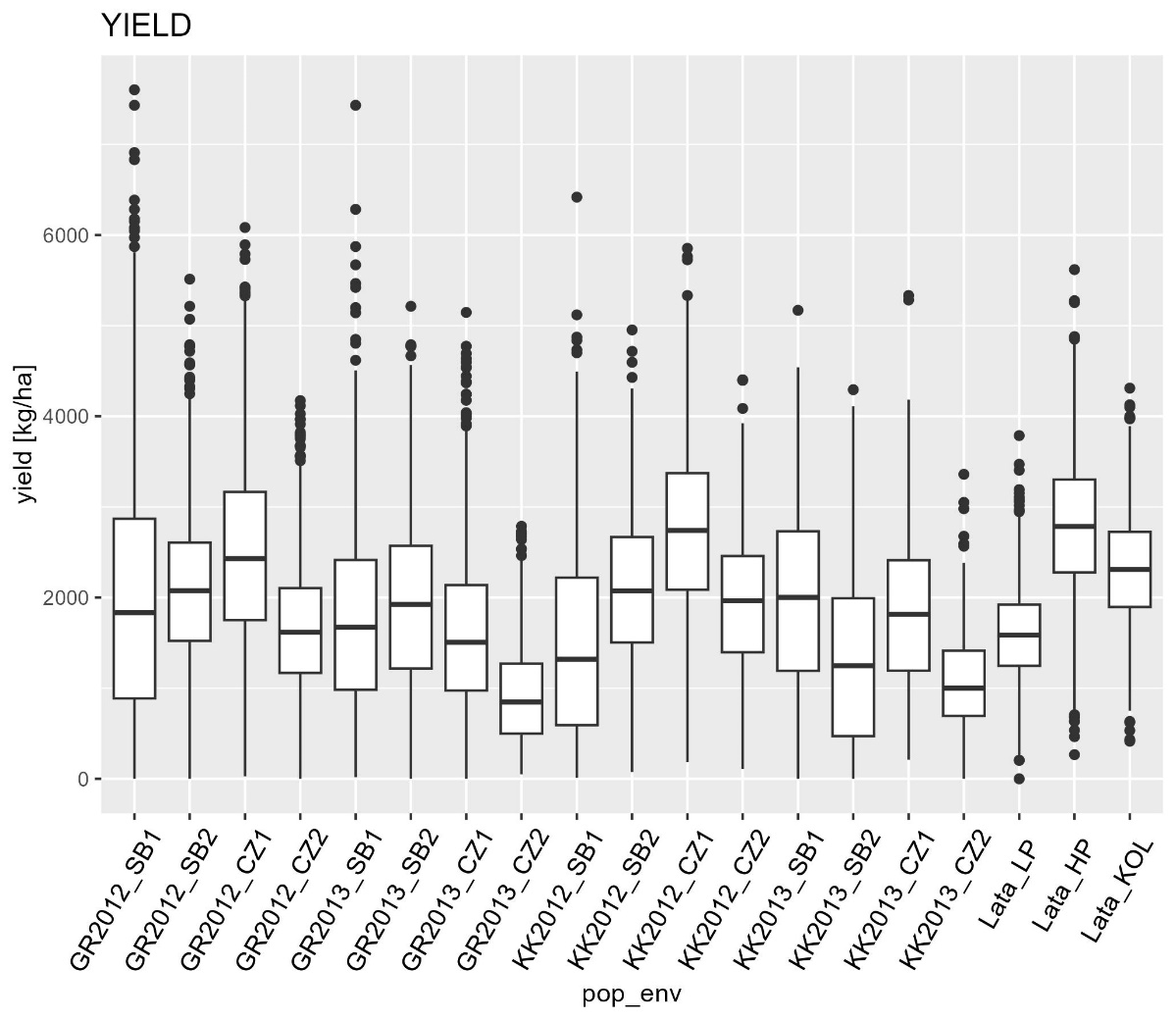
**

|  | **minimum** | **maximum** | **range** | **mean** | **std. dev.** |
| --- | --- | --- | --- | --- | --- |
| GR2012_SB1 | 0.7 | 7603 | 7602.3 | 2020.2 | 1374.6 |
| GR2012_SB2 | 0.2 | 5513.3 | 5513.2 | 2093.9 | 833.7 |
| GR2012_CZ1 | 26.7 | 6083.3 | 6056.7 | 2483.3 | 1028.7 |
| GR2012_CZ2 | 0 | 4173.3 | 4173.3 | 1659.8 | 726.7 |
| GR2013_SB1 | 18.3 | 7432 | 7413.7 | 1772.9 | 1069.2 |
| GR2013_SB2 | 0 | 5214 | 5214 | 1925.4 | 996 |
| GR2013_CZ1 | 0 | 5146.7 | 5146.7 | 1656.4 | 995.6 |
| GR2013_CZ2 | 50 | 2786.7 | 2736.7 | 920 | 554 |
| KK2012_SB1 | 10.7 | 6418.3 | 6407.7 | 1522.1 | 1131.7 |
| KK2012_SB2 | 73.7 | 4953 | 4879.3 | 2127.4 | 887.2 |
| KK2012_CZ1 | 186.7 | 5853.3 | 5666.7 | 2777.7 | 1006.1 |
| KK2012_CZ2 | 110 | 4403.3 | 4293.3 | 1963.3 | 770.1 |
| KK2013_SB1 | 0 | 5169.7 | 5169.7 | 1971.6 | 1063.1 |
| KK2013_SB2 | 0 | 4293.7 | 4293.7 | 1344.9 | 1005.9 |
| KK2013_CZ1 | 210 | 5333.3 | 5123.3 | 1878.5 | 933.2 |
| KK2013_CZ2 | 0 | 3360 | 3360 | 1106.3 | 596 |
| Lata_LP | 0 | 3787.1 | 3787.1 | 1598.5 | 537.8 |
| Lata_HP | 266.4 | 5618.5 | 5352.1 | 2772.5 | 821.3 |
| Lata_KOL | 414.7 | 4310.8 | 3896.1 | 2316 | 633.4 |
| **average** | **72** | **5129.1** | **5057.1** | **1890** | **892.9** |

**Figure S6:** Principal component analysis plots of the phenotypic traits for each population x environment

***Grinkan 2012***


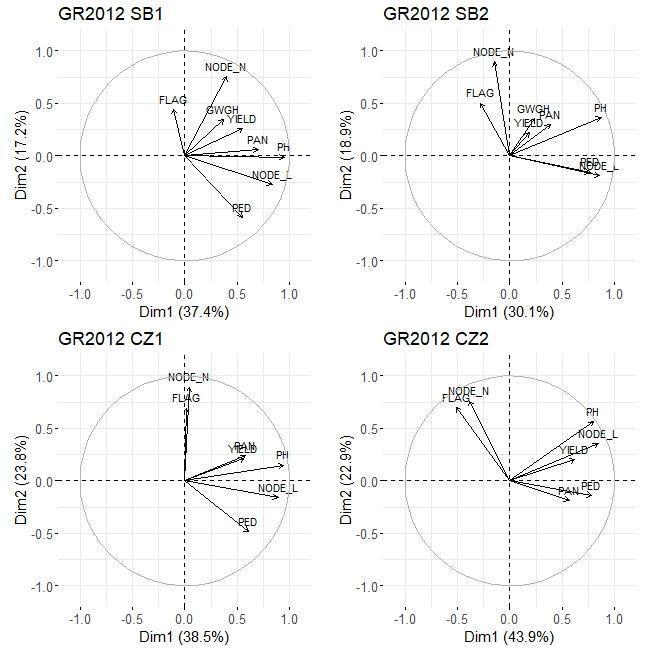


Principal component analysis plots of the adjusted mean phenotypic traits (BLUEs) in the four environments where Grinkan population part was phenotyped in 2012 (SB1, 2: Sotuba sowing 1 and 2; CZ1, 2: Cinzana sowing 1 and 2) FLAG: flag leaf appearance, PH: plant height, NODE_N: number of nodes, NODE_L: average length of the internode, PED: peduncle length, PAN: panicle length, GWGH: 1000 grain weight, YIELD: grain yield

***Grinkan 2013***


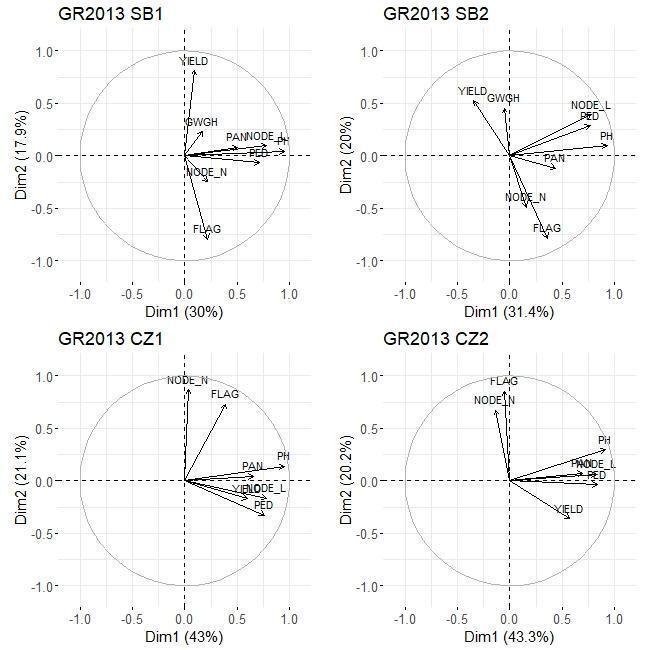


Principal component analysis plots of the adjusted mean phenotypic traits (BLUEs) in the four environments where Grinkan population part was phenotyped in 2013 (SB1, 2: Sotuba sowing 1 and 2; CZ1, 2: Cinzana sowing 1 and 2) FLAG: flag leaf appearance, PH: plant height, NODE_N: number of nodes, NODE_L: average length of the internode, PED: peduncle length, PAN: panicle length, GWGH: 1000 grain weight, YIELD: grain yield

***Kenin-Keni 2012***

**
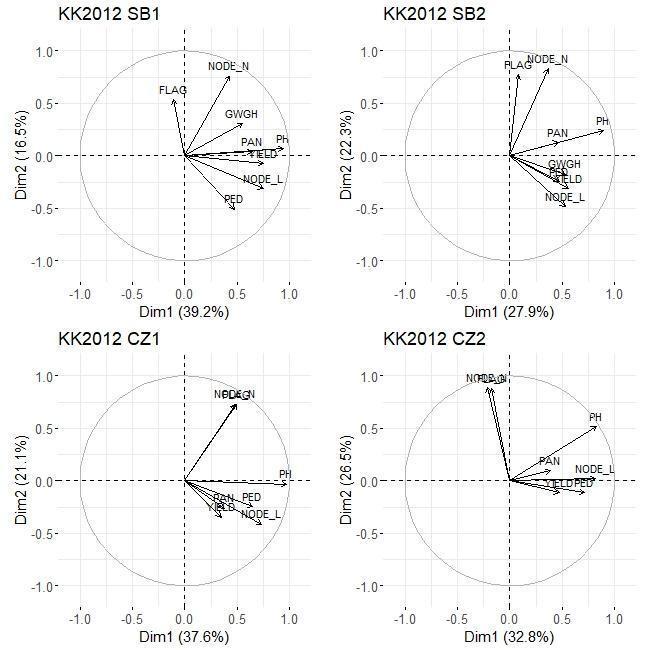
**

Principal component analysis plots of the adjusted mean phenotypic traits (BLUEs) in the four environments where Kenin-Keni population part was phenotyped in 2012 (SB1, 2: Sotuba sowing 1 and 2; CZ1, 2: Cinzana sowing 1 and 2) FLAG: flag leaf appearance, PH: plant height, NODE_N: number of nodes, NODE_L: average length of the internode, PED: peduncle length, PAN: panicle length, GWGH: 1000 grain weight, YIELD: grain yield

***Kenin-Keni 2013***


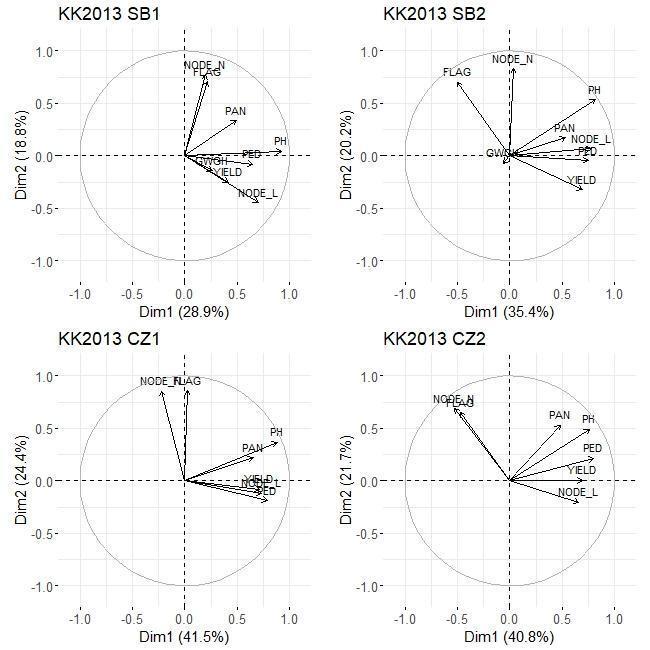


Principal component analysis plots of the adjusted mean phenotypic traits (BLUEs) in the four environments where Kenin-Keni population part was phenotyped in 2013 (SB1, 2: Sotuba sowing 1 and 2; CZ1, 2: Cinzana sowing 1 and 2) FLAG: flag leaf appearance, PH: plant height, NODE_N: number of nodes, NODE_L: average length of the internode, PED: peduncle length, PAN: panicle length, GWGH: 1000 grain weight, YIELD: grain yield

***Lata (2013)***


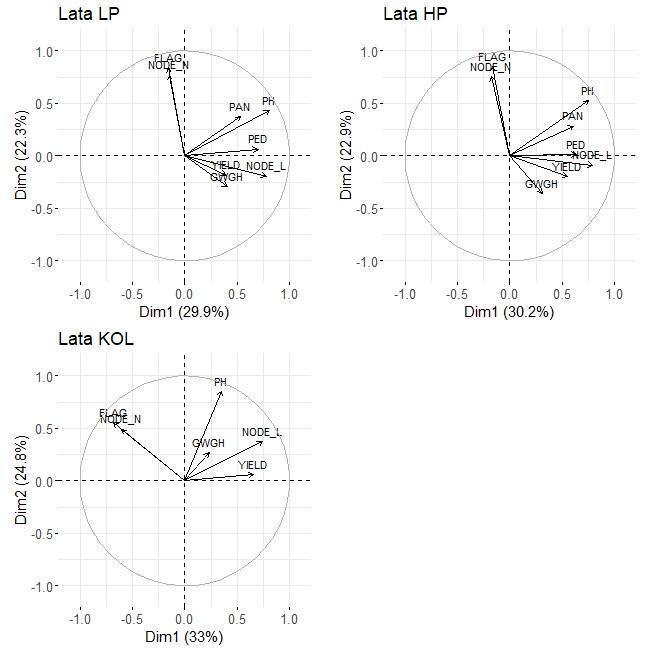


Principal component analysis plots of the adjusted mean phenotypic traits (BLUEs) in the three environments where Lata3 population was phenotyped (LP: low P, HP: high P, KOL: Kolombada station). FLAG: flag leaf appearance, PH: plant height, NODE_N: number of nodes, NODE_L: average length of the internode, PED: peduncle length, PAN: panicle length, GWGH: 1000 grain weight, YIELD: grain yield

**Figure S7:** Pearson correlation matrix plots of the phenotypic traits for each population and year of phenotyping combination

***Grinkan 2012***


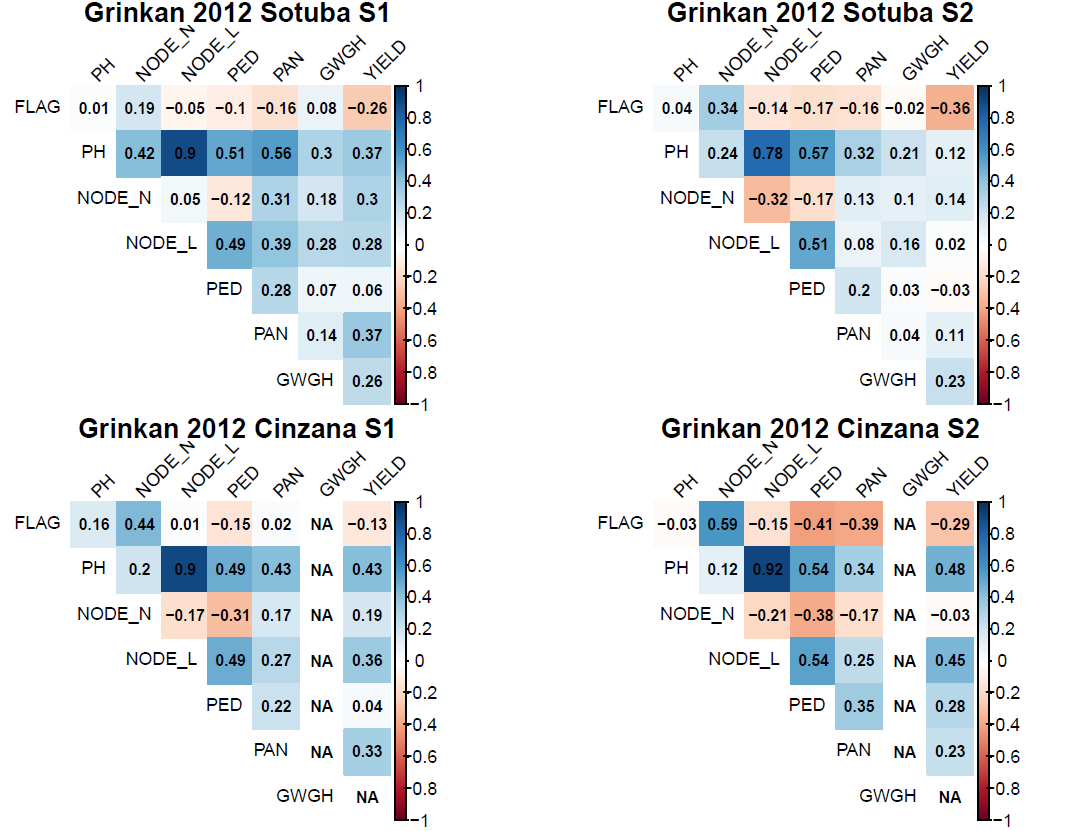


***Grinkan 2013***


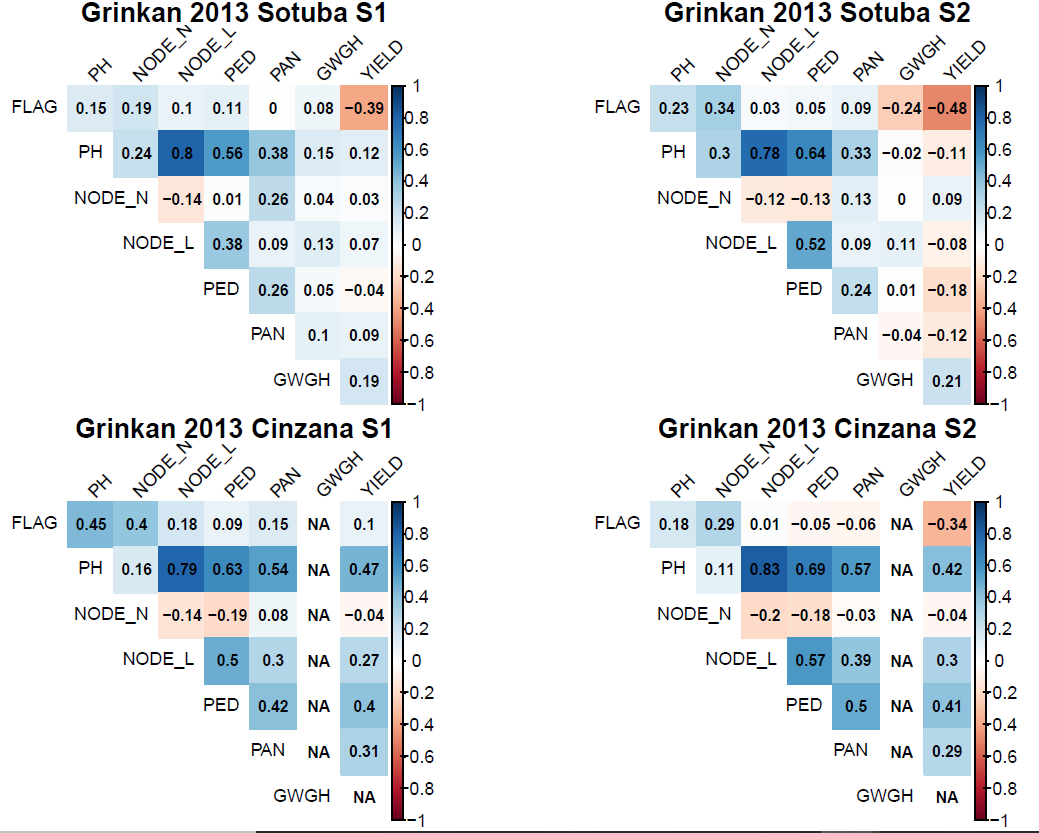


***Kenin-Keni 2012***


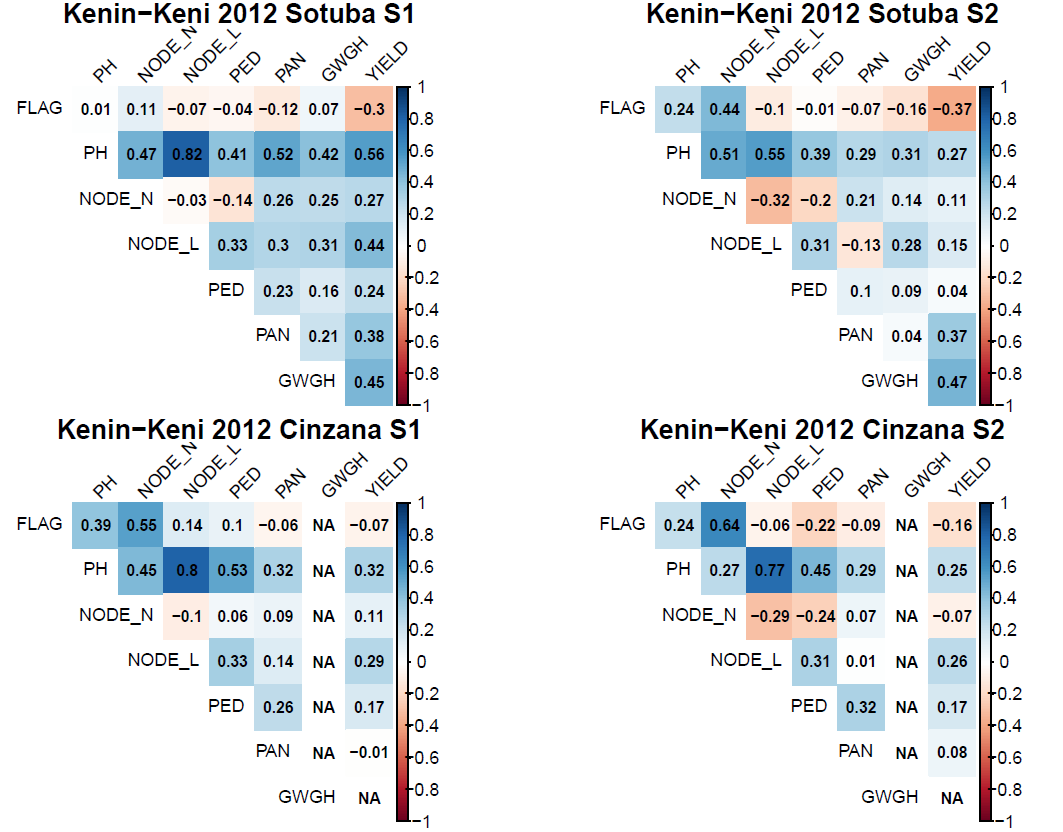


***Kenin-Keni 2013***


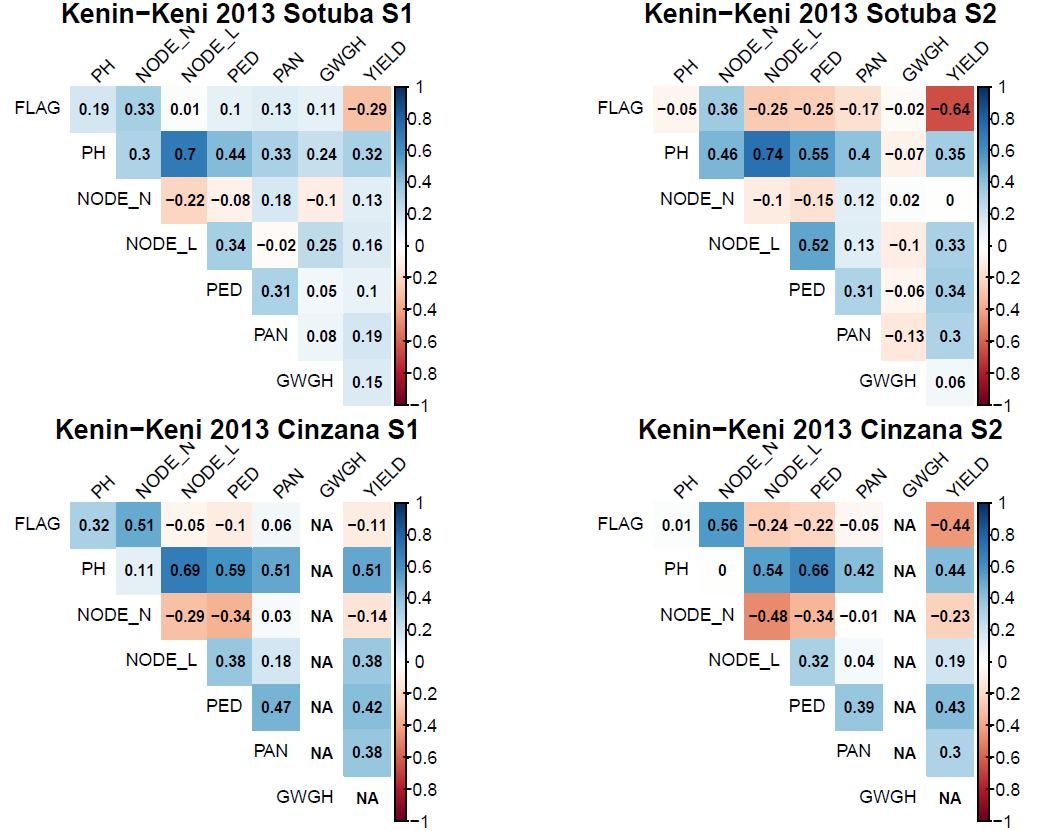


***Lata3 2013***


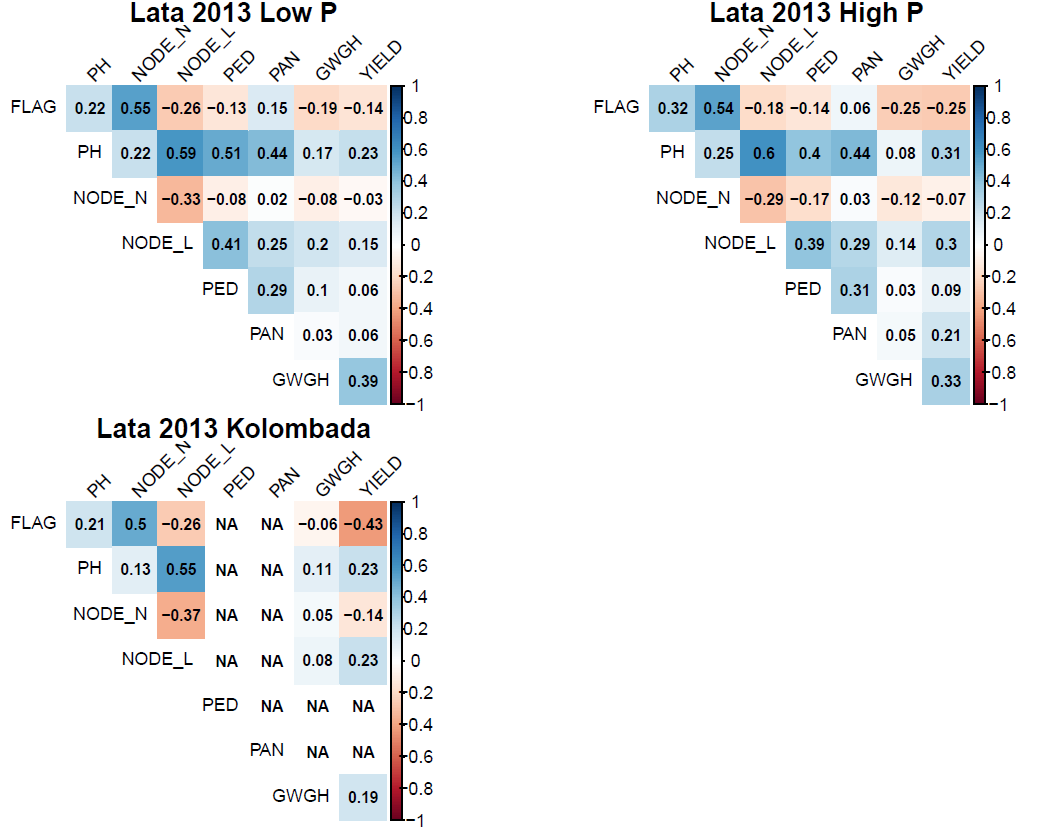


**Figure S8:**  Phenotype by environmental covariables analysis visualisation

***Flag leaf appearance (FLAG)***


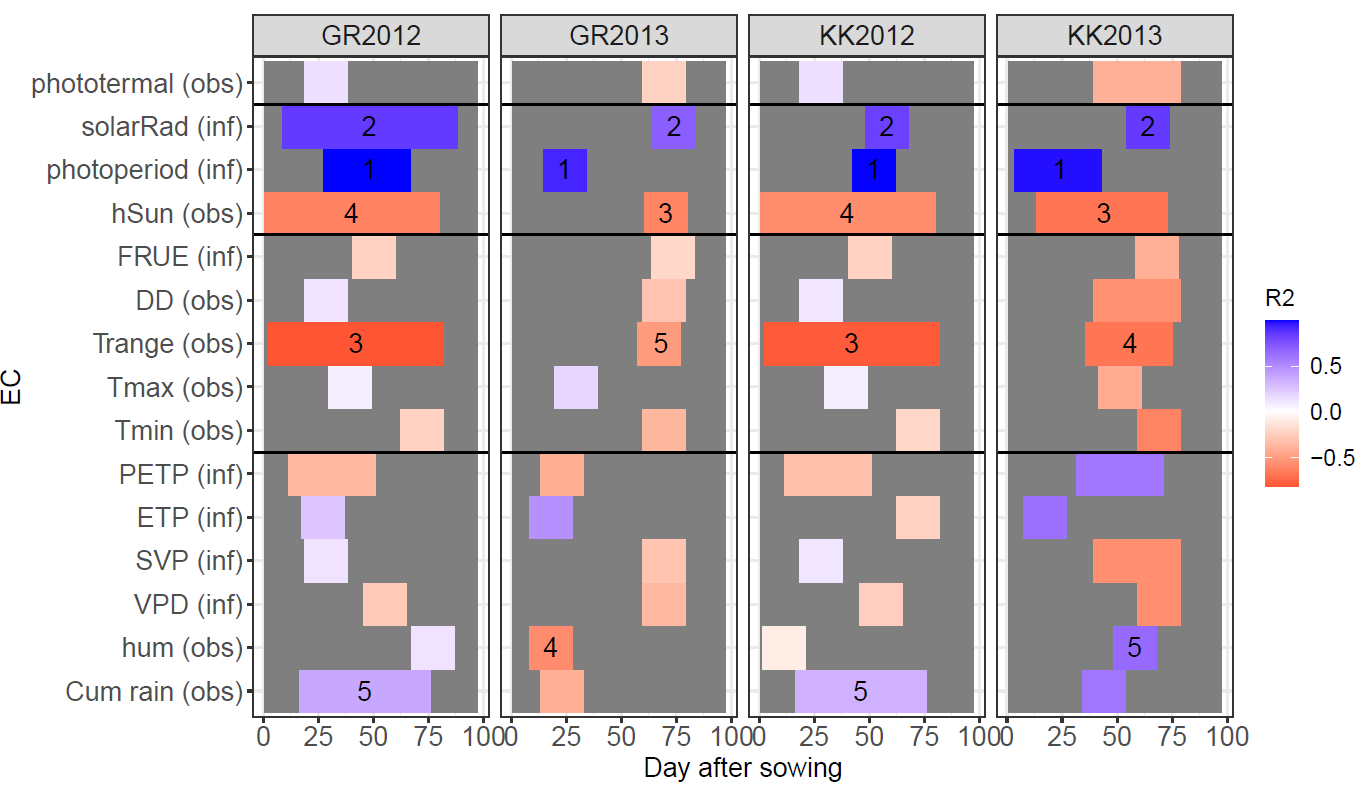


Effect of the environmental covariables (EC) on flag leaf appearance for the Grinkan and Kenin-Keni populations measured in environments Sotuba sowing 1-2 and Cinzana sowing 1-2 over the 2012 and 2013 seasons. The ECs and time window were selected using method S1. The intensity of the colour is proportional to the r squared (R^2^) between the within environment adjusted means and the environmental covariable (EC) value with direction of the effect (blue positive, red negative) and time window during which the effect is the strongest. The five most influential ECs are indicated with numbers (1 corresponding to the most influential one)

***Plant height***


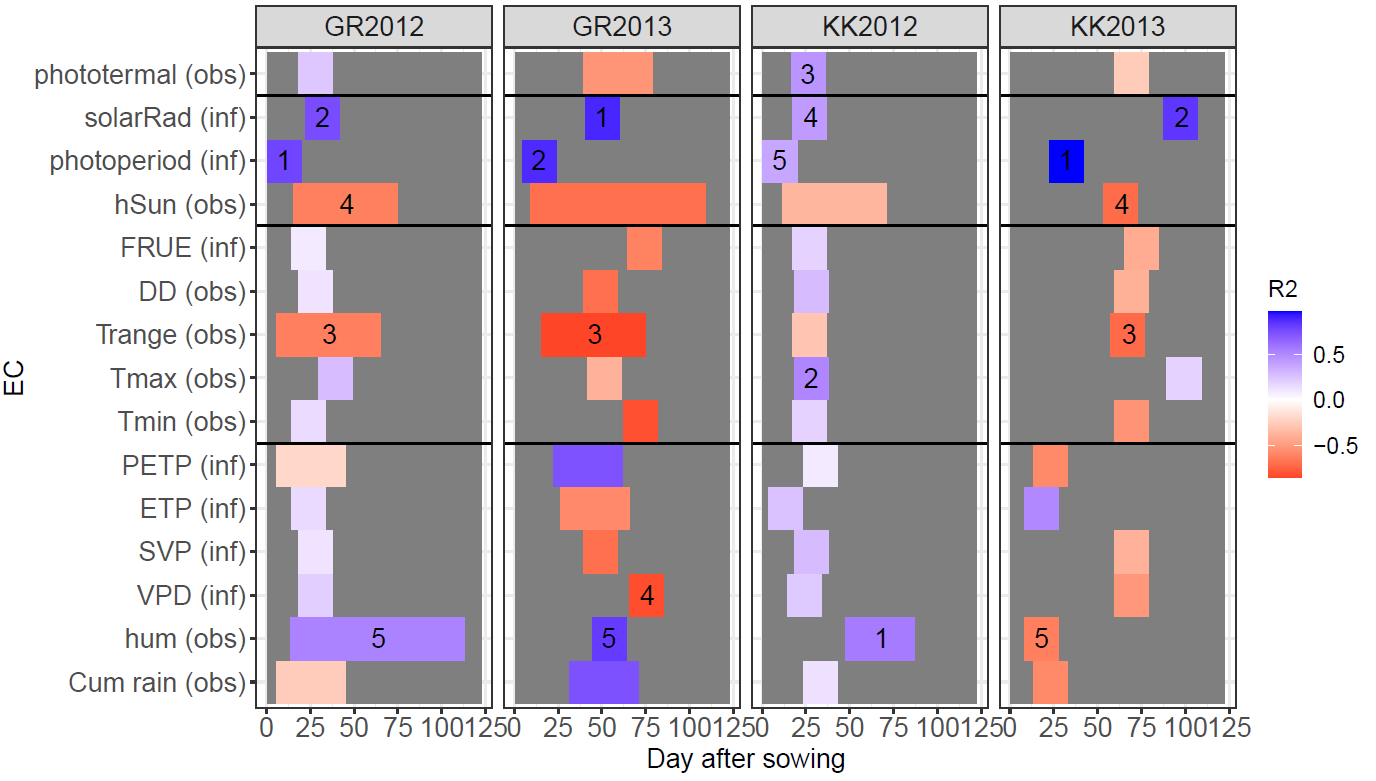


Effect of the environmental covariables (EC) on plant height for the Grinkan and Kenin-Keni populations measured in environments Sotuba sowing 1-2 and Cinzana sowing 1-2 over the 2012 and 2013 seasons. The ECs and time window were selected using method S1. The intensity of the colour is proportional to the r squared (R^2^) between the within environment adjusted means and the environmental covariable (EC) value with direction of the effect (blue positive, red negative) and time window during which the effect is the strongest. The five most influential ECs are indicated with numbers (1 corresponding to the most influential one)

***Number of internodes***


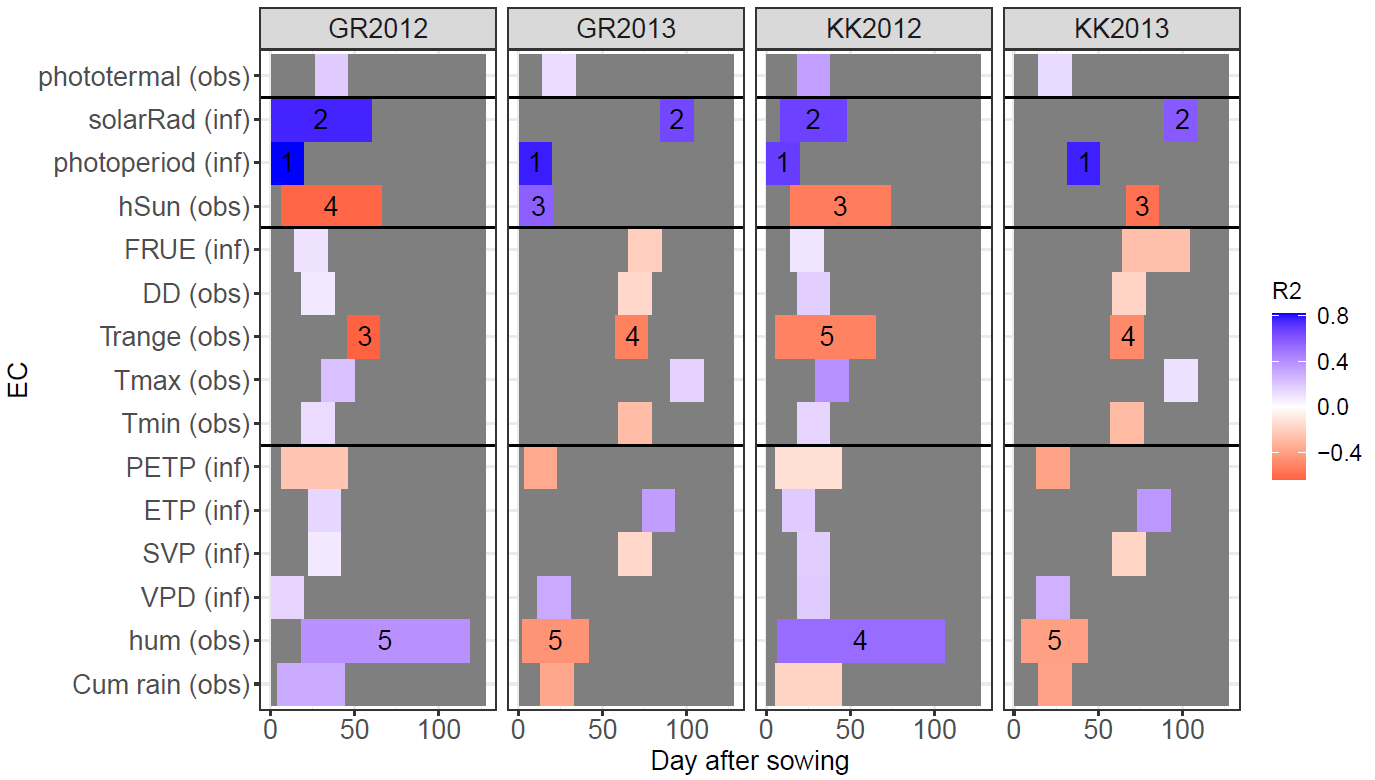


Effect of the environmental covariables (EC) on the number of internodes for the Grinkan and Kenin-Keni populations measured in environments Sotuba sowing 1-2 and Cinzana sowing 1-2 the 2012 and 2013 seasons. The ECs and time window were selected using method S1. The intensity of the colour is proportional to the r squared (R^2^) between the within environment adjusted means and the environmental covariable (EC) value with direction of the effect (blue positive, red negative) and time window during which the effect is the strongest. The five most influential ECs are indicated with numbers (1 corresponding to the most influential one)

***Average internode length***


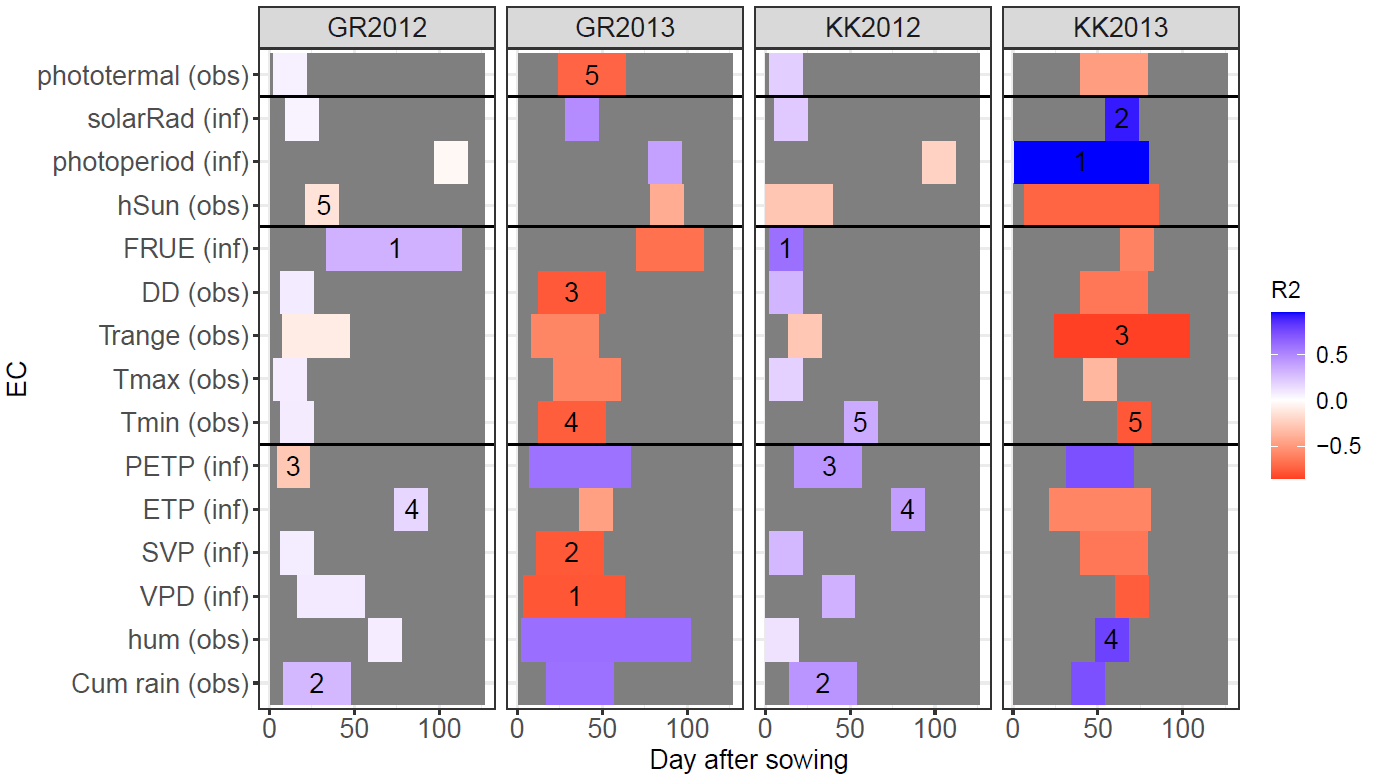


Effect of the environmental covariables (EC) on the average internode length for the Grinkan and Kenin-Keni populations measured in environments Sotuba sowing 1-2 and Cinzana sowing 1-2 over the 2012 and 2013 seasons. The ECs and time window were selected using method S1 The intensity of the colour is proportional to the r squared (R^2^) between the within environment adjusted means and the environmental covariable (EC) value with direction of the effect (blue positive, red negative) and time window during which the effect is the strongest. The five most influential ECs are indicated with numbers (1 corresponding to the most influential one)

***Peduncle length***


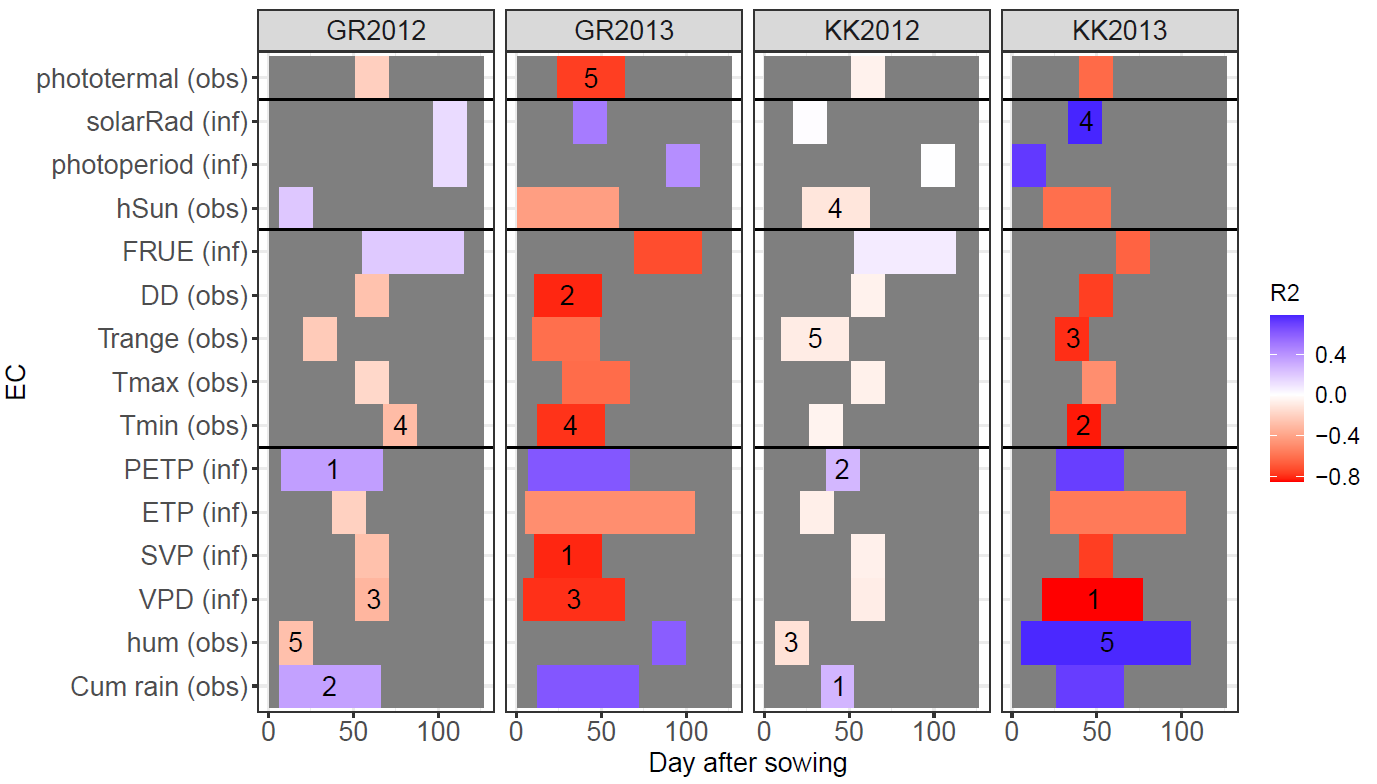


Effect of the environmental covariables (EC) on the peduncle length for the Grinkan and Kenin-Keni populations measured in environments Sotuba sowing 1-2 and Cinzana sowing 1-2 over the 2012 and 2013 seasons. The ECs and time window were selected using method S1. The intensity of the colour is proportional to the r squared (R^2^) between the within environment adjusted means and the environmental covariable (EC) value with direction of the effect (blue positive, red negative) and time window during which the effect is the strongest. The five most influential ECs are indicated with numbers (1 corresponding to the most influential one)

***Panicle length***


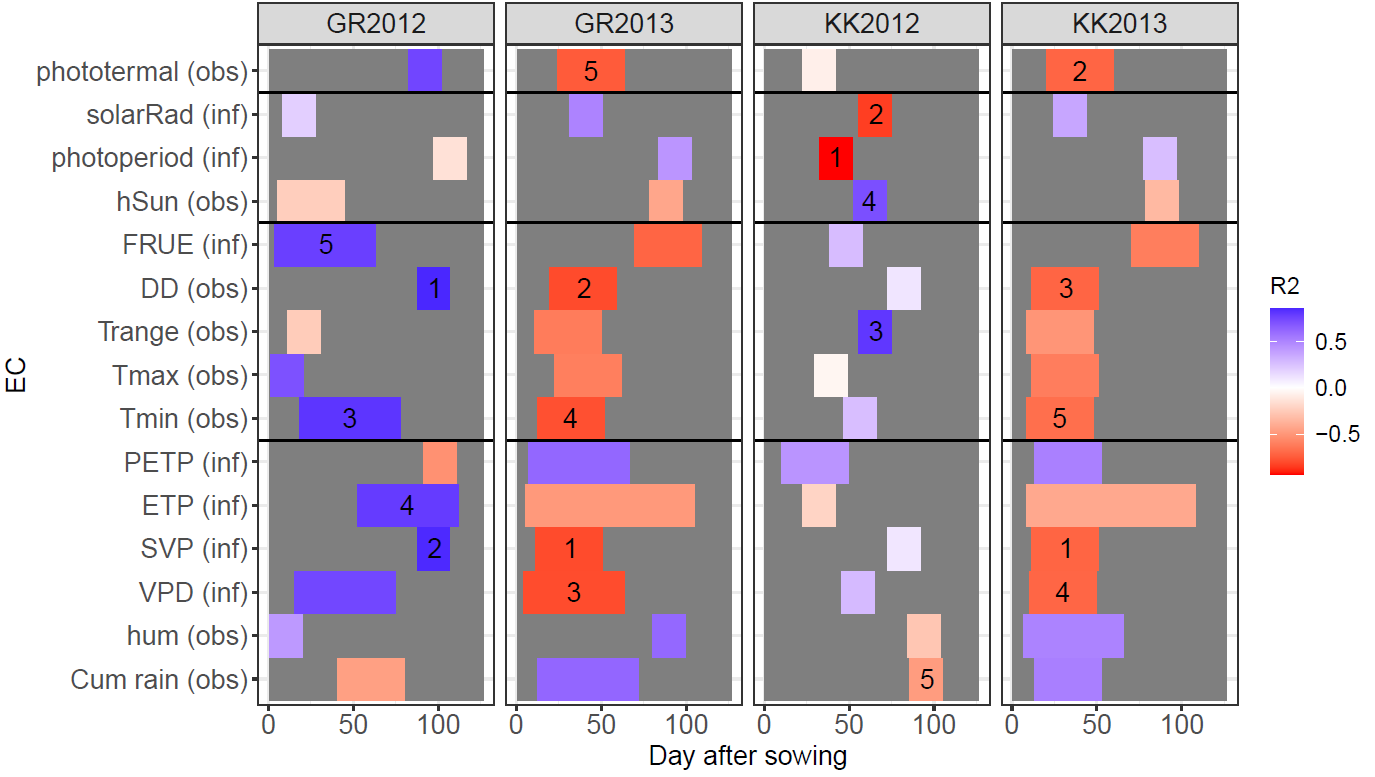


Effect of the environmental covariables (EC) on the panicle length for the Grinkan and Kenin-Keni populations measured in environments Sotuba sowing 1-2 and Cinzana sowing 1-2 over the 2012 and 2013 seasons. The ECs and time window were selected using method S1. The intensity of the colour is proportional to the r squared (R^2^) between the within environment adjusted means and the environmental covariable (EC) value with direction of the effect (blue positive, red negative) and time window during which the effect is the strongest. The five most influential ECs are indicated with numbers (1 corresponding to the most influential one)

***Grain yield (YIELD)***


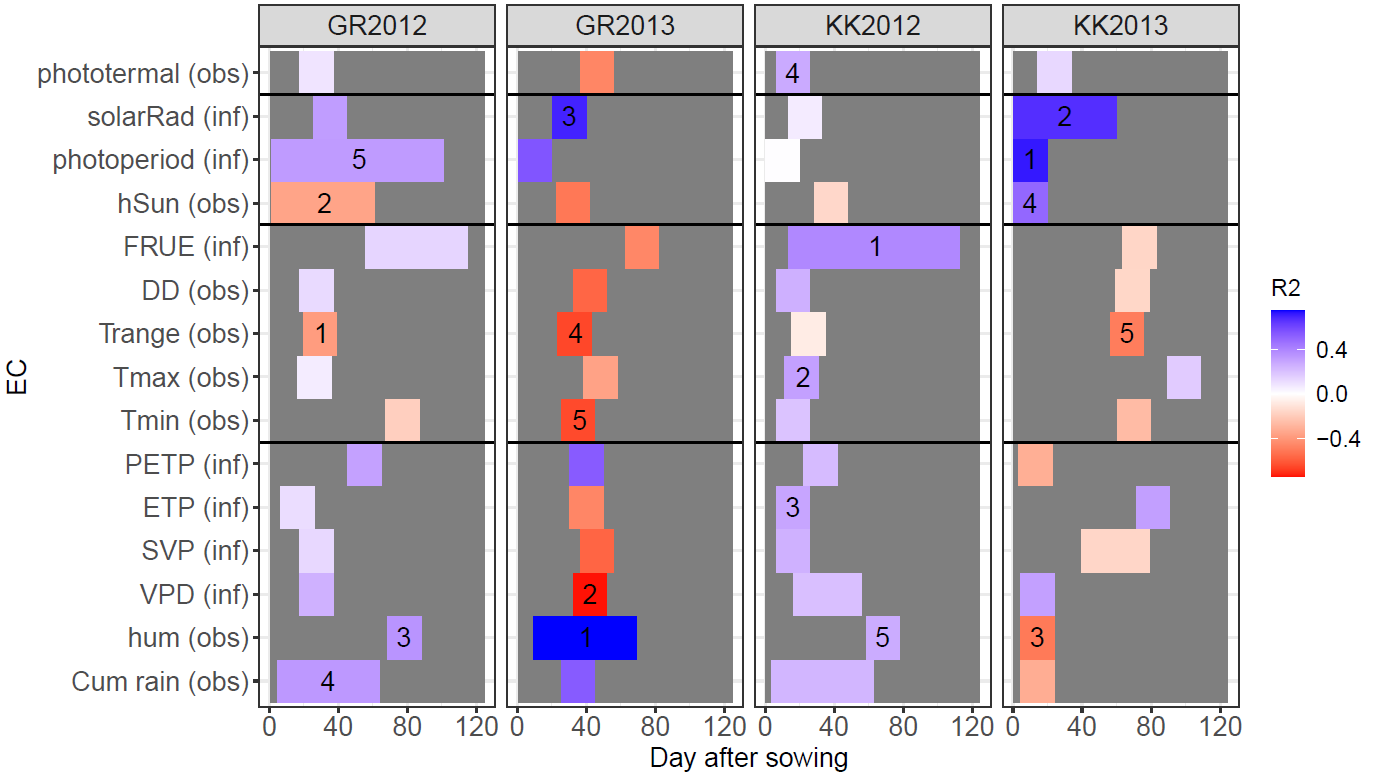


Effect of the environmental covariables (EC) on grain yield for the Grinkan and Kenin-Keni populations measured in environments Sotuba sowing 1-2 and Cinzana sowing 1-2 over the 2012 and 2013 seasons. The ECs and time window were selected using method S1. The intensity of the colour is proportional to the r squared (R^2^) between the within environment adjusted means and the environmental covariable (EC) value with direction of the effect (blue positive, red negative) and time window during which the effect is the strongest. The five most influential ECs are indicated with numbers (1 corresponding to the most influential one)

**Figure S9**: Effect plot of the detected QTLs over traits, sub-populations and environments

***Flag leaf appearance (FLAG)***


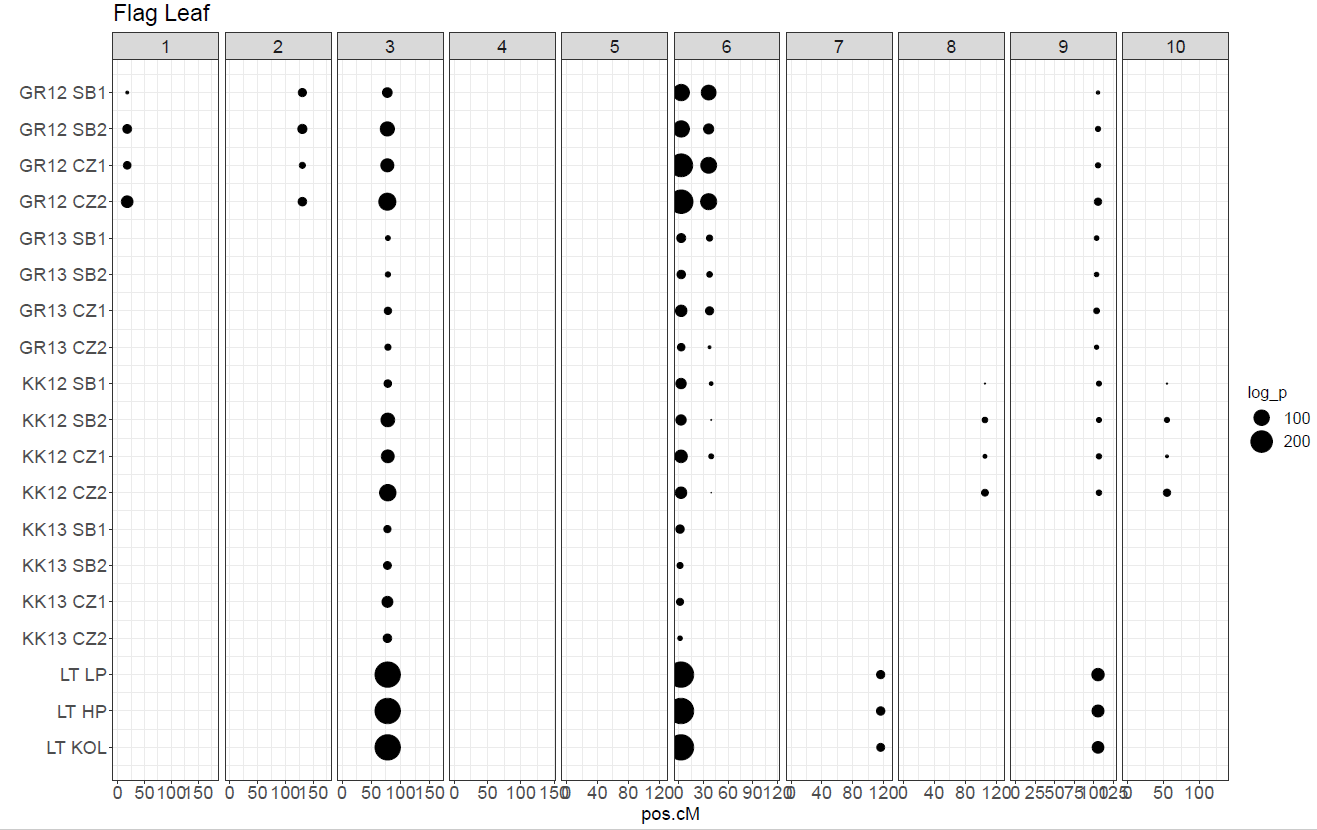


Significance of the QTL effect on flag leaf appearance given the different populations: Grinkan 2012 (GR12), Grinkan 2013 (GR13), Kenin-Keni 2012 (KK12), Kenin-Keni 2013 (KK13), and Lata within the tested environments: Sotuba sowing 1 and 2 (SB1, SB2), Cinzana sowing 1 and 2 (CZ1, CZ2), low/high phosphorus (LP, HP), and Kolombada. The size of the dot is proportional to the within environment significance

***Plant height***


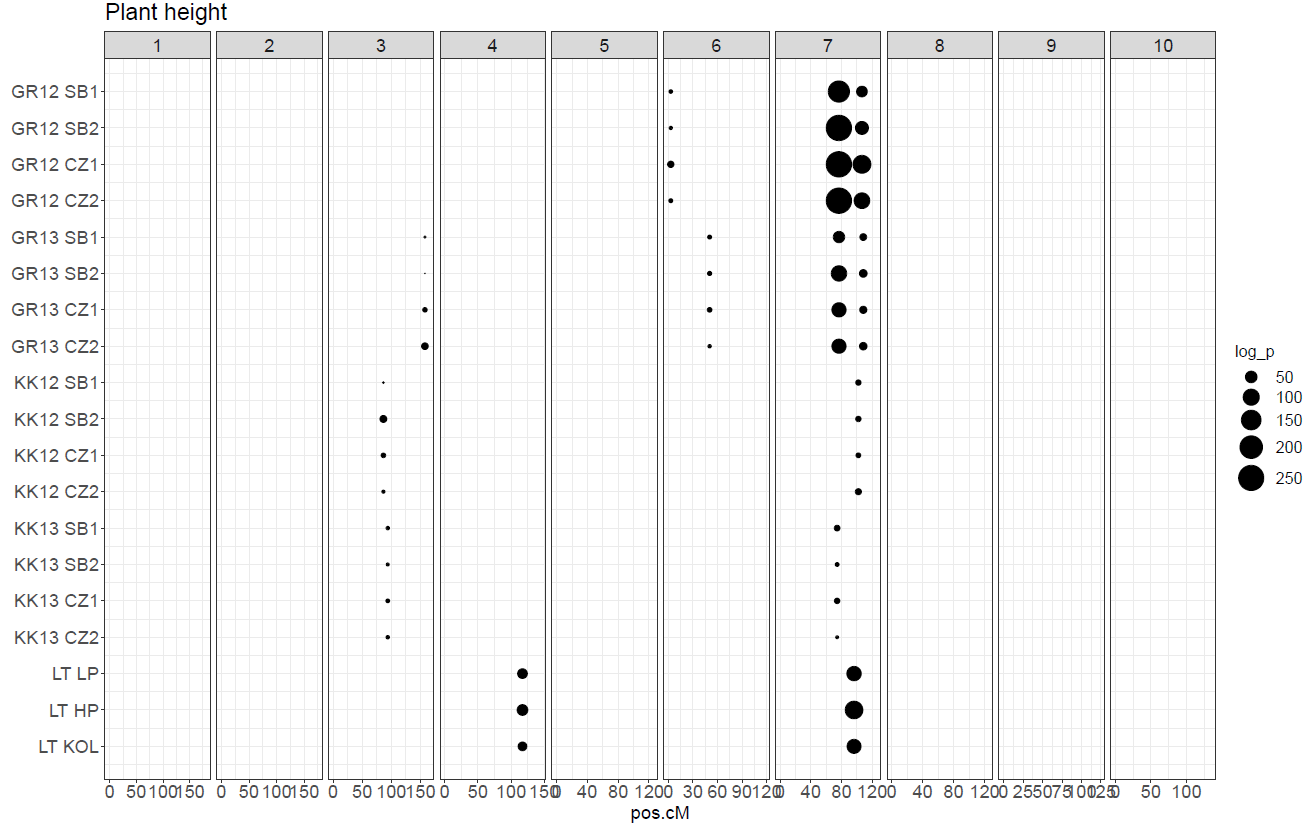


Significance of the QTL effect on flag leaf appearance given the different populations: Grinkan 2012 (GR12), Grinkan 2013 (GR13), Kenin-Keni 2012 (KK12), Kenin-Keni 2013 (KK13), and Lata within the tested environments: Sotuba sowing 1 and 2 (SB1, SB2) Cinzana sowing 1 and 2 (CZ1, CZ2), low/high phosphorus (LP, HP), and Kolombada. The size of the dot is proportional to the within environment significance

***Number of internodes (NODE_N)***


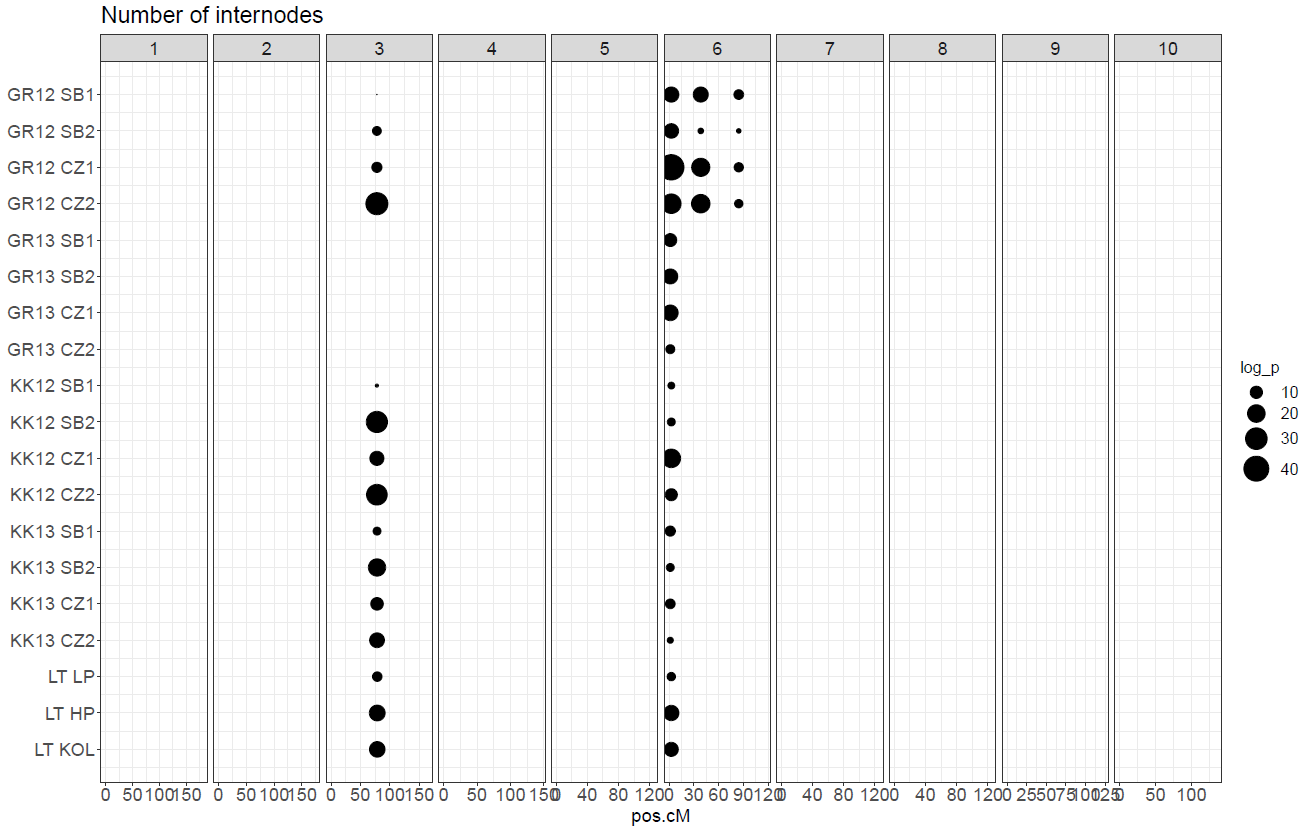


Significance of the QTL effect on the number of internodes given the different populations: Grinkan 2012 (GR12), Grinkan 2013 (GR13), Kenin-Keni 2012 (KK12), Kenin-Keni 2013 (KK13), and Lata within the tested environments: Sotuba sowing 1 and 2 (SB1, SB2), Cinzana sowing 1 and 2 (CZ1, CZ2), low/high phosphorus (LP, HP), and Kolombada. The size of the dot is proportional to the within environment significance

***Average internode length (NODE_L)***


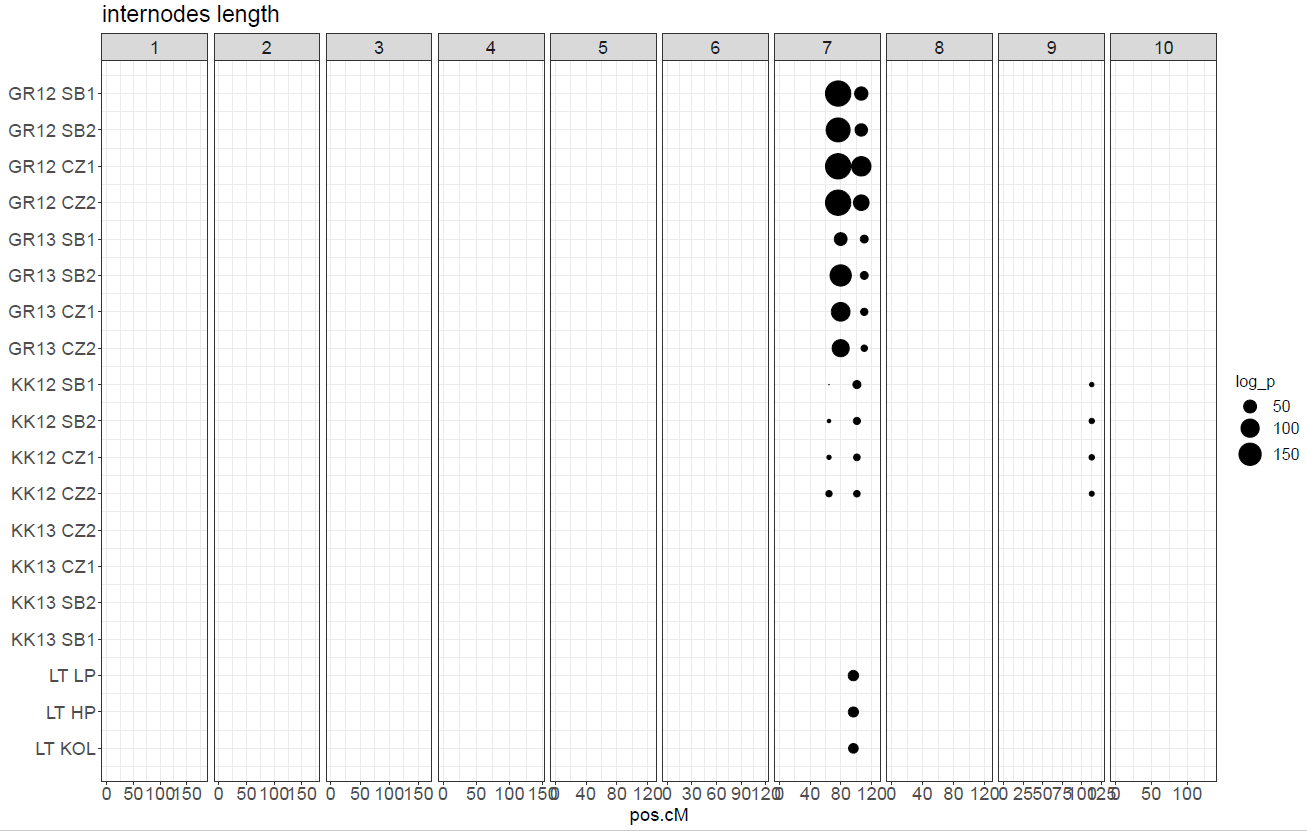


Significance of the QTL effect on the average internode length given the different populations: Grinkan 2012 (GR12), Grinkan 2013 (GR13), Kenin-Keni 2012 (KK12), Kenin-Keni 2013 (KK13), and Lata within the tested environments: Sotuba sowing 1 and 2 (SB1, SB2), Cinzana sowing 1 and 2 (CZ1, CZ2), low/high phosphorus (LP, HP), and Kolombada. The size of the dot is proportional to the within environment significance

***Peduncle length (PED)***


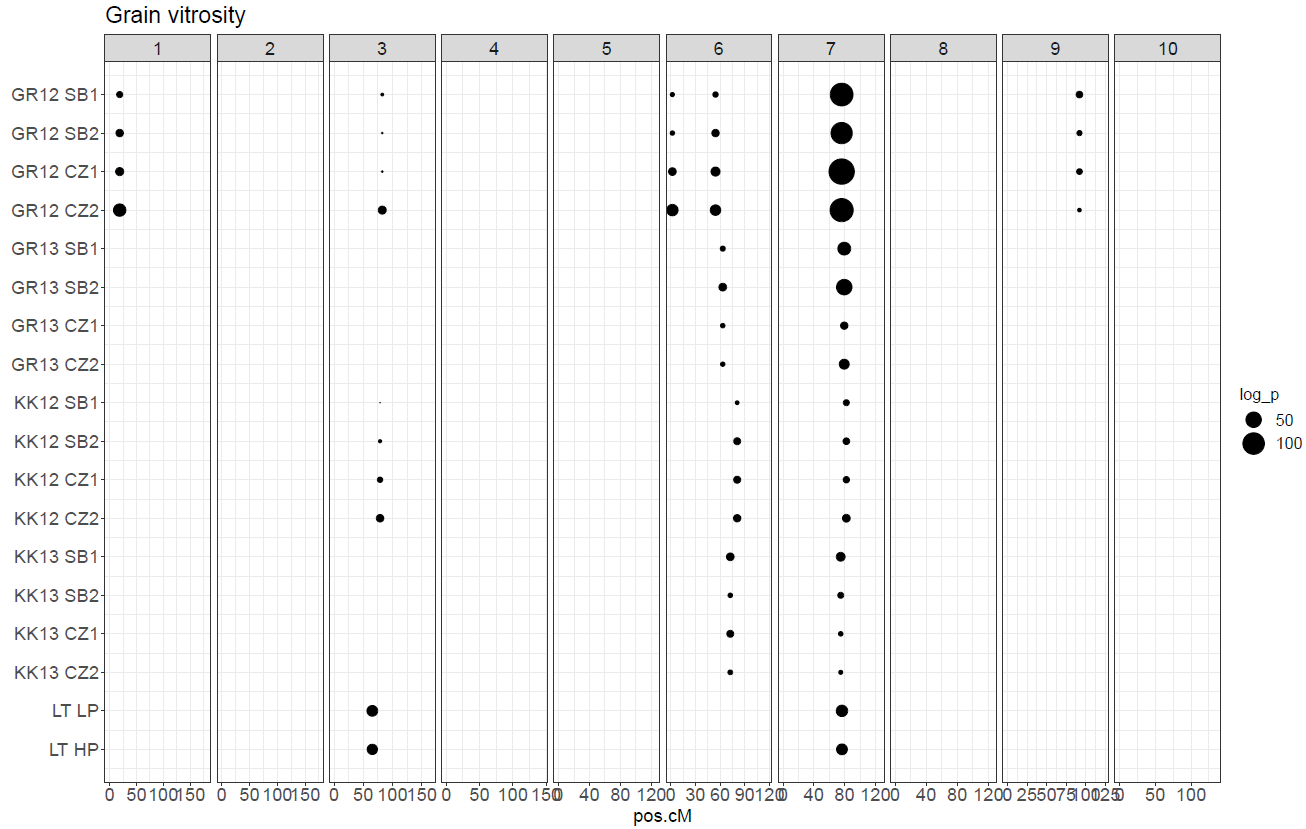


Significance of the QTL effect on the peduncle length given the different populations: Grinkan 2012 (GR12), Grinkan 2013 (GR13), Kenin-Keni 2012 (KK12), Kenin-Keni 2013 (KK13), and Lata within the tested environments: Sotuba sowing 1 and 2 (SB1, SB2), Cinzana sowing 1 and 2 (CZ1, CZ2), low/high phosphorus (LP, HP), and Kolombada. The size of the dot is proportional to the within environment significance

***Panicle length (PAN)***


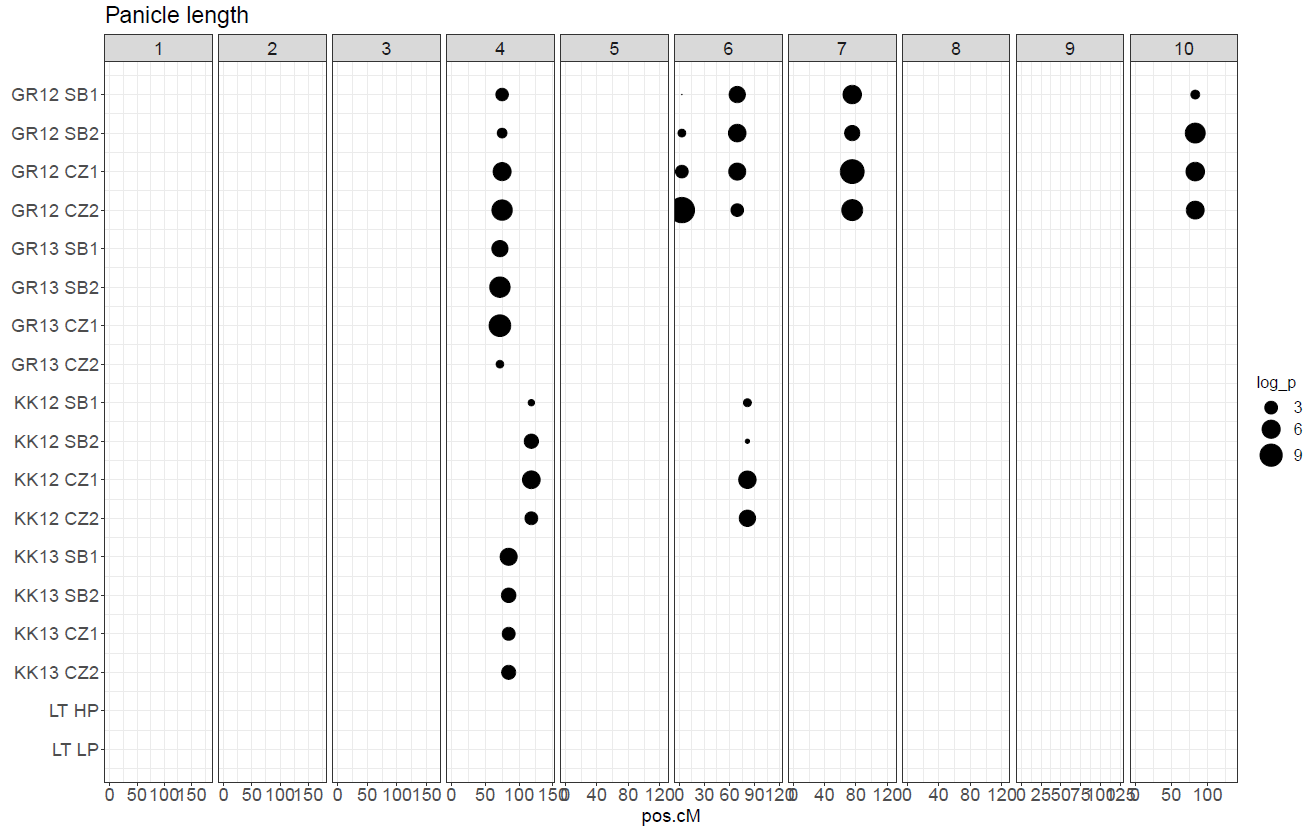


Significance of the QTL effect on the panicle length given the different populations: Grinkan 2012 (GR12), Grinkan 2013 (GR13), Kenin-Keni 2012 (KK12), Kenin-Keni 2013 (KK13), and Lata within the tested environments: Sotuba sowing 1 and 2 (SB1, SB2), Cinzana sowing 1 and 2 (CZ1, CZ2), low/high phosphorus (LP, HP), and Kolombada. The size of the dot is proportional to the within environment significance

***1000 grain weight (GWGH)***


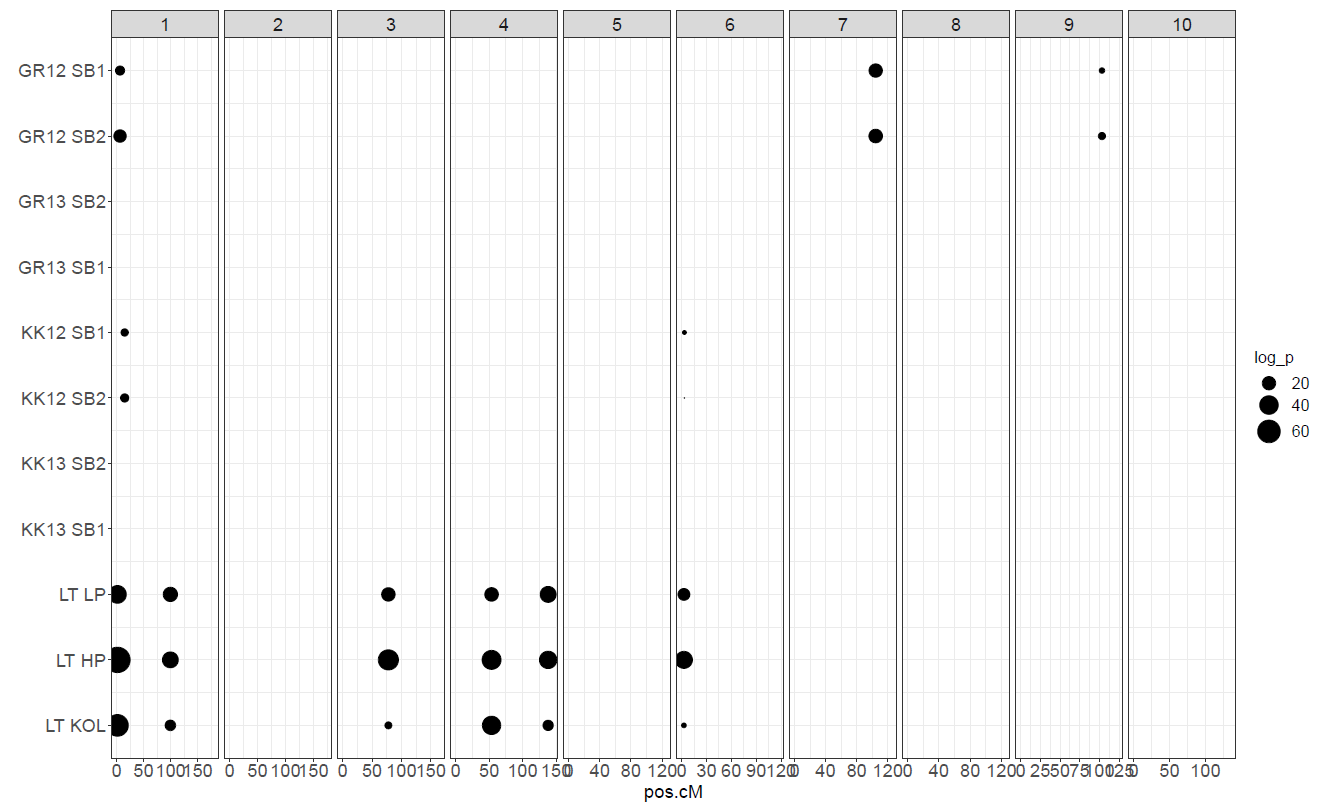


Significance of the QTL effect on 1000 grain weight given the different populations: Grinkan 2012 (GR12), Grinkan 2013 (GR13), Kenin-Keni 2012 (KK12), Kenin-Keni 2013 (KK13), and Lata within the tested environments: Sotuba sowing 1 and 2 (SB1, SB2), Cinzana sowing 1 and 2 (CZ1, CZ2), low/high phosphorus (LP, HP), and Kolombada. The size of the dot is proportional to the within environment significance

***Grain yield (YIELD)***


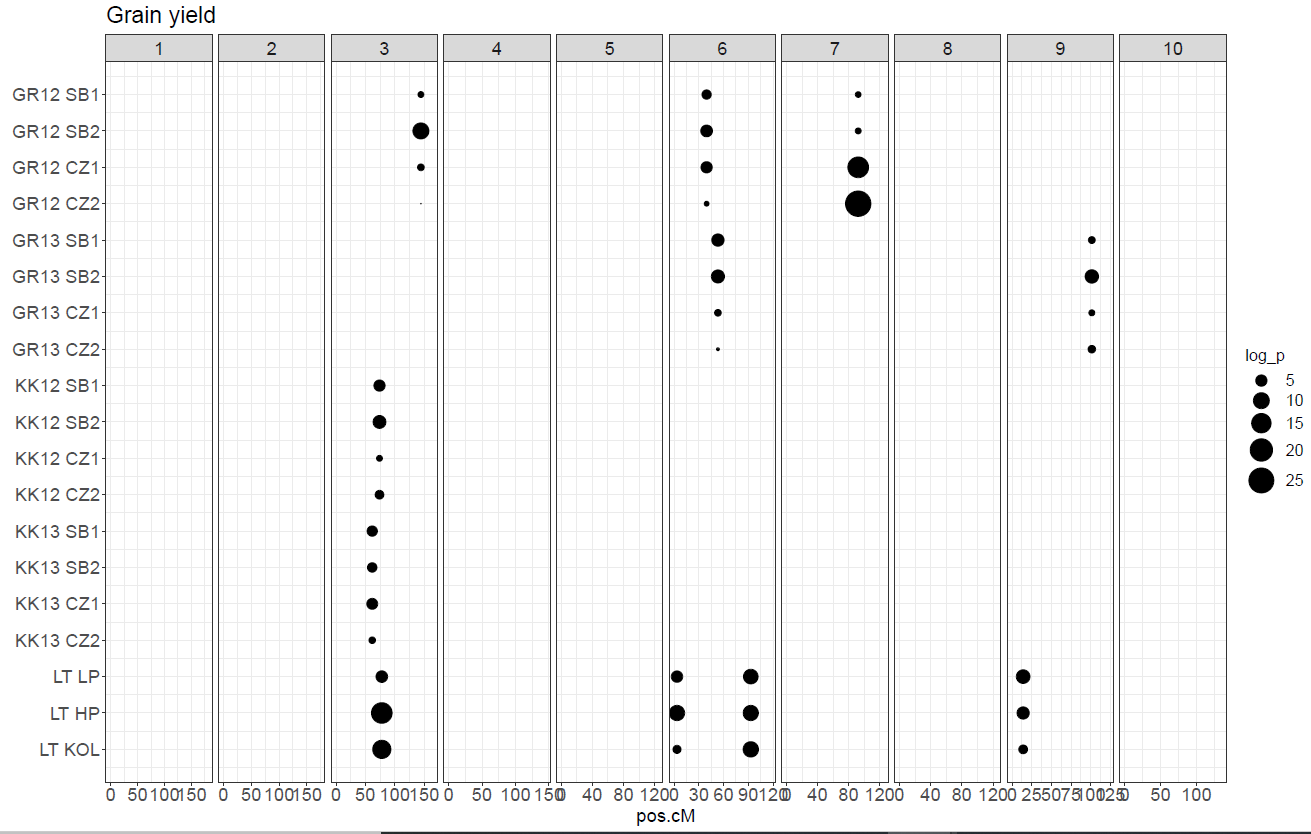


Significance of the QTL effect on grain yield given the different populations: Grinkan 2012 (GR12), Grinkan 2013 (GR13), Kenin-Keni 2012 (KK12), Kenin-Keni 2013 (KK13), and Lata within the tested environments: Sotuba sowing 1 and 2 (SB1, SB2), Cinzana sowing 1 and 2 (CZ1, CZ2), low/high phosphorus (LP, HP), and Kolombada. The size of the dot is proportional to the within environment significance

**Supplemental tables**

**Table S1:** Synonyms of the parental lines’ names

| **This Study** | **germplasm_Name** | **Variety name** | **Alternative name** | **BCNAM_PROJECT_ICRISAT_CODE** | **BCNAM_PROJECT_IER_CODE** | **ICRISAT accession no** | **USDA accession no** |
| --- | --- | --- | --- | --- | --- | --- | --- |
| Grinkan | Grinkan |  | 02-SB-F4DT-275 | 1085 | V12 |  |  |
| Kenin-Keni | Keninkeni |  | V248/08 |  | V13 |  |  |
| Lata3 | Lata3 | Lata 3 | GPN01 S01 267-9-3-3-vr | 1097 | V14 |  |  |
| Fara-Fara | IS24887 | Fara Fara |  | 1096 | V20 | IS24887 |  |
| E36-1 | E36-1 |  |  |  | V15 | IS30469 |  |
| IS15401 | IS15401 | Soumalemba |  | 1086 | V17 | IS15401 |  |
| IS23540 | IS23540 | Ganga |  | 1087 | V18 | IS23540 |  |
| B35 | B35 |  |  |  | V33 |  |  |
| Konotene | Konotene |  |  |  | V5 | IS25705 |  |
| SC566-14 | SC566-14 |  |  | 1089 | V19 |  | PI533871 |
| Framida | Framida |  |  | 1084 | V16 |  |  |
| CSM417 | CSM417 | Tiemantieteli |  |  | V11 |  |  |
| CSM63 | CSM63E | Jakumbe |  |  | V2 |  |  |
| CSM388 | CSM388 | Jigi Seme |  |  | V3 |  |  |
| Gadiaba Dié | Gadiaba Dié |  |  |  | V4 | IS25916 | PI525840,PI585749 |
| W. Kaura | White Kaura |  | SSV 20043 |  | V25 |  |  |
| V33/08 | V33/08 |  | G03-1-118 |  | V7 |  |  |
| Kalaban | Kalaban |  | 00KOF5DT19 |  | V10 |  |  |
| Malisor 84-7 | Malisor84-7 | Dabitinnen |  |  | V9 |  |  |
| BimbG | BimbG | Bimbiri Soumalen | BB_G_5_34 |  | V35 |  |  |
| Hafijeka | IS23645 | Hafijega |  | 1088 | V21 | IS23645 |  |
| S. Kaura | Short Kaura |  | SK-5912 | 1090 | V22 | IS10699 |  |
| Sangatigui | Sanga Tigi |  | 98-BE-F5P-84 |  | V8 |  |  |
| DouaG | Doua-G |  |  | 1092 | V31 |  |  |
| Gnossiconi | Gnossiconi |  |  | 1093 | V23 |  |  |
| Ngolofing | CSM660 | Ngolofing |  | 1091 | V29 |  |  |
| Sambalma | Sambalma (4) | Sambalma |  | 1095 | V26 |  |  |

**Table S2**: Consensus map statistics

| **Chr** | **N. markers^1^** | **Length [cM]** | **N. CO^2^ Grinkan** | **N. CO^2^ Kenin-Keni** | **N. CO^2^ Lata3** |
| --- | --- | --- | --- | --- | --- |
| 1 | 8154 | 179.8 | 6562 | 2591 | 2570 |
| 2 | 6717 | 173.8 | 5470 | 2425 | 2158 |
| 3 | 7258 | 164.8 | 5571 | 2450 | 2534 |
| 4 | 5593 | 144.9 | 4887 | 2035 | 2183 |
| 5 | 3940 | 124.6 | 4577 | 2327 | 1960 |
| 6 | 4380 | 117.2 | 4548 | 2076 | 1908 |
| 7 | 3674 | 124.9 | 3894 | 1474 | 1574 |
| 8 | 3533 | 124 | 3895 | 1648 | 1733 |
| 9 | 4463 | 123.1 | 3967 | 1693 | 1784 |
| 10 | 3833 | 134.9 | 4298 | 1624 | 1716 |
| Total | 51545 | 1411.9 | 47669 | 20343 | 20120 |

1. Number of polymorphic markers in the consensus map
2. N. CO: total number of crossing over estimated in the different single reference BCNAM populations (Grinkan, Kenin-Keni, Lata3)

**Table S3**: Sowing dates of the field trials

|  | 2012 | | 2013 | |
| --- | --- | --- | --- | --- |
|  | Sowing 1 | Sowing 2 | Sowing 1 | Sowing 2 |
| Sotuba | 26-Jun | 24-Jul | 29-Jun | 21-Jul |
| Cinzana | 29-Jun | 21-Jul | 21-Jul | 07-Aug |
| Samanko |  |  | 28-Jun |  |
| Kolombada |  |  | 28-Jun |  |

**Table S4**: Environmental description of the multi-location trials

| **Location** | **lat** | **lon** | **year** | **sowing** | **cum rain**  **[mm]** | **av. Humidity**  **[%]** | **av. temp. [d]** | **av. maxT [d]** | **av. minT**  **[d]** | **av. photop. [h]** |
| --- | --- | --- | --- | --- | --- | --- | --- | --- | --- | --- |
| Sotuba | 12.65 | -7.93 | 2012 | Sw1 | 851.5 | 77.1 | 26.6 | 31.3 | 21.9 | 12.3 |
|  |  |  |  | Sw2 | 570.3 | 74.3 | 27 | 32.3 | 21.7 | 12.0 |
|  |  |  | 2013 | Sw1 | 799.8 | 77.7 | 27.2 | 31.8 | 22.6 | 12.3 |
|  |  |  |  | Sw2 | 698.2 | 75.7 | 27.1 | 32.4 | 21.9 | 12.0 |
| Cinzana | 13.25 | -5.96 | 2012 | Sw1 | 707.9 | 79.3 | 28.1 | 32.9 | 23.3 | 12.3 |
|  |  |  |  | Sw2 | 529.3 | 75.5 | 28.7 | 34.1 | 23.3 | 12.0 |
|  |  |  | 2013 | Sw1 | 377.1 | 70.3 | 28.7 | 34.2 | 23.3 | 12.0 |
|  |  |  |  | Sw2 | 338.5 | 65.9 | 28.7 | 34.6 | 22.7 | 11.9 |
| Samanko | 12.53 | -8.07 | 2013 | LP | 967 | 79.8* | 27.5 | 33.3 | 21.7 | 12.3 |
|  |  |  |  | HP | 967 | 79.8* | 27.5 | 33.3 | 21.7 | 12.3 |
| Kolombada | 12.69 | -7.01 | 2013 | std | 439.7 | 77.6* | 26.7* | 31.2* | 22.3* | 12.3 |

*: Synthetic data from Nasapower (Spark 2018)

**Table S5:** Detail of the phenotyping per sub-population, year, environment, and trait

| Population | Year | Env | FLAG | PH | NODE_N | NODE_L | PED | PAN | GWGH | YIELD |
| --- | --- | --- | --- | --- | --- | --- | --- | --- | --- | --- |
| Grinkan | 2012 | Sotuba Sowing 1 | X | X | X | X | X | X | X | X |
|  |  | Sotuba Sowing 2 | X | X | X | X | X | X | X | X |
|  |  | Cinzana Sowing 1 | X | X | X | X | X | X |  | X |
|  |  | Cinzana Sowing 2 | X | X | X | X | X | X |  | X |
|  | 2013 | Sotuba Sowing 1 | X | X | X | X | X | X | X | X |
|  |  | Sotuba Sowing 2 | X | X | X | X | X | X | X | X |
|  |  | Cinzana Sowing 1 | X | X | X | X | X | X |  | X |
|  |  | Cinzana Sowing 2 | X | X | X | X | X | X |  | X |
| Kenin-Keni | 2012 | Sotuba Sowing 1 | X | X | X | X | X | X | X | X |
|  |  | Sotuba Sowing 2 | X | X | X | X | X | X | X | X |
|  |  | Cinzana Sowing 1 | X | X | X | X | X | X |  | X |
|  |  | Cinzana Sowing 2 | X | X | X | X | X | X |  | X |
|  | 2013 | Sotuba Sowing 1 | X | X | X | X | X | X | X | X |
|  |  | Sotuba Sowing 2 | X | X | X | X | X | X | X | X |
|  |  | Cinzana Sowing 1 | X | X | X | X | X | X |  | X |
|  |  | Cinzana Sowing 2 | X | X | X | X | X | X |  | X |
| Lata3 | 2013 | Samanko Low P | X | X | X | X | X | X | X | X |
|  |  | Samanko High P | X | X | X | X | X | X | X | X |
|  |  | Kolombada | X | X | X | X |  |  | X | X |

**Table S6:** List of environmental covariables available for the Sotuba and Cinzana locations

| Category | EC | Abbreviation | Unit | Observed/inferred | Sum/mean |
| --- | --- | --- | --- | --- | --- |
| Atmospheric | cumulated rain | cum rain | mm | obs | sum |
|  | humidity | hum | % | obs | mean |
|  | vapour pressure deficit | VPD | kPa | inf | mean |
|  | slope of saturation VP curve | SVP | kPa/d | inf | mean |
|  | potential evapotranspiration | ETP | mm/day | inf | mean |
|  | water deficit | PETP | mm/day | inf | mean |
| Temperature | minimum temperature | Tmin | d | obs | mean |
|  | maximum temperature | Tmax | d | obs | mean |
|  | temperature range | Trange | d | obs | mean |
|  | cumulated degree day | DD | dd | obs | sum |
|  | T effect on radiation use efficiency | FRUE | 0-1 | inf | mean |
| Radiation | cumulated hour of sun | hsun | h | obs | sum |
|  | photoperiod | photo | h | inf | mean |
|  | solar radiation | SolRad | MJ/m^2/day | inf | sum |
| Photothermal | Photothermal (photoperiod * DD) | photothermal | h*dd | obs | sum |

**Table S7:** QTL detection threshold

| **Population** | **-log10(p-val)** |
| --- | --- |
| GR2012 | 4.647689 |
| GR2013 | 4.483628 |
| KK2012 | 4.181491 |
| KK2013 | 4.187031 |
| Lata | 4.428637 |

**Table S8**: Percentage of SNPs with at least one parent homozygous for the minor allele in the parents from the sorghum WCA-BCNAM and the sorghum US NAM populations. The percentages were calculated for different thresholds of minor allele frequency (5, 2.5 and 1%) to retain a specific locus from the initial set of 2’877’109 SNPs.

| Minor allele frequency threshold [%] | WCA-BCNAM [%] | US NAM [%] |
| --- | --- | --- |
| 5 | 86.8 | 57.5 |
| 2.5 | 78.7 | 46.9 |
| 1 | 68.9 | 38.1 |

**Table S9**: Within cross variance components: genotypic variance ($\sigma_{g}^{2}$) genotype by environment variance ($\sigma_{ge}^{2}$), error variance ($\sigma_{e}^{2}$) and broad sense heritability (*h^2^*) of the different crosses. Empty cells correspond to traits that have not been evaluated in a specific cross/environment.

***Grinkan sub-population characterized in 2012***

|  |  | Flag leaf [d] | | | | Plant height [cm] | | | | Number of internodes | | | | Average internode length [cm] | | | |
| --- | --- | --- | --- | --- | --- | --- | --- | --- | --- | --- | --- | --- | --- | --- | --- | --- | --- |
| ***Grinkan*** | N | Av | $\sigma_{g}^{2}$ | $\sigma_{ge}^{2}$ | $h^{2}$ | Av | $\sigma_{g}^{2}$ | $\sigma_{ge}^{2}$ | $h^{2}$ | Av | $\sigma_{g}^{2}$ | $\sigma_{ge}^{2}$ | $h^{2}$ | Av | $\sigma_{g}^{2}$ | $\sigma_{ge}^{2}$ | $h^{2}$ |
| Fara | 117 | 83.6 | 15.9 | 13.8 | 0.82 | 175.7 | 1360.1 | 662.6 | 0.89 | 14.3 | 1.2 | 5.3 | 0.48 | 7.6 | 3.4 | 2.1 | 0.86 |
| E36-1 | 125 | 76.3 | 35.0 | 14.7 | 0.91 | 166.6 | 738.3 | 645.0 | 0.82 | 13.1 | 2.1 | 4.6 | 0.65 | 8.0 | 2.2 | 2.7 | 0.77 |
| IS15401 | 113 | 80.4 | 8.0 | 7.8 | 0.8 | 166.8 | 615.9 | 624.3 | 0.8 | 14.3 | 0.8 | 5.5 | 0.37 | 7.3 | 1.9 | 2.1 | 0.78 |
| IS23540 | 61 | 77.8 | 15.9 | 9.4 | 0.87 | 176.3 | 1098.6 | 641.6 | 0.87 | 13.2 | 0.5 | 4.2 | 0.32 | 8.3 | 5.5 | 2.7 | 0.89 |
| B35 | 118 | 79.1 | 25.4 | 19.9 | 0.84 | 156.7 | 888.6 | 489.9 | 0.88 | 13.7 | 1.4 | 4.1 | 0.57 | 6.7 | 2.7 | 1.8 | 0.86 |
| Kono. | 134 | 78.9 | 6.8 | 6.2 | 0.81 | 180.7 | 1837.9 | 823.9 | 0.9 | 13.1 | 0.5 | 3.7 | 0.35 | 8.9 | 7.5 | 2.7 | 0.92 |
| SC566-14 | 142 | 74.5 | 53.5 | 23.9 | 0.9 | 173.2 | 1335.7 | 564.3 | 0.9 | 13.0 | 3.2 | 3.8 | 0.76 | 8.2 | 5.9 | 2.3 | 0.91 |
| Framida | 140 | 76.2 | 19.5 | 6.5 | 0.92 | 172.6 | 1077.2 | 445.4 | 0.91 | 13.0 | 1.9 | 3.2 | 0.69 | 8.3 | 4.4 | 1.9 | 0.90 |
| CSM417 | 160 | 75.9 | 16.8 | 5.8 | 0.92 | 179.1 | 1974.4 | 946.5 | 0.89 | 11.7 | 1.4 | 2.6 | 0.64 | 9.3 | 8.8 | 4.1 | 0.89 |
| CSM63 | 67 | 75.3 | 28.8 | 15.0 | 0.89 | 187.1 | 1697.7 | 1250.4 | 0.84 | 12.8 | 1.9 | 4.5 | 0.63 | 9.2 | 8.7 | 3.7 | 0.90 |
| CSM388 | 149 | 76.6 | 24.1 | 4.8 | 0.95 | 201.7 | 1779.5 | 1283.5 | 0.85 | 13.5 | 1.7 | 4.1 | 0.62 | 9.7 | 6.3 | 3.0 | 0.89 |
| Gadiaba | 135 | 80.1 | 9.5 | 4.6 | 0.89 | 192.6 | 2466.6 | 1211.0 | 0.89 | 14.3 | 1.5 | 4.1 | 0.58 | 8.9 | 9.0 | 3.4 | 0.91 |
| Wth Kaur | 137 | 79.1 | 12.4 | 6.2 | 0.89 | 171.8 | 1780.4 | 663.7 | 0.91 | 13.4 | 0.5 | 3.9 | 0.34 | 7.8 | 6.8 | 1.9 | 0.93 |
| Average | 123 | 78.0 |  |  | 0.88 | 177.0 |  |  | 0.87 | 13.3 |  |  | 0.5 | 8.3 |  |  | 0.9 |

|  |  | Peduncle length [cm] | | | | Panicle length [cm] | | | | Grain weight [g] | | | | Grain yield [ton/ha] | | | |
| --- | --- | --- | --- | --- | --- | --- | --- | --- | --- | --- | --- | --- | --- | --- | --- | --- | --- |
| ***Grinkan*** | N | Av | $\sigma_{g}^{2}$ | $\sigma_{ge}^{2}$ | $h^{2}$ | Av | $\sigma_{g}^{2}$ | $\sigma_{ge}^{2}$ | $h^{2}$ | Av | $\sigma_{g}^{2}$ | $\sigma_{ge}^{2}$ | $h^{2}$ | Av | $\sigma_{g}^{2}$ | $\sigma_{ge}^{2}$ | $h^{2}$ |
| Fara | 117 | 36.8 | 23.2 | 33.3 | 0.74 | 30.7 | 8.7 | 22.6 | 0.61 | 25.3 | 3.2 | 3.1 | 0.67 | 1.8 | 0.3 | 0.9 | 0.55 |
| E36-1 | 125 | 34.6 | 20.7 | 35.1 | 0.7 | 28.0 | 4.3 | 11.3 | 0.6 | 25.4 | 2.9 | 2.8 | 0.68 | 2.5 | 0.2 | 1.1 | 0.41 |
| IS15401 | 113 | 34.3 | 22.0 | 33.6 | 0.72 | 28.9 | 3.4 | 14.9 | 0.48 | 23.5 | 3.9 | 2.5 | 0.76 | 2.0 | 0.2 | 0.8 | 0.44 |
| IS23540 | 61 | 40.2 | 25.4 | 26.5 | 0.79 | 28.3 | 1.6 | 12.7 | 0.34 | 24.4 | 5.0 | 2.2 | 0.82 | 2.3 | 0.0 | 1.3 | 0.04 |
| B35 | 118 | 38.1 | 36.4 | 36.0 | 0.8 | 27.6 | 5.7 | 13.0 | 0.64 | 23.7 | 4.6 | 1.4 | 0.86 | 1.5 | 0.2 | 0.5 | 0.6 |
| Kono. | 134 | 35.0 | 36.8 | 25.5 | 0.85 | 29.1 | 8.0 | 12.7 | 0.72 | 26.8 | 6.1 | 3.7 | 0.77 | 2.1 | 0.2 | 0.9 | 0.44 |
| SC566-14 | 142 | 39.7 | 60.6 | 39.5 | 0.86 | 28.5 | 5.5 | 16.5 | 0.57 | 24.1 | 2.4 | 2.0 | 0.7 | 2.0 | 0.2 | 0.7 | 0.46 |
| Framida | 140 | 36.5 | 24.3 | 19.4 | 0.83 | 28.6 | 6.8 | 9.5 | 0.74 | 24.5 | 2.0 | 3.6 | 0.52 | 2.2 | 0.1 | 0.7 | 0.35 |
| CSM417 | 160 | 40.4 | 45.2 | 35.9 | 0.83 | 30.8 | 5.1 | 22.3 | 0.48 |  |  |  |  | 2.1 | 0.2 | 0.9 | 0.41 |
| CSM63 | 67 | 41.4 | 57.0 | 32.2 | 0.88 | 31.6 | 3.9 | 23.8 | 0.4 | 23.1 | 1.6 | 4.0 | 0.44 | 1.9 | 0.0 | 1.0 | 0 |
| CSM388 | 149 | 40.5 | 30.6 | 41.5 | 0.75 | 32.0 | 5.2 | 21.0 | 0.5 | 23.2 | 1.7 | 3.1 | 0.52 | 2.2 | 0.1 | 1.0 | 0.28 |
| Gadiaba | 135 | 38.3 | 41.8 | 42.0 | 0.8 | 27.5 | 7.7 | 11.1 | 0.74 | 23.7 | 2.7 | 2.6 | 0.67 | 2.2 | 0.1 | 0.7 | 0.35 |
| Wth Kaur | 137 | 39.5 | 87.6 | 31.2 | 0.92 | 28.7 | 4.6 | 12.8 | 0.59 | 24.3 | 4.7 | 3.7 | 0.72 | 2.2 | 0.2 | 0.7 | 0.54 |
| Average | 123 | 38.1 |  |  | 0.81 | 29.3 |  |  | 0.57 | 24.3 |  |  | 0.68 | 2.1 |  |  | 0.37 |

***Kenin-Keni sub-population characterized in 2012***

|  |  | Flag leaf [d] | | | | Plant height [cm] | | | | Number of internodes | | | | Average internode length [cm] | | | |
| --- | --- | --- | --- | --- | --- | --- | --- | --- | --- | --- | --- | --- | --- | --- | --- | --- | --- |
| ***Keni-Keni*** | N | Av | $\sigma_{g}^{2}$ | $\sigma_{ge}^{2}$ | $h^{2}$ | Av | $\sigma_{g}^{2}$ | $\sigma_{ge}^{2}$ | $h^{2}$ | Av | $\sigma_{g}^{2}$ | $\sigma_{ge}^{2}$ | $h^{2}$ | Av | $\sigma_{g}^{2}$ | $\sigma_{ge}^{2}$ | $h^{2}$ |
| Fara | 134 | 79.1 | 34.0 | 7.0 | 0.95 | 245.7 | 705.2 | 689.7 | 0.8 | 13.5 | 1.3 | 3.4 | 0.60 | 11.1 | 2.9 | 2.2 | 0.80 |
| E36-1 | 144 | 70.0 | 37.8 | 11.1 | 0.93 | 207.9 | 1044.0 | 758.4 | 0.85 | 11.9 | 1.6 | 3.8 | 0.63 | 10.4 | 4.9 | 2.6 | 0.86 |
| IS15401 | 138 | 75.0 | 23.3 | 9.0 | 0.91 | 223.1 | 642.9 | 577.3 | 0.82 | 12.8 | 0.5 | 3.8 | 0.34 | 10.4 | 2.5 | 2.1 | 0.77 |
| B35 | 79 | 71.6 | 17.0 | 19.3 | 0.78 | 205.4 | 898.9 | 1324.1 | 0.73 | 11.9 | 1.6 | 4.9 | 0.56 | 9.6 | 4.9 | 3.6 | 0.82 |
| CSM417 | 80 | 71.4 | 1.7 | 6.3 | 0.52 | 241.7 | 441.5 | 1040.8 | 0.63 | 11.5 | 0.0 | 1.5 | 0.02 | 12.0 | 3.1 | 5.1 | 0.69 |
| Average | 115 | 73.4 |  |  | 0.82 | 224.8 |  |  | 0.77 | 12.3 |  |  | 0.4 | 10.7 |  |  | 0.8 |

|  |  | Peduncle length [cm] | | | | Panicle length [cm] | | | | Grain weight [g] | | | | Grain yield [ton/ha] | | | |
| --- | --- | --- | --- | --- | --- | --- | --- | --- | --- | --- | --- | --- | --- | --- | --- | --- | --- |
| ***Keni-Keni*** | N | Av | $\sigma_{g}^{2}$ | $\sigma_{ge}^{2}$ | $h^{2}$ | Av | $\sigma_{g}^{2}$ | $\sigma_{ge}^{2}$ | $h^{2}$ | Av | $\sigma_{g}^{2}$ | $\sigma_{ge}^{2}$ | $h^{2}$ | Av | $\sigma_{g}^{2}$ | $\sigma_{ge}^{2}$ | $h^{2}$ |
| Fara | 134 | 59.7 | 20.1 | 55.4 | 0.59 | 37.3 | 3.5 | 26.7 | 0.34 | 22.9 | 3.8 | 3.0 | 0.72 | 2.0 | 0.3 | 0.7 | 0.6 |
| E36-1 | 144 | 51.3 | 69.0 | 43.0 | 0.87 | 35.1 | 7.9 | 23.6 | 0.57 | 21.0 | 2.8 | 2.2 | 0.71 | 2.1 | 0.1 | 0.7 | 0.39 |
| IS15401 | 138 | 55.7 | 25.2 | 45.3 | 0.69 | 35.6 | 4.3 | 29.6 | 0.37 | 22.7 | 2.3 | 2.5 | 0.65 | 2.2 | 0.1 | 0.8 | 0.27 |
| B35 | 79 | 55.1 | 51.4 | 61.6 | 0.77 | 36.6 | 3.4 | 39.4 | 0.26 | 20.3 | 4.0 | 4.2 | 0.65 | 1.7 | 0.2 | 0.4 | 0.65 |
| CSM417 | 80 | 64.4 | 10.1 | 44.8 | 0.47 | 40.0 | 5.7 | 41.4 | 0.36 |  |  |  |  | 2.4 | 0.0 | 1.1 | 0 |
| Average | 115 | 57.2 |  |  | 0.68 | 36.9 |  |  | 0.38 | 21.7 |  |  | 0.68 | 2.1 |  |  | 0.38 |

***Grinkan sub-population characterized in 2013***

|  |  | Flag leaf [d] | | | | Plant height [cm] | | | | Number of internodes | | | | Average internode length [cm] | | | |
| --- | --- | --- | --- | --- | --- | --- | --- | --- | --- | --- | --- | --- | --- | --- | --- | --- | --- |
| ***Grinkan*** | N | Av | $\sigma_{g}^{2}$ | $\sigma_{ge}^{2}$ | $h^{2}$ | Av | $\sigma_{g}^{2}$ | $\sigma_{ge}^{2}$ | $h^{2}$ | Av | $\sigma_{g}^{2}$ | $\sigma_{ge}^{2}$ | $h^{2}$ | Av | $\sigma_{g}^{2}$ | $\sigma_{ge}^{2}$ | $h^{2}$ |
| E36-1 | 46 | 70.2 | 31.7 | 7.1 | 0.95 | 155.29 | 395.35 | 262 | 0.86 | 13.0 | 1.5 | 2.1 | 0.74 | 7.4 | 1.3 | 0.3 | 0.81 |
| V33/08 | 67 | 73.1 | 6.5 | 6.1 | 0.81 | 157.43 | 655.28 | 555.2 | 0.83 | 13.1 | 0.0 | 3.1 | 0.00 | 7.0 | 2.3 | 0.1 | 0.83 |
| Kalaban | 115 | 70.4 | 19.2 | 7.1 | 0.92 | 153.5 | 461.43 | 318.5 | 0.85 | 12.8 | 1.1 | 2.2 | 0.66 | 7.2 | 1.7 | 0.2 | 0.81 |
| Malisor | 95 | 72.3 | 14.7 | 17.6 | 0.77 | 142.77 | 139.03 | 259.7 | 0.68 | 12.5 | 1.0 | 2.5 | 0.62 | 6.8 | 0.5 | 0.3 | 0.58 |
| BimbG | 100 | 79 | 15.2 | 10.9 | 0.85 | 177.86 | 1001.7 | 603.9 | 0.87 | 14.3 | 1.3 | 3.4 | 0.61 | 7.9 | 3.9 | 0.3 | 0.86 |
| Hafijega | 84 | 71.6 | 25.2 | 10.3 | 0.91 | 165.26 | 1310.7 | 614.2 | 0.9 | 12.3 | 0.4 | 2.4 | 0.43 | 8.0 | 7.3 | 1.0 | 0.91 |
| CSM_388 | 47 | 69.5 | 24.5 | 6.5 | 0.94 | 185.67 | 1540.3 | 661 | 0.9 | 12.7 | 1.3 | 2.3 | 0.69 | 9.4 | 8.2 | 1.6 | 0.91 |
| Sh Kaur | 50 | 73.9 | 5.1 | 5.6 | 0.79 | 161.13 | 843.1 | 618.5 | 0.85 | 14.0 | 0.0 | 4.2 | 0.00 | 7.1 | 3.0 | 0.1 | 0.86 |
| average | 75.5 | 72.5 |  |  | 0.87 | 162.36 |  |  | 0.84 | 13.1 |  |  | 0.47 | 7.6 |  |  | 0.82 |

|  |  | Peduncle length [cm] | | | | Panicle length [cm] | | | | Grain weight [g] | | | | Grain yield [ton/ha] | | | |
| --- | --- | --- | --- | --- | --- | --- | --- | --- | --- | --- | --- | --- | --- | --- | --- | --- | --- |
| ***Grinkan*** | N | Av | $\sigma_{g}^{2}$ | $\sigma_{ge}^{2}$ | $h^{2}$ | Av | $\sigma_{g}^{2}$ | $\sigma_{ge}^{2}$ | $h^{2}$ | Av | $\sigma_{g}^{2}$ | $\sigma_{ge}^{2}$ | $h^{2}$ | Av | $\sigma_{g}^{2}$ | $\sigma_{ge}^{2}$ | $h^{2}$ |
| E36-1 | 46 | 31.6 | 13.8 | 15.4 | 0.78 | 27.1 | 5.07 | 5.83 | 0.78 |  |  |  |  | 1.9 | 0.04 | 0.42 | 0.29 |
| V33/08 | 67 | 36.1 | 35.06 | 44.9 | 0.76 | 29.9 | 7.63 | 9.1 | 0.77 |  |  |  |  | 1.5 | 0.05 | 0.46 | 0.29 |
| Kalaban | 115 | 35.2 | 11.75 | 28.9 | 0.62 | 26.7 | 2.02 | 9.09 | 0.47 |  |  |  |  | 1.68 | 0.05 | 0.44 | 0.31 |
| Malisor | 95 | 31.9 | 18.73 | 19.4 | 0.79 | 26.7 | 2.53 | 8.9 | 0.53 | 17.1 | 5.6 | 42.1 | 0.21 | 1.68 | 0.02 | 0.41 | 0.18 |
| BimbG | 100 | 35.9 | 23.85 | 28.3 | 0.77 | 30.5 | 10.9 | 10.44 | 0.81 | 19.3 | 16.2 | 23.4 | 0.58 | 1.09 | 0.22 | 0.35 | 0.71 |
| Hafijega | 84 | 35.7 | 34.89 | 26.8 | 0.84 | 31.2 | 9.57 | 11.31 | 0.77 |  |  |  |  | 1.27 | 0.1 | 0.38 | 0.53 |
| CSM_388 | 47 | 37.9 | 44.31 | 27.7 | 0.86 | 30.1 | 5.53 | 8.04 | 0.73 |  |  |  |  | 1.73 | 0.07 | 0.57 | 0.33 |
| Sh Kaur | 50 | 34.8 | 35.49 | 41.8 | 0.77 | 29 | 3.46 | 12.68 | 0.52 |  |  |  |  | 1.39 | 0.19 | 0.45 | 0.63 |
| average | 75.5 | 34.9 |  |  | 0.77 | 28.9 |  |  | 0.67 | 18.2 |  |  | 0.39 | 1.53 |  |  | 0.41 |

***Kenin-Keni sub-population characterized in 2013***

|  |  | Flag leaf [d] | | | | Plant height [cm] | | | | Number of internodes | | | | Average internode length [cm] | | | |
| --- | --- | --- | --- | --- | --- | --- | --- | --- | --- | --- | --- | --- | --- | --- | --- | --- | --- |
|  | N | Av | $\sigma_{g}^{2}$ | $\sigma_{ge}^{2}$ | $h^{2}$ | Av | $\sigma_{g}^{2}$ | $\sigma_{ge}^{2}$ | $h^{2}$ | Av | $\sigma_{g}^{2}$ | $\sigma_{ge}^{2}$ | $h^{2}$ | Av | $\sigma_{g}^{2}$ | $\sigma_{ge}^{2}$ | $h^{2}$ |
| Sangatigui | 35 | 68.2 | 28.3 | 8.7 | 0.93 | 213.36 | 477.75 | 358.7 | 0.84 | 11.6 | 1.8 | 2.5 | 0.72 | 10.1 | 3.3 | 2.0 | 0.85 |
| IS23540 | 67 | 70.2 | 25.1 | 12.7 | 0.89 | 210.72 | 297.61 | 430.5 | 0.73 | 12.6 | 1.5 | 1.2 | 0.73 | 10.0 | 1.3 | 2.3 | 0.66 |
| Kalaban | 80 | 67.2 | 16.7 | 8.6 | 0.89 | 196 | 928.98 | 367.6 | 0.91 | 11.4 | 1.8 | 1.8 | 0.77 | 9.7 | 4.1 | 2.3 | 0.87 |
| Malisor | 66 | 69.8 | 20.9 | 22.8 | 0.79 | 182.62 | 769.19 | 421.5 | 0.88 | 12.0 | 0.9 | 7.2 | 0.33 | 8.3 | 3.0 | 1.5 | 0.82 |
| BimbG | 73 | 74.9 | 36.3 | 10.5 | 0.93 | 218.56 | 546.97 | 414 | 0.84 | 12.9 | 1.8 | 2.5 | 0.73 | 9.8 | 2.9 | 2.2 | 0.83 |
| CSM417 | 23 | 66.5 | 0.55 | 6.5 | 0.25 | 223.3 | 407 | 376.8 | 0.81 | 11.6 | 0.5 | 0.3 | 0.59 | 10.6 | 2.7 | 2.2 | 0.81 |
| average | 57.3 | 69.5 |  |  | 0.78 | 207.43 |  |  | 0.84 | 12.0 |  |  | 0.64 | 9.7 |  |  | 0.81 |

|  |  | Peduncle length [cm] | | | | Panicle length [cm] | | | | Grain weight [g] | | | | Grain yield [ton/ha] | | | |
| --- | --- | --- | --- | --- | --- | --- | --- | --- | --- | --- | --- | --- | --- | --- | --- | --- | --- |
|  | N | Av | $\sigma_{g}^{2}$ | $\sigma_{ge}^{2}$ | $h^{2}$ | Av | $\sigma_{g}^{2}$ | $\sigma_{ge}^{2}$ | $h^{2}$ | Av | $\sigma_{g}^{2}$ | $\sigma_{ge}^{2}$ | $h^{2}$ | Av | $\sigma_{g}^{2}$ | $\sigma_{ge}^{2}$ | $h^{2}$ |
| Sangatigui | 35 | 57.2 | 17.01 | 50.2 | 0.58 | 39.99 | 4.97 | 15.58 | 0.56 |  |  |  |  | 1.49 | 0.14 | 0.46 | 0.55 |
| IS23540 | 67 | 48.7 | 40.06 | 41.4 | 0.79 | 34.84 | 6.72 | 12.46 | 0.68 |  |  |  |  | 1.54 | 0.13 | 0.44 | 0.54 |
| Kalaban | 80 | 50.2 | 47.95 | 42.3 | 0.82 | 34.79 | 10.6 | 13.87 | 0.75 |  |  |  |  | 1.71 | 0.06 | 0.53 | 0.3 |
| Malisor | 66 | 48.7 | 54.06 | 28.5 | 0.88 | 34.85 | 8.45 | 16.75 | 0.67 | 15.3 | 2.2 | 19.8 | 0.18 | 1.4 | 0.12 | 0.53 | 0.47 |
| BimbG | 73 | 52.3 | 13.23 | 57.8 | 0.48 | 39.82 | 5.1 | 19.71 | 0.51 | 16.4 | 0 | 38.1 | 0 | 1.43 | 0.33 | 0.57 | 0.7 |
| CSM417 | 23 | 59.6 | 31.63 | 65.2 | 0.66 | 40.91 | 13.2 | 12.68 | 0.81 |  |  |  |  | 2.06 | 0.25 | 0.37 | 0.73 |
| average | 57.3 | 52.8 |  |  | 0.7 | 37.53 |  |  | 0.66 | 15.8 |  |  | 0.09 | 1.61 |  |  | 0.55 |

***Lata3 sub-population characterized in 2013***

|  |  | Flag leaf [d] | | | | Plant height [cm] | | | | Number of internodes | | | | Average internode length [cm] | | | |
| --- | --- | --- | --- | --- | --- | --- | --- | --- | --- | --- | --- | --- | --- | --- | --- | --- | --- |
| ***Lata*** | N | Av | $\sigma_{g}^{2}$ | $\sigma_{ge}^{2}$ | $h^{2}$ | Av | $\sigma_{g}^{2}$ | $\sigma_{ge}^{2}$ | $h^{2}$ | Av | $\sigma_{g}^{2}$ | $\sigma_{ge}^{2}$ | $h^{2}$ | Av | $\sigma_{g}^{2}$ | $\sigma_{ge}^{2}$ | $h^{2}$ |
| Grinkan | 95 | 81.2 | 24.7 | 0.3 | 0.97 | 238.2 | 1596.8 | 105.8 | 0.93 | 6.8 | 0.5 | 0.0 | 0.62 | 30.2 | 39.3 | 2.2 | 0.94 |
| IS23645 | 54 | 83.2 | 40.3 | 0.9 | 0.97 | 250.6 | 3387.6 | 1.9 | 0.97 | 5.9 | 0.6 | 0.0 | 0.72 | 27.1 | 44.6 | 2.2 | 0.93 |
| SK5912 | 76 | 86.0 | 34.4 | 1.4 | 0.97 | 248.9 | 1170.1 | 74.6 | 0.92 | 6.4 | 0.9 | 0.0 | 0.61 | 28.7 | 37.8 | 2.2 | 0.94 |
| DouaG | 72 | 83.7 | 24.9 | 0.9 | 0.97 | 276.9 | 558.5 | 0.0 | 0.87 | 5.9 | 0.4 | 0.0 | 0.63 | 31.5 | 18.2 | 2.2 | 0.87 |
| Framida | 60 | 81.6 | 56.6 | 1.3 | 0.98 | 245.2 | 369.7 | 0.0 | 0.78 | 6.7 | 0.7 | 0.0 | 0.74 | 25.8 | 10.2 | 2.2 | 0.79 |
| Gnossiconi | 67 | 79.9 | 18.1 | 0.6 | 0.96 | 269.5 | 269.8 | 64.0 | 0.74 | 6.2 | 0.2 | 0.0 | 0.45 | 30.5 | 11.4 | 2.2 | 0.82 |
| IS15401 | 89 | 85.2 | 49.1 | 1.6 | 0.97 | 274.7 | 799.7 | 7.5 | 0.91 | 6.1 | 1.2 | 0.0 | 0.81 | 28.5 | 18.3 | 2.2 | 0.88 |
| IS23540 | 68 | 82.0 | 52.9 | 3.6 | 0.96 | 237.7 | 1011.8 | 20.2 | 0.93 | 6.6 | 0.7 | 0.0 | 0.70 | 29.4 | 26.9 | 2.2 | 0.91 |
| Fara | 82 | 90.0 | 42.9 | 0.9 | 0.97 | 295.2 | 924.6 | 70.8 | 0.86 | 6.1 | 1.1 | 0.0 | 0.77 | 27.1 | 15.5 | 2.2 | 0.86 |
| Ngolofing | 76 | 82.0 | 2.1 | 0.0 | 0.8 | 275.8 | 331.2 | 0.0 | 0.82 | 5.6 | 0.2 | 0.0 | 0.52 | 29.4 | 14.6 | 2.2 | 0.86 |
| Sambalma | 88 | 86.3 | 31.2 | 0.0 | 0.97 | 265.9 | 628.5 | 32.5 | 0.88 | 6.1 | 0.6 | 0.0 | 0.66 | 28.2 | 15.7 | 2.2 | 0.86 |
| SC566-14 | 69 | 82.5 | 74.7 | 5.8 | 0.96 | 253.5 | 1465.7 | 124.1 | 0.92 | 5.8 | 0.9 | 0.0 | 0.71 | 32.1 | 20.7 | 2.2 | 0.87 |
| average | 74 | 83.6 |  |  | 0.95 | 261 |  |  | 0.88 | 6.2 |  |  | 0.66 | 29.0 |  |  | 0.88 |

|  |  | Peduncle length [cm] | | | | Panicle length [cm] | | | | Grain weight [g] | | | | Grain yield [ton/ha] | | | |
| --- | --- | --- | --- | --- | --- | --- | --- | --- | --- | --- | --- | --- | --- | --- | --- | --- | --- |
| ***Lata*** | N | Av | $\sigma_{g}^{2}$ | $\sigma_{ge}^{2}$ | $h^{2}$ | Av | $\sigma_{g}^{2}$ | $\sigma_{ge}^{2}$ | $h^{2}$ | Av | $\sigma_{g}^{2}$ | $\sigma_{ge}^{2}$ | $h^{2}$ | Av | $\sigma_{g}^{2}$ | $\sigma_{ge}^{2}$ | $h^{2}$ |
| Grinkan | 95 | 42.8 | 27.2 | 1.4 | 0.88 | 29.2 | 6.1 | 1.3 | 0.77 | 2.1 | 0.04 | 0.01 | 0.86 | 2.47 | 0.31 | 0.05 | 0.8 |
| IS23645 | 54 | 41.0 | 49.1 | 2.1 | 0.89 | 30.8 | 9.8 | 0.0 | 0.84 | 1.86 | 0.08 | 0.01 | 0.92 | 1.46 | 0.22 | 0.09 | 0.78 |
| SK5912 | 76 | 43.4 | 27.6 | 0.3 | 0.9 | 28.1 | 6.1 | 1.7 | 0.73 | 2.33 | 0.09 | 0.01 | 0.91 | 2.07 | 0.21 | 0.08 | 0.77 |
| DouaG | 72 | 48.6 | 23.2 | 0.3 | 0.87 | 30.5 | 4.4 | 0.0 | 0.75 | 2.13 | 0.05 | 0 | 0.95 | 2.32 | 0.09 | 0.07 | 0.56 |
| Framida | 60 | 42.2 | 33.1 | 0.8 | 0.92 | 28.0 | 6.4 | 1.0 | 0.76 | 2.28 | 0.07 | 0.01 | 0.91 | 2.01 | 0.14 | 0.16 | 0.56 |
| Gnossiconi | 67 | 46.5 | 14.1 | 0.0 | 0.77 | 29.2 | 3.6 | 0.0 | 0.68 | 2.24 | 0.04 | 0 | 0.89 | 2.37 | 0.05 | 0.05 | 0.4 |
| IS15401 | 89 | 43.8 | 24.3 | 4.3 | 0.83 | 28.5 | 5.9 | 0.0 | 0.87 | 2.36 | 0.06 | 0.01 | 0.9 | 2.45 | 0.19 | 0.13 | 0.67 |
| IS23540 | 68 | 39.8 | 30.0 | 2.9 | 0.88 | 26.0 | 4.6 | 1.6 | 0.69 | 2.24 | 0.08 | 0.01 | 0.91 | 2.18 | 0.11 | 0.06 | 0.6 |
| Fara | 82 | 47.2 | 22.9 | 0.0 | 0.86 | 32.2 | 7.6 | 0.5 | 0.78 | 2.25 | 0.07 | 0.01 | 0.91 | 2.07 | 0.18 | 0.05 | 0.74 |
| Ngolofing | 76 | 44.9 | 12.9 | 0.0 | 0.81 | 30.8 | 3.1 | 0.0 | 0.66 | 2.2 | 0.02 | 0.01 | 0.76 | 2.28 | 0.03 | 0.09 | 0.31 |
| Sambalma | 88 | 43.0 | 18.0 | 1.7 | 0.84 | 32.7 | 7.3 | 0.9 | 0.78 | 2.27 | 0.04 | 0.01 | 0.84 | 2.46 | 0.14 | 0.07 | 0.65 |
| SC566-14 | 69 | 43.0 | 28.3 | 0.3 | 0.89 | 28.1 | 10.9 | 0.1 | 0.88 | 2.22 | 0.04 | 0.01 | 0.88 | 2.21 | 0.28 | 0.07 | 0.78 |
| average | 74 | 43.9 |  |  | 0.9 | 29.5 |  |  | 0.77 | 2.21 |  |  | 0.89 | 2.2 |  |  | 0.64 |

**Table S10**: Lists of the five most influential environmental covariables for each trait

Table of most influential EC on traits with recurrent parent, year of phenotyping, trait, most influential EC in order of influence, average R2 trait-EC of the different tested window, R2 trait-EC of the best window, starting and ending days of the most influencing window, value of the EC in the environment during the best window

| **RP** | **year** | **trait** | **EC** | **R2_av** | **R2_win** | **start** | **end** | **SB1** | **SB2** | **CZ1** | **CZ2** |
| --- | --- | --- | --- | --- | --- | --- | --- | --- | --- | --- | --- |
| Grinkan | 2012 | FLAG | photoperiod | 0.997 | 1 | 28 | 67 | 12.4 | 12.1 | 12.4 | 12.2 |
| Grinkan | 2012 | FLAG | solarRad | 0.844 | 0.999 | 9 | 88 | 3003.5 | 2928 | 3002.5 | 2937.1 |
| Grinkan | 2012 | FLAG | Trange | 0.82 | 1 | 3 | 82 | 8.8 | 9.4 | 8.9 | 9.4 |
| Grinkan | 2012 | FLAG | hSun | 0.629 | 0.997 | 1 | 80 | 512.1 | 542.9 | 511.3 | 538 |
| Grinkan | 2012 | FLAG | rain | 0.378 | 0.959 | 17 | 76 | 573.6 | 417.4 | 540.4 | 408.7 |
|  |  |  |  |  |  |  |  |  |  |  |  |
| Grinkan | 2012 | PH | photoperiod | 0.775 | 0.895 | 1 | 20 | 12.7 | 12.5 | 12.7 | 12.6 |
| Grinkan | 2012 | PH | solarRad | 0.746 | 0.995 | 23 | 42 | 756.5 | 752.6 | 758.9 | 754.5 |
| Grinkan | 2012 | PH | Trange | 0.628 | 0.985 | 6 | 65 | 8.8 | 8.9 | 8.7 | 8.8 |
| Grinkan | 2012 | PH | hSun | 0.621 | 0.999 | 16 | 75 | 389.4 | 407.2 | 376.7 | 398.8 |
| Grinkan | 2012 | PH | hum | 0.523 | 0.999 | 14 | 113 | 77.2 | 74 | 79.8 | 75.4 |
|  |  |  |  |  |  |  |  |  |  |  |  |
| Grinkan | 2012 | N_N | photoperiod | 0.819 | 0.915 | 1 | 20 | 12.7 | 12.5 | 12.7 | 12.6 |
| Grinkan | 2012 | N_N | solarRad | 0.758 | 0.901 | 1 | 60 | 2266.3 | 2248.7 | 2272.4 | 2255 |
| Grinkan | 2012 | N_N | Trange | 0.633 | 0.974 | 46 | 65 | 8.8 | 9.8 | 9 | 9.3 |
| Grinkan | 2012 | N_N | hSun | 0.62 | 0.994 | 7 | 66 | 376.3 | 396.7 | 373.6 | 383.1 |
| Grinkan | 2012 | N_N | hum | 0.388 | 0.841 | 19 | 118 | 76.9 | 73.1 | 78.8 | 74.2 |
|  |  |  |  |  |  |  |  |  |  |  |  |
| Grinkan | 2012 | N_L | FRUE | 0.32 | 0.956 | 34 | 113 | 1 | 1 | 1 | 1 |
| Grinkan | 2012 | N_L | rain | 0.291 | 0.967 | 9 | 48 | 359.8 | 419.7 | 436.6 | 396.7 |
| Grinkan | 2012 | N_L | PETP | 0.275 | 0.963 | 5 | 24 | 4.7 | -0.5 | -1.8 | 1.4 |
| Grinkan | 2012 | N_L | ETP | 0.168 | 0.644 | 74 | 93 | 9.1 | 9.9 | 10.3 | 10.1 |
| Grinkan | 2012 | N_L | hSun | 0.145 | 0.919 | 22 | 41 | 137.2 | 120.2 | 114.8 | 124.6 |
|  |  |  |  |  |  |  |  |  |  |  |  |
| Grinkan | 2012 | PAN | PETP | 0.356 | 0.999 | 8 | 67 | -0.4 | 0.2 | 0.5 | -1.9 |
| Grinkan | 2012 | PAN | rain | 0.345 | 0.995 | 7 | 66 | 516.6 | 539.1 | 554.9 | 449.7 |
| Grinkan | 2012 | PAN | VPD | 0.322 | 0.634 | 52 | 71 | 0.8 | 1 | 0.9 | 1.3 |
| Grinkan | 2012 | PAN | Tmin | 0.295 | 0.549 | 68 | 87 | 31.3 | 33.2 | 32.4 | 35.8 |
| Grinkan | 2012 | PAN | hum | 0.277 | 0.924 | 7 | 26 | 78.6 | 78.6 | 77.9 | 81.9 |
|  |  |  |  |  |  |  |  |  |  |  |  |
| Grinkan | 2012 | PED | DD | 0.857 | 0.979 | 88 | 107 | 377 | 396.8 | 433.4 | 452.4 |
| Grinkan | 2012 | PED | SVP | 0.85 | 0.974 | 88 | 107 | 0.2 | 0.2 | 0.2 | 0.3 |
| Grinkan | 2012 | PED | Tmin | 0.809 | 0.993 | 19 | 78 | 30.6 | 31.3 | 31.9 | 32.4 |
| Grinkan | 2012 | PED | ETP | 0.788 | 1 | 53 | 112 | 9.1 | 9.5 | 9.9 | 10.1 |
| Grinkan | 2012 | PED | FRUE | 0.772 | 0.997 | 4 | 63 | 1 | 1 | 1 | 1 |
|  |  |  |  |  |  |  |  |  |  |  |  |
| Grinkan | 2012 | YIELD | Trange | 0.382 | 0.995 | 20 | 39 | 8.6 | 8.6 | 8.3 | 8.9 |
| Grinkan | 2012 | YIELD | hSun | 0.352 | 0.976 | 2 | 61 | 376.3 | 377 | 371.1 | 381.7 |
| Grinkan | 2012 | YIELD | hum | 0.346 | 0.869 | 69 | 88 | 76.4 | 74.5 | 83.2 | 72.4 |
| Grinkan | 2012 | YIELD | rain | 0.33 | 0.998 | 5 | 64 | 509.8 | 524.2 | 584.4 | 460.2 |
| Grinkan | 2012 | YIELD | photoperiod | 0.32 | 0.321 | 2 | 101 | 12.4 | 12.1 | 12.3 | 12.1 |

| **RP** | **year** | **trait** | **EC** | **R2_av** | **R2_win** | **start** | **end** | **SB1** | **SB2** | **CZ1** | **CZ2** |
| --- | --- | --- | --- | --- | --- | --- | --- | --- | --- | --- | --- |
| Grinkan | 2013 | FLAG | photoperiod | 0.92 | 0.926 | 15 | 34 | 12.6 | 12.4 | 12.5 | 12.3 |
| Grinkan | 2013 | FLAG | solarRad | 0.693 | 0.866 | 64 | 83 | 741.5 | 710.8 | 708.1 | 674.2 |
| Grinkan | 2013 | FLAG | hSun | 0.623 | 0.991 | 61 | 80 | 145 | 175.7 | 163.2 | 181.5 |
| Grinkan | 2013 | FLAG | hum | 0.584 | 0.993 | 9 | 28 | 75.9 | 80.7 | 78.3 | 81.8 |
| Grinkan | 2013 | FLAG | Trange | 0.514 | 0.954 | 58 | 77 | 8.2 | 10.4 | 9.7 | 12 |
|  |  |  |  |  |  |  |  |  |  |  |  |
| Grinkan | 2013 | PH | solarRad | 0.887 | 0.961 | 41 | 60 | 755.5 | 742.5 | 741.7 | 718.6 |
| Grinkan | 2013 | PH | photoperiod | 0.874 | 0.884 | 5 | 24 | 12.7 | 12.5 | 12.6 | 12.4 |
| Grinkan | 2013 | PH | Trange | 0.848 | 1 | 16 | 75 | 8.3 | 8.7 | 9.1 | 10.2 |
| Grinkan | 2013 | PH | VPD | 0.822 | 0.998 | 66 | 85 | 1 | 1.3 | 1.7 | 2.6 |
| Grinkan | 2013 | PH | hum | 0.814 | 0.998 | 45 | 64 | 80.7 | 79.1 | 77.9 | 74.6 |
|  |  |  |  |  |  |  |  |  |  |  |  |
| Grinkan | 2013 | N_N | photoperiod | 0.774 | 0.812 | 1 | 20 | 12.7 | 12.6 | 12.6 | 12.4 |
| Grinkan | 2013 | N_N | solarRad | 0.65 | 0.726 | 85 | 104 | 712.6 | 670 | 665.8 | 630.8 |
| Grinkan | 2013 | N_N | hSun | 0.565 | 0.995 | 2 | 21 | 155.6 | 130.1 | 147.8 | 128 |
| Grinkan | 2013 | N_N | Trange | 0.523 | 0.873 | 58 | 77 | 8.2 | 10.4 | 9.7 | 12 |
| Grinkan | 2013 | N_N | hum | 0.452 | 0.998 | 3 | 42 | 77.1 | 80 | 78.3 | 80.6 |
|  |  |  |  |  |  |  |  |  |  |  |  |
| Grinkan | 2013 | N_L | VPD | 0.781 | 0.976 | 4 | 63 | 1 | 1 | 1.3 | 1.3 |
| Grinkan | 2013 | N_L | SVP | 0.772 | 0.998 | 12 | 51 | 0.2 | 0.2 | 0.2 | 0.2 |
| Grinkan | 2013 | N_L | DD | 0.772 | 0.998 | 13 | 52 | 764.4 | 742.9 | 786.3 | 807.1 |
| Grinkan | 2013 | N_L | Tmin | 0.754 | 0.998 | 13 | 52 | 31.2 | 30.6 | 32.2 | 32.8 |
| Grinkan | 2013 | N_L | photothermal | 0.728 | 0.997 | 25 | 64 | 9380.2 | 9144.8 | 9741.9 | 9961.7 |
|  |  |  |  |  |  |  |  |  |  |  |  |
| Grinkan | 2013 | PAN | SVP | 0.814 | 0.994 | 11 | 50 | 0.2 | 0.2 | 0.2 | 0.2 |
| Grinkan | 2013 | PAN | DD | 0.813 | 0.991 | 11 | 50 | 762.5 | 740.9 | 791 | 804 |
| Grinkan | 2013 | PAN | VPD | 0.795 | 0.995 | 5 | 64 | 1 | 0.9 | 1.3 | 1.3 |
| Grinkan | 2013 | PAN | Tmin | 0.79 | 0.998 | 13 | 52 | 31.2 | 30.6 | 32.2 | 32.8 |
| Grinkan | 2013 | PAN | photothermal | 0.765 | 0.998 | 25 | 64 | 9380.2 | 9144.8 | 9741.9 | 9961.7 |
|  |  |  |  |  |  |  |  |  |  |  |  |
| Grinkan | 2013 | PED | SVP | 0.804 | 0.993 | 12 | 51 | 0.2 | 0.2 | 0.2 | 0.2 |
| Grinkan | 2013 | PED | DD | 0.803 | 0.991 | 20 | 59 | 755.8 | 739.9 | 784.7 | 812.5 |
| Grinkan | 2013 | PED | VPD | 0.801 | 0.989 | 5 | 64 | 1 | 0.9 | 1.3 | 1.3 |
| Grinkan | 2013 | PED | Tmin | 0.788 | 1 | 13 | 52 | 31.2 | 30.6 | 32.2 | 32.8 |
| Grinkan | 2013 | PED | photothermal | 0.75 | 0.998 | 25 | 64 | 9380.2 | 9144.8 | 9741.9 | 9961.7 |
|  |  |  |  |  |  |  |  |  |  |  |  |
| Grinkan | 2013 | YIELD | hum | 0.746 | 1 | 10 | 69 | 79.2 | 79.6 | 78.9 | 76.9 |
| Grinkan | 2013 | YIELD | VPD | 0.736 | 0.991 | 33 | 52 | 0.9 | 0.9 | 1.1 | 1.5 |
| Grinkan | 2013 | YIELD | solarRad | 0.693 | 0.893 | 21 | 40 | 756.5 | 755 | 755.8 | 744.7 |
| Grinkan | 2013 | YIELD | Trange | 0.651 | 0.986 | 24 | 43 | 8.3 | 7.8 | 8.5 | 9.7 |
| Grinkan | 2013 | YIELD | Tmin | 0.642 | 0.934 | 26 | 45 | 31.1 | 30.4 | 31.8 | 33.1 |

| **RP** | **year** | **trait** | **EC** | **R2_av** | **R2_win** | **start** | **end** | **SB1** | **SB2** | **CZ1** | **CZ2** |
| --- | --- | --- | --- | --- | --- | --- | --- | --- | --- | --- | --- |
| Kenin-K | 2012 | FLAG | photoperiod | 0.995 | 0.999 | 43 | 62 | 12.4 | 12.1 | 12.4 | 12.1 |
| Kenin-K | 2012 | FLAG | solarRad | 0.817 | 0.997 | 49 | 68 | 753.5 | 728.2 | 752.4 | 730.7 |
| Kenin-K | 2012 | FLAG | Trange | 0.8 | 1 | 3 | 82 | 8.8 | 9.4 | 8.9 | 9.4 |
| Kenin-K | 2012 | FLAG | hSun | 0.58 | 0.994 | 1 | 80 | 512.1 | 542.9 | 511.3 | 538 |
| Kenin-K | 2012 | FLAG | photothermal | 0.553 | 0.997 | 12 | 31 | 2600.1 | 2224.2 | 2598.4 | 2282.5 |
|  |  |  |  |  |  |  |  |  |  |  |  |
| Kenin-K | 2012 | PH | hum | 0.551 | 0.998 | 48 | 87 | 77.2 | 75.8 | 83.9 | 77.8 |
| Kenin-K | 2012 | PH | Tmax | 0.504 | 0.996 | 19 | 38 | 22.2 | 21.9 | 23.9 | 22.5 |
| Kenin-K | 2012 | PH | solarRad | 0.414 | 0.959 | 18 | 37 | 756 | 754.6 | 758.7 | 756.5 |
| Kenin-K | 2012 | PH | photoperiod | 0.371 | 0.523 | 1 | 20 | 12.7 | 12.5 | 12.7 | 12.6 |
| Kenin-K | 2012 | PH | hSun | 0.365 | 0.985 | 12 | 71 | 393.5 | 396.2 | 375.2 | 392.6 |
|  |  |  |  |  |  |  |  |  |  |  |  |
| Kenin-K | 2012 | N_N | photoperiod | 0.684 | 0.836 | 1 | 20 | 12.7 | 12.5 | 12.7 | 12.6 |
| Kenin-K | 2012 | N_N | solarRad | 0.668 | 0.994 | 9 | 48 | 1511.7 | 1505.7 | 1516.7 | 1509.6 |
| Kenin-K | 2012 | N_N | hSun | 0.54 | 0.994 | 15 | 74 | 389.3 | 405.2 | 380.1 | 395.2 |
| Kenin-K | 2012 | N_N | hum | 0.52 | 0.991 | 7 | 106 | 77.7 | 74.8 | 80.4 | 76.8 |
| Kenin-K | 2012 | N_N | Trange | 0.517 | 0.97 | 6 | 65 | 8.8 | 8.9 | 8.7 | 8.8 |
|  |  |  |  |  |  |  |  |  |  |  |  |
| Kenin-K | 2012 | N_L | FRUE | 0.593 | 0.972 | 3 | 22 | 1 | 1 | 1 | 1 |
| Kenin-K | 2012 | N_L | rain | 0.439 | 0.999 | 15 | 54 | 277 | 404.2 | 409.4 | 377.2 |
| Kenin-K | 2012 | N_L | PETP | 0.438 | 0.976 | 18 | 57 | -2.1 | 1 | 0.7 | 0.3 |
| Kenin-K | 2012 | N_L | ETP | 0.397 | 0.895 | 75 | 94 | 9.2 | 9.9 | 10.2 | 10 |
| Kenin-K | 2012 | N_L | Tmin | 0.344 | 0.55 | 47 | 66 | 30.5 | 31.8 | 31.6 | 32.5 |
|  |  |  |  |  |  |  |  |  |  |  |  |
| Kenin-K | 2012 | PAN | photoperiod | 0.937 | 0.941 | 33 | 52 | 12.5 | 12.2 | 12.5 | 12.2 |
| Kenin-K | 2012 | PAN | solarRad | 0.843 | 0.932 | 56 | 75 | 749.7 | 717.6 | 747.4 | 720.2 |
| Kenin-K | 2012 | PAN | Trange | 0.802 | 0.999 | 56 | 75 | 8.8 | 10.2 | 9.2 | 9.8 |
| Kenin-K | 2012 | PAN | hSun | 0.714 | 0.995 | 53 | 72 | 131.9 | 147.5 | 133.9 | 142.4 |
| Kenin-K | 2012 | PAN | photothermal | 0.544 | 0.986 | 17 | 36 | 2676.2 | 2350.1 | 2577.4 | 2425.7 |
|  |  |  |  |  |  |  |  |  |  |  |  |
| Kenin-K | 2012 | PED | rain | 0.268 | 1 | 34 | 53 | 162.9 | 255.4 | 262.2 | 131 |
| Kenin-K | 2012 | PED | PETP | 0.261 | 0.999 | 37 | 56 | -2 | 3.1 | 3.9 | -3.5 |
| Kenin-K | 2012 | PED | hum | 0.131 | 0.601 | 7 | 26 | 78.6 | 78.6 | 77.9 | 81.9 |
| Kenin-K | 2012 | PED | photothermal | 0.127 | 0.982 | 27 | 46 | 2525.5 | 2194.5 | 2236.6 | 2627.2 |
| Kenin-K | 2012 | PED | hSun | 0.113 | 0.989 | 23 | 62 | 258.3 | 251.5 | 249.5 | 261.6 |
|  |  |  |  |  |  |  |  |  |  |  |  |
| Kenin-K | 2012 | YIELD | FRUE | 0.385 | 0.964 | 14 | 113 | 1 | 1 | 1 | 1 |
| Kenin-K | 2012 | YIELD | Tmax | 0.305 | 0.736 | 12 | 31 | 21.8 | 22 | 23.9 | 22.9 |
| Kenin-K | 2012 | YIELD | ETP | 0.288 | 0.886 | 7 | 26 | 8.6 | 8.9 | 10 | 8.8 |
| Kenin-K | 2012 | YIELD | hum | 0.267 | 0.774 | 59 | 78 | 78 | 78 | 84.6 | 79 |
| Kenin-K | 2012 | YIELD | DD | 0.254 | 0.724 | 7 | 26 | 358.5 | 364 | 403.8 | 384.4 |

| **RP** | **year** | **trait** | **EC** | **R2_av** | **R2_win** | **start** | **end** | **SB1** | **SB2** | **CZ1** | **CZ2** |
| --- | --- | --- | --- | --- | --- | --- | --- | --- | --- | --- | --- |
| Kenin-K | 2013 | FLAG | photoperiod | 0.975 | 0.978 | 4 | 43 | 12.6 | 12.4 | 12.5 | 12.3 |
| Kenin-K | 2013 | FLAG | solarRad | 0.842 | 0.999 | 55 | 74 | 749.1 | 725.3 | 723.4 | 692.8 |
| Kenin-K | 2013 | FLAG | hSun | 0.69 | 0.996 | 14 | 73 | 416.7 | 442.1 | 446.7 | 487.8 |
| Kenin-K | 2013 | FLAG | Trange | 0.686 | 0.998 | 36 | 75 | 8.1 | 9.2 | 9.4 | 10.7 |
| Kenin-K | 2013 | FLAG | hum | 0.645 | 0.999 | 49 | 68 | 80.7 | 77.7 | 77 | 72.8 |
|  |  |  |  |  |  |  |  |  |  |  |  |
| Kenin-K | 2013 | PH | photoperiod | 0.966 | 0.972 | 23 | 42 | 12.6 | 12.3 | 12.4 | 12.2 |
| Kenin-K | 2013 | PH | solarRad | 0.83 | 0.945 | 88 | 107 | 707.3 | 663.8 | 659.4 | 625.2 |
| Kenin-K | 2013 | PH | Trange | 0.714 | 0.976 | 58 | 77 | 8.2 | 10.4 | 9.7 | 12 |
| Kenin-K | 2013 | PH | hSun | 0.707 | 0.999 | 54 | 73 | 141.3 | 162.2 | 157.5 | 172.8 |
| Kenin-K | 2013 | PH | hum | 0.627 | 0.961 | 9 | 28 | 75.9 | 80.7 | 78.3 | 81.8 |
|  |  |  |  |  |  |  |  |  |  |  |  |
| Kenin-K | 2013 | N_N | photoperiod | 0.769 | 0.773 | 32 | 51 | 12.5 | 12.2 | 12.3 | 12 |
| Kenin-K | 2013 | N_N | solarRad | 0.584 | 0.737 | 90 | 109 | 703.7 | 659.7 | 655.2 | 621.6 |
| Kenin-K | 2013 | N_N | hSun | 0.581 | 1 | 67 | 86 | 159.1 | 176.1 | 167.9 | 175.9 |
| Kenin-K | 2013 | N_N | Trange | 0.487 | 0.808 | 58 | 77 | 8.2 | 10.4 | 9.7 | 12 |
| Kenin-K | 2013 | N_N | hum | 0.401 | 0.999 | 5 | 44 | 77.4 | 80.2 | 78.8 | 80.2 |
|  |  |  |  |  |  |  |  |  |  |  |  |
| Kenin-K | 2013 | N_L | photoperiod | 0.956 | 0.96 | 1 | 80 | 12.5 | 12.2 | 12.3 | 12.1 |
| Kenin-K | 2013 | N_L | solarRad | 0.911 | 0.991 | 55 | 74 | 749.1 | 725.3 | 723.4 | 692.8 |
| Kenin-K | 2013 | N_L | Trange | 0.852 | 1 | 25 | 104 | 8.8 | 10.1 | 10.6 | 12.4 |
| Kenin-K | 2013 | N_L | hum | 0.777 | 0.998 | 49 | 68 | 80.7 | 77.7 | 77 | 72.8 |
| Kenin-K | 2013 | N_L | Tmin | 0.776 | 0.99 | 62 | 81 | 31 | 33 | 34.3 | 36.7 |
|  |  |  |  |  |  |  |  |  |  |  |  |
| Kenin-K | 2013 | PAN | VPD | 0.85 | 1 | 18 | 77 | 1 | 1 | 1.3 | 1.6 |
| Kenin-K | 2013 | PAN | Tmin | 0.832 | 1 | 33 | 52 | 30.6 | 30.7 | 32 | 33.7 |
| Kenin-K | 2013 | PAN | Trange | 0.8 | 0.994 | 26 | 45 | 7.9 | 8 | 8.5 | 9.6 |
| Kenin-K | 2013 | PAN | solarRad | 0.782 | 0.865 | 34 | 53 | 756.7 | 748.4 | 748.2 | 729.3 |
| Kenin-K | 2013 | PAN | hum | 0.774 | 0.998 | 6 | 105 | 78.2 | 77.4 | 72.8 | 67.1 |
|  |  |  |  |  |  |  |  |  |  |  |  |
| Kenin-K | 2013 | PED | SVP | 0.714 | 0.99 | 12 | 51 | 0.2 | 0.2 | 0.2 | 0.2 |
| Kenin-K | 2013 | PED | photothermal | 0.713 | 1 | 21 | 60 | 9447.8 | 9132.6 | 9683.7 | 9869.8 |
| Kenin-K | 2013 | PED | DD | 0.713 | 0.992 | 12 | 51 | 763.3 | 741.5 | 788.6 | 804.8 |
| Kenin-K | 2013 | PED | VPD | 0.708 | 0.994 | 11 | 50 | 1.1 | 0.9 | 1.1 | 1.2 |
| Kenin-K | 2013 | PED | Tmin | 0.674 | 0.988 | 9 | 48 | 31.2 | 30.4 | 32.1 | 32.5 |
|  |  |  |  |  |  |  |  |  |  |  |  |
| Kenin-K | 2013 | YIELD | photoperiod | 0.714 | 0.807 | 1 | 20 | 12.7 | 12.6 | 12.6 | 12.4 |
| Kenin-K | 2013 | YIELD | solarRad | 0.664 | 0.727 | 1 | 60 | 2266.2 | 2254.1 | 2256.4 | 2220.1 |
| Kenin-K | 2013 | YIELD | hum | 0.502 | 0.996 | 5 | 24 | 75.4 | 80.3 | 75.7 | 82.1 |
| Kenin-K | 2013 | YIELD | hSun | 0.493 | 0.98 | 1 | 20 | 154.4 | 131.4 | 147.7 | 126.4 |
| Kenin-K | 2013 | YIELD | Trange | 0.491 | 0.867 | 57 | 76 | 8.4 | 10.2 | 9.6 | 12 |

**Table S11**: QTL parental allele effects detailed statistics (significant effect, QTLxE effect, QTLxEC effect)

In each configuration: sub-population (e.g. GR2012) x trait (e.g. FLAG), the QTL scan, gave us a number of significant QTL positions. Each of those QTL positions is defined by N_parent – 1 (central parent set as reference) alleles.

For each configuration, we determined the proportion of parental allele that have a significant effect, that significantly interact with the environment, and that significantly interact with at least one environmental covariate.

| ***Total number of parental alleles at the QTL positions*** | | | | | | | | | |
| --- | --- | --- | --- | --- | --- | --- | --- | --- | --- |
|  | **FLAG** | **PH** | **NODE_N** | **NODE_L** | **PED** | **PAN** | **GWGH** | **YIELD** | **Total/av.** |
| GR2012 | 78 | 39 | 52 | 26 | 78 | 65 | 36 | 39 | 413 |
| GR2013 | 32 | 32 | 8 | 16 | 16 | 8 | 0 | 16 | 128 |
| KK2012 | 30 | 10 | 10 | 15 | 15 | 10 | 0 | 5 | 95 |
| KK2013 | 12 | 12 | 12 | 0 | 12 | 6 | 0 | 6 | 60 |
| Lata | 48 | 24 | 24 | 12 | 24 | 0 | 72 | 48 | 252 |
| Total | 200 | 117 | 106 | 69 | 145 | 89 | 108 | 114 | 948 |
| ***Proportion of QTL parental allele with a significant effect*** | | | | | | | | | |
| GR2012 | 0.4 | 0.67 | 0.38 | 0.81 | 0.41 | 0.32 | 0.33 | 0.54 | 0.48 |
| GR2013 | 0.38 | 0.5 | 0.5 | 0.69 | 0.62 | 0.62 | NA | 0.38 | 0.53 |
| KK2012 | 0.5 | 0.6 | 0.5 | 0.67 | 0.4 | 0.4 | NA | 0.8 | 0.55 |
| KK2013 | 0.67 | 0.5 | 0.67 | NA | 0.58 | 0.5 | NA | 0.17 | 0.51 |
| Lata | 0.58 | 0.33 | 0.62 | 0.33 | 0.58 | NA | 0.43 | 0.42 | 0.47 |
| Average | 0.5 | 0.52 | 0.54 | 0.62 | 0.52 | 0.46 | 0.38 | 0.46 | 0.51 |
| ***Proportion of QTL parental allele with a significant environmental interaction*** | | | | | | | | | |
| GR2012 | 0.26 | 0.38 | 0.15 | 0.46 | 0.28 | 0.15 | 0.19 | 0.44 | 0.29 |
| GR2013 | 0.16 | 0.31 | 0.38 | 0.38 | 0.31 | 0.25 | NA | 0.25 | 0.29 |
| KK2012 | 0.27 | 0.4 | 0.4 | 0.27 | 0.2 | 0.1 | NA | 0.4 | 0.29 |
| KK2013 | 0.17 | 0.33 | 0.25 | NA | 0.25 | 0.33 | NA | 0.17 | 0.25 |
| Lata | 0.27 | 0.17 | 0.12 | 0 | 0.21 | NA | 0.19 | 0.25 | 0.17 |
| Average | 0.22 | 0.32 | 0.26 | 0.28 | 0.25 | 0.21 | 0.19 | 0.3 | 0.26 |
| ***Proportion of QTL parental allele with at least one EC interaction*** | | | | | | | | | |
| GR2012 | 0.17 | 0.33 | 0.09 | 0.21 | 0.21 | 0.06 | NA | 0.24 | 0.16 |
| GR2013 | 0.08 | 0.25 | 0.33 | 0.33 | 0.22 | 0.11 | NA | 0.17 | 0.21 |
| KK2012 | 0.19 | 0.17 | 0.33 | 0.11 | 0.17 | 0.08 | NA | 0 | 0.15 |
| KK2013 | 0.14 | 0.21 | 0.14 | NA | 0.07 | 0 | NA | 0.14 | 0.12 |
| Average | 0.15 | 0.24 | 0.22 | 0.22 | 0.17 | 0.06 | NA | 0.14 | 0.16 |

**Table S12**: Number of significant QTL parental allele by environmental covariable interaction

| Category |  | FLAG | PH | NODE_N | NODE_L | PED | PAN | YIELD | Total |
| --- | --- | --- | --- | --- | --- | --- | --- | --- | --- |
| Atmospheric | rain | 21 | 0 | 0 | 5 | 16 | 1 | 6 | 49 |
|  | hum | 5 | 27 | 13 | 0 | 18 | 0 | 11 | 74 |
|  | VPD | 0 | 9 | 0 | 6 | 21 | 1 | 3 | 40 |
|  | SVP | 0 | 0 | 0 | 6 | 4 | 5 | 0 | 15 |
|  | ETP | 0 | 0 | 0 | 5 | 0 | 4 | 0 | 9 |
|  | PETP | 0 | 0 | 0 | 5 | 16 | 0 | 0 | 21 |
| Temperature | Tmin | 0 | 0 | 0 | 8 | 21 | 5 | 3 | 37 |
|  | Tmax | 0 | 1 | 0 | 0 | 0 | 0 | 0 | 1 |
|  | Trange | 23 | 25 | 11 | 0 | 3 | 1 | 10 | 73 |
|  | DD | 0 | 0 | 0 | 6 | 4 | 5 | 0 | 15 |
|  | FRUE | 0 | 0 | 0 | 4 | 0 | 4 | 0 | 8 |
| Radiation | hSun | 23 | 17 | 13 | 4 | 2 | 1 | 7 | 67 |
|  | photoperiod | 24 | 26 | 12 | 0 | 0 | 1 | 8 | 71 |
|  | solarRad | 23 | 28 | 10 | 0 | 1 | 1 | 3 | 66 |
| Photothermal | photothermal | 0 | 1 | 0 | 6 | 4 | 1 | 0 | 12 |
|  | Total | 119 | 134 | 59 | 55 | 110 | 30 | 51 | 558 |

**Supplemental Methods**

**Method S1:** Approximate mixed model computation and QTL test statistic

To reduce the computational demand needed to perform the QTL scan we implemented an approximate mixed model computation similar to the generalized least square strategy implemented in Kruijer et al. (2015). The procedure consists of estimating a general VCOV ($\hat{V}$) using model 2 without the tested QTL position, which means estimating the VCOV of model 2 without the QTL term for the SIM scan and the same model with selected cofactors for the CIM scan. The statistical significance of the tested QTL positions and the different allelic effect was obtained by using $\hat{V}$ to get the following Wald statistic $W_{Q}=\beta^{T}{V(\beta)}^{-1}\beta$, where $\beta={(X^{T}V^{-1}X)}^{-1}X^{T}V^{-1}y$, $V\left( \beta\right)= {(X^{T}V^{-1}X)}^{-1}$, $X$ represents the fixed effect matrix including the QTL position, and $y$the vector of phenotypic values. $W_{Q}$ follows a chi-square distribution with degree of freedom equal to the number of tested QTL allelic effects ($\left( N_{par}-1 \right)*N_{env}$ for the main QTL term).

**Method S2**: Diversity tree construction methodology

This analysis was based on 137’003 SNPs common to the SAP (Boatwright et al. 2022) and SGT (<https://www.globalsorghuminitiative.org/>) panels. From a merge of these 2 studies, we excluded all wild accessions and kept SNP with less than 20% of missing data and with a Minor Allele Frequency (MAF) higher than 5%. To lighten the matrix without losing too much precision, we pruned with bcftools (Danecek et al, 2021) with these parameters : windows (-w) 1000 and r² (-m) bigger than 0.8. The software Darwin ([https://darwin.cirad.fr/](https://darwin.cirad.fr/index.php)) was used to calculate the dissimilarity matrix and draw the tree (NJ method).

**Method S3**: Diversity coverage computation

To calculate the diversity coverage, we used a reference marker set of 2’877’109 SNPs obtained after the combination of genetic information from the SAP (Boatwright et al. 2022), SGT (<https://www.globalsorghuminitiative.org/>), AG (AdpatGrass, Jean-Christophe Glaszmann personal communication), and CSP (<https://genome.jgi.doe.gov/portal/AComAofSorghum/AComAofSorghum.info.html>) panels for 1443 individuals. The reference markers contained less than 20% missing values. We calculated the percentage of SNPs with at least one parent homozygous for the minor allele in the parents from the sorghum WCA-BCNAM and the sorghum US NAM populations (Bouchet et al. 2017), after excluding the marker with less than 5, 2.5, and 1% missing values.

**References**

Bouchet, S., Olatoye, M. O., Marla, S. R., Perumal, R., Tesso, T., Yu, J., ... & Morris, G. P. (2017). Increased power to dissect adaptive traits in global sorghum diversity using a nested association mapping population. *Genetics*, *206*(2), 573-585.

Boatwright, J.L., Sapkota, S., Jin, H., Schnable, J.C., Brenton, Z., Boyles, R. and Kresovich, S. (2022), Sorghum Association Panel whole-genome sequencing establishes cornerstone resource for dissecting genomic diversity. Plant J, 111: 888-904. <https://doi.org/10.1111/tpj.15853>

Danecek P, Bonfield JK, *et al*. Twelve years of SAMtools and BCFtools. *Gigascience* (2021) 10(2):giab00

Li, J., & Ji, L. (2005). Adjusting multiple testing in multilocus analyses using the eigenvalues of a correlation matrix. *Heredity*, *95*(3), 221-227.

Sparks, A. H. (2018). nasapower: a NASA POWER global meteorology, surface solar energy and climatology data client for R. *Journal of Open Source Software*, *3*(30), 1035.
